# Supplementary material for: Interference with ERK-dimerization at the nucleocytosolic interface targets pathological ERK1/2 signaling without cardiotoxic side-effects
Source: Nat Commun. 2020 Apr 7;11:1733. doi: 10.1038/s41467-020-15505-4 (PMC7138859; doi:10.1038/s41467-020-15505-4)
Supplement: Supplementary file 1 — Supplementary Information [file 41467_2020_15505_MOESM1_ESM.pdf]

Supplementary Information accompanying:

Interference with ERK-dimerization at the nucleocytosolic interface targets pathological ERK1/2 signaling without cardiotoxic side-effects

Angela Tomasovic#, Theresa Brand#, Constanze Schanbacher#, Sofia Kramer, Martin W. Hümmert, Patricio Godoy, Wolfgang Schmidt-Heck, Peter Nordbeck, Jonas Ludwig, Susanne Homann, Armin Wiegering, Timur Shaykhutdinov, Christoph Kratz, Ruth Knüchel, Hans-Konrad Müller-Hermelink, Andreas Rosenwald, Norbert Frey, Jutta Eichler, Dobromir Dobrev, Ali El-Armouche, Jan G. Hengstler, Oliver J. Müller, Karsten Hinrichs, Friederike Cuello, Alma Zernecke, Kristina Lorenz*

Supplementary Figures and Tables

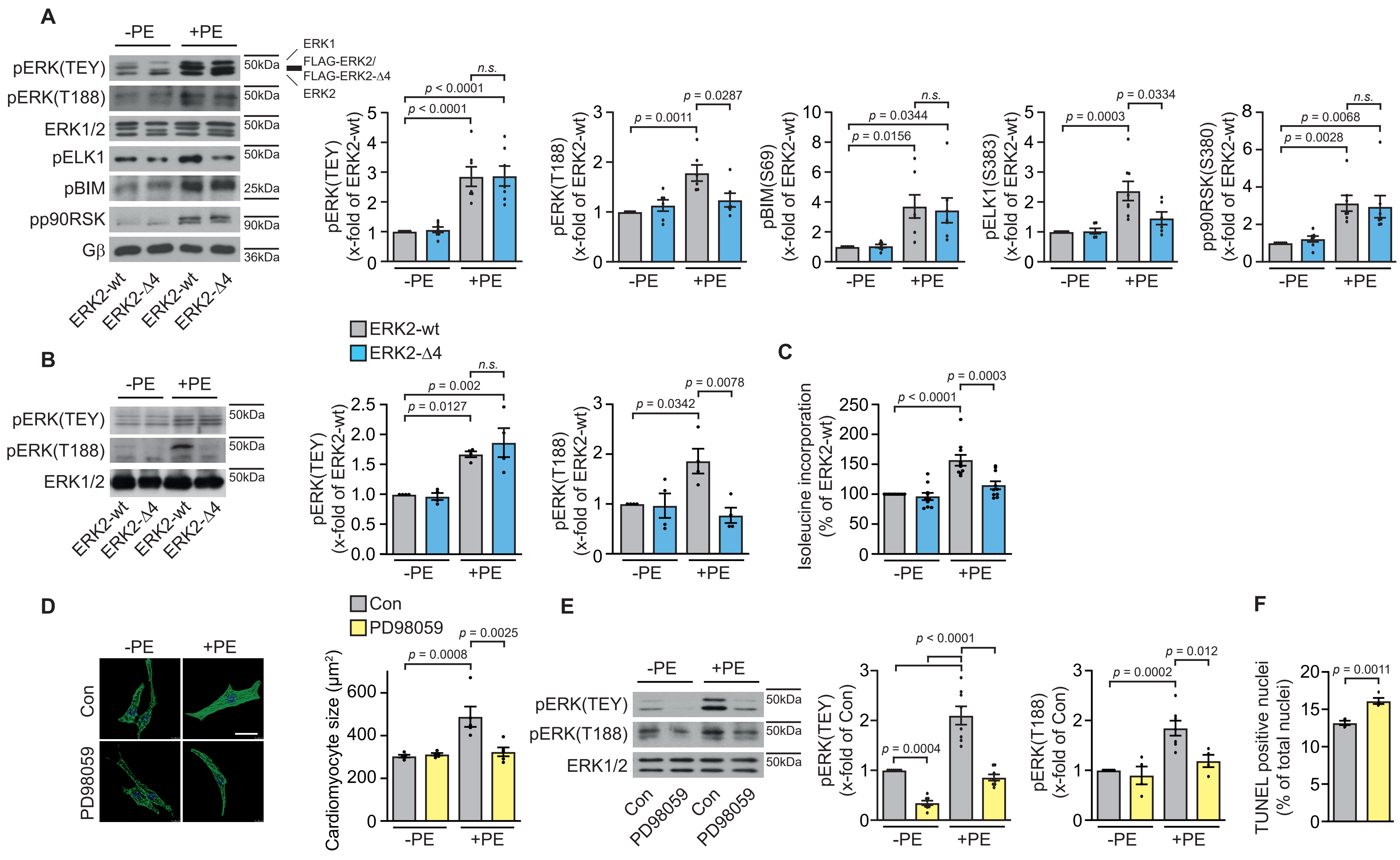

Supplementary Figure1

Supplementary Figure 1

Cells transduced with monomeric ERK2 show reduced ERK_{T188}-phosphorylation but normal ERK activation. (**A-C**) NRCM were transduced with Flag-ERK2-wt (ERK2-wt) or Flag-$ERK2_{\Delta 174\text{-}177}$ (ERK2-Δ4) and stimulated as indicated. (**A**) Immunoblot analysis of pERK(TEY) (n=7) and pERK(T188) (n=6) and phosphorylation levels of nuclear (pELK1[S383], n=8 for ERK2-wt and n=6 for ERK2-Δ4) and cytosolic (pBIM[S69], n=7; pp90RSK[S380], n=7) ERK-targets in the absence (-PE) and presence of phenylephrine stimulation (+PE; 4µM, 10min). Shown are quantitative analyses and representative western blots. Gβ was used as additional loading control. **(B)** Immunoblot analysis of pERK(TEY) and pERK(T188) in the absence (-PE) and presence of phenylephrine stimulation (+PE; 4µM, 24h) (n=4). Shown are quantitative analyses and representative western blots. (**C**) [^{3}H]Isoleucine incorporation assay (n=10). Cells were stimulated with PE as indicated (4µM, 24h). (**D**) Representative pictures of phalloidin-stained NRCM and cell area analysis of NRCM pretreated with **PD98059** (30µM, 1h) and stimulated with PE as indicated (4µM, 24h; n=5 and 40 cells per group and experiment; scale bar 20µm). (**E**) Western blot analyses of NRCM pretreated with **PD98059** (30µM, 1h) and stimulated with PE (4µM, 10min) as indicated for ERK(TEY)- and ERK(T188)-phosphorylation. Shown are quantitative analyses and representative western blots (pERK(TEY), n=8; pERK(T188), Con±PE n=7 and PD98059±PE n=4). (**F**) TUNEL assay analysis of NRCM after H_2O_2 treatment (100µM, 1h; n=4 of at least 500 cells per experiment and group). Error bars are mean±s.e.m.; n numbers represent biologically independent experiments. For statistical analysis ordinary one-way ANOVA (A-E) or unpaired and two-sided Student's *t*-test (F) were applied, for (B) the Tukey test and for (A,C,D,E) Bonferroni was applied as *post hoc* test. Source data are provided as a Source Data file.

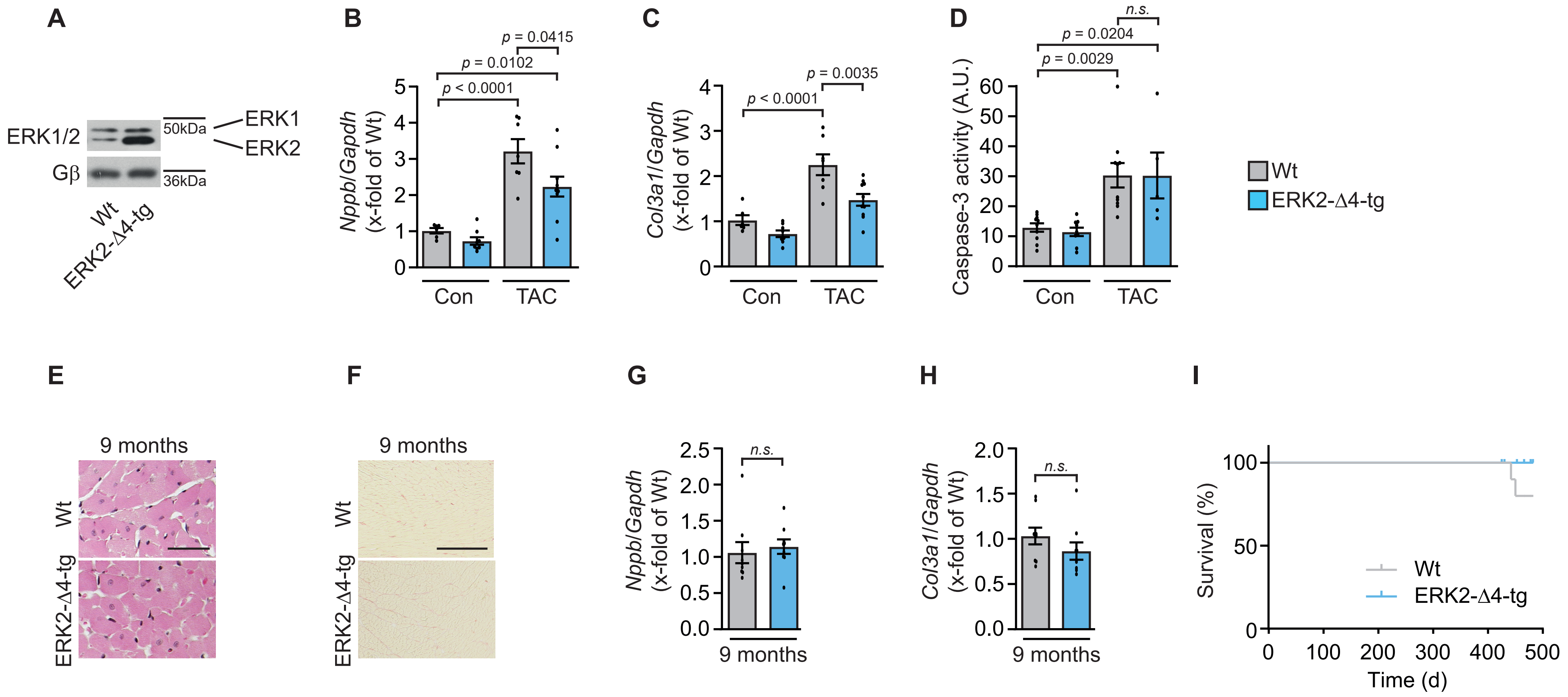

Supplementary Figure2

Supplementary Figure 2

Monomeric ERK2 attenuates pathological cardiac remodeling but not physiological heart growth. (**A**) Representative western blot of ERK1/2 expression levels in wild-type (Wt) and ERK2$_{\Delta174\text{-}177}$ (ERK2-Δ4-tg) transgenic mice. Gβ was used as an additional loading control. Western blot was reproduced five times with similar result. (**B**-**D**) Wt and ERK2-Δ4-tg mice were analyzed for apoptosis and markers of remodeling before (Con) and 6 weeks after transverse aortic constriction (TAC). (**B**,**C**) mRNA expression levels of brain natriuretic peptide (*Nppb;* **B**) and collagen type III alpha 1 (*Col3a1;* **C**) normalized to glycerinaldehyde-3-phosphate dehydrogenase (*Gapdh*). Wt Con, n=6; ERK2-Δ4-tg Con, n=8; Wt TAC, n=7; ERK2-Δ4-tg TAC, n=10 mice per group; n represent numbers of mice measured in triplicates. (**D**) Caspase-3 activity in heart lysates (Wt Con, n=10; ERK2-Δ4-tg Con, n=9; Wt TAC, n=10; ERK2-Δ4-tg TAC, n=5 mice per group). (**E-I**) 9 months old Wt and ERK2-Δ4-tg were analyzed for cardiomyocyte size, interstitial fibrosis, markers of cardiac remodeling and survival. (**E,F**) Shown are representative histological H&E (scale bar 50µm, **E**) and Sirius Red-stained (scale bar 200µm, **F**) sections of left ventricular myocardium. Histological stainings were performed in Wt (n=9) and ERK2-Δ4-tg (n=10). Quantifications: Supplementary Table 2. (**G**,**H**) mRNA expression levels of *Nppb* (**G**) and *Col3a1* (**H**) normalized to *Gapdh* (n=9; n represents the numbers of mice per group measured in triplicates). (**I**) Kaplan-Meier survival curves of Wt (n=14) and ERK2-Δ4-tg (n=25 mice per group). Error bars are mean±s.e.m.. For statistical analysis ordinary one-way ANOVA (B-D) and Bonferroni as *post hoc* test or an unpaired and two-sided Student's *t*-test (G,H) were applied. For (I) an survival curve comparison was applied. Source data are provided as a Source Data file.

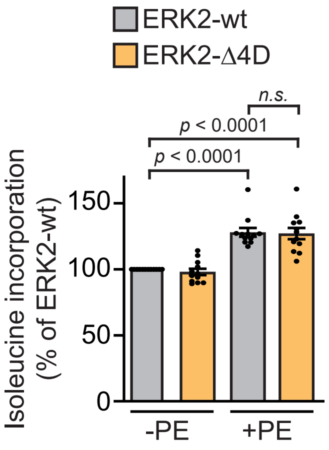

Supplementary Figure3

Supplementary Figure 3

ERK2-Δ4D shows a comparable hypertrophic response to phenylephrine as ERK2-wt. [^{3}H]Isoleucine incorporation assay of NRCM transduced with Flag-tagged ERK2-wt or Flag-ERK2$_{\Delta174\text{-}177,T188D}$ (ERK2-Δ4D) stimulated with phenylephrine (PE; 4µM, 30h; n=12 experiments). Error bars are mean±s.e.m., n numbers represent biologically independent experiments. For statistical analysis ordinary one-way ANOVA and Bonferroni as *post hoc* test was applied. Source data are provided as a Source Data file.

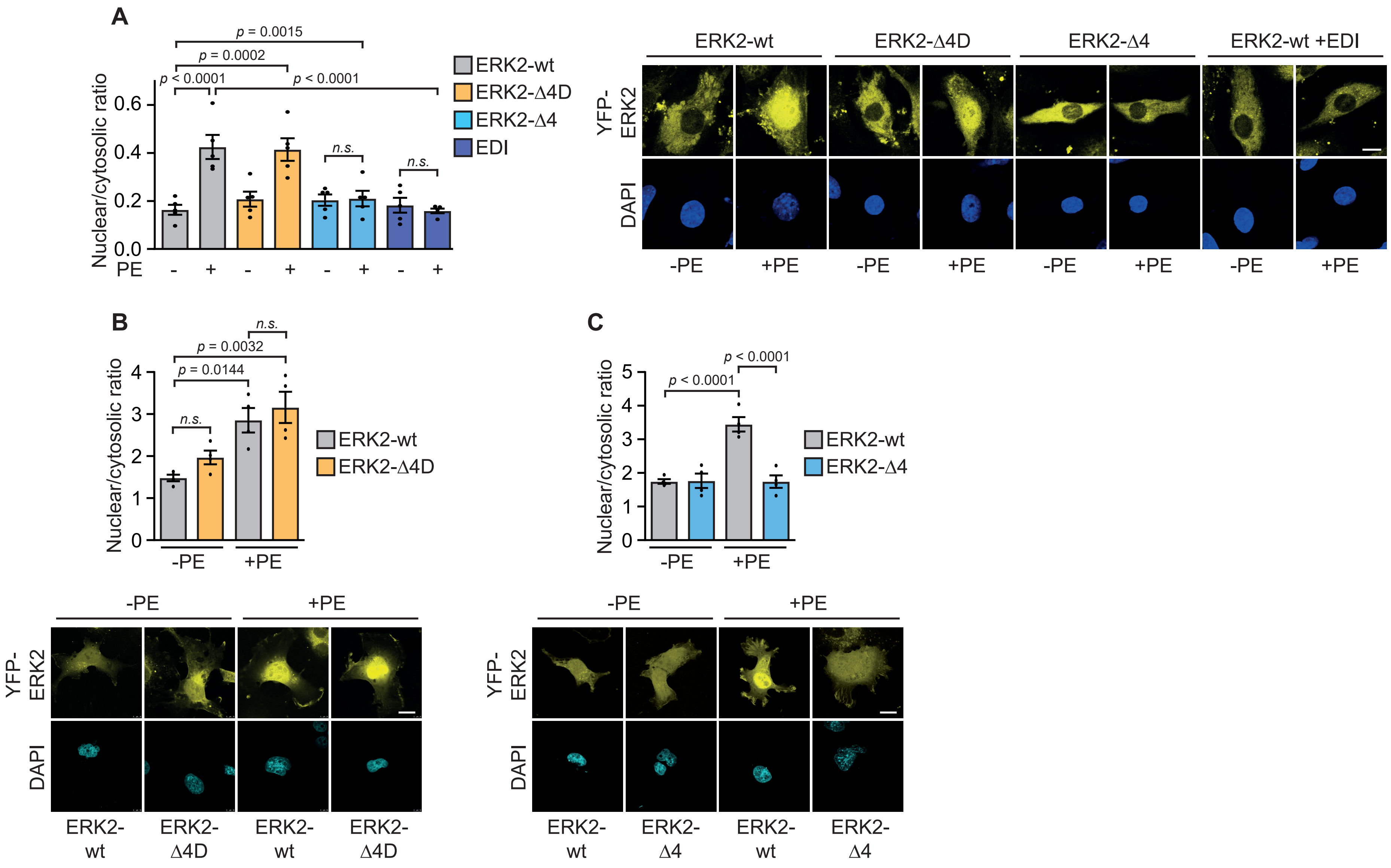

Supplementary Figure4

Supplementary Figure 4

ERK$_{T188}$-phosphorylation is essential for nuclear accumulation of ERK2 and can be prevented by interference with ERK dimerization. (A) Quantitative analysis of nuclear-to-cytosolic ratios of YFP-tagged ERK2-wt (ERK2-wt), YFP-ERK2$_{\Delta 174-177}$ (ERK2-Δ4) and YFP-ERK2$_{\Delta 174-177,T188D}$ (ERK2-Δ4D) and ERK2-wt+EDI in NRCM. Cells were stimulated with PE (4µM, 10min) as indicated. (30-100 cells per group of n=5). For illustration, representative confocal images displaying distribution of YFP-tagged ERK2 constructs (yellow) and DAPI-stained cell nuclei (blue) (scale bar 10µm) are shown. (**B,C**) Analysis of nuclear-to-cytosolic ratios of YFP-ERK2-wt, YFP-ERK2-Δ4D and YFP-ERK2-Δ4 in COS7 cells. Cells were stimulated with PE (10µM, 10min) as indicated. Shown are representative confocal images displaying distribution of YFP-tagged ERK2 constructs (yellow) and DAPI-stained cell nuclei (blue) (scale bar 10µm) and the respective quantitative analysis (60 cells per group of n=4). Error bars are mean±s.e.m.; n numbers represent biologically independent experiments. For statistical analysis ordinary one-way ANOVA and for (B,C) Bonferroni as *post hoc* test and for (A) Tukey test was applied. Source data are provided as a Source Data file.

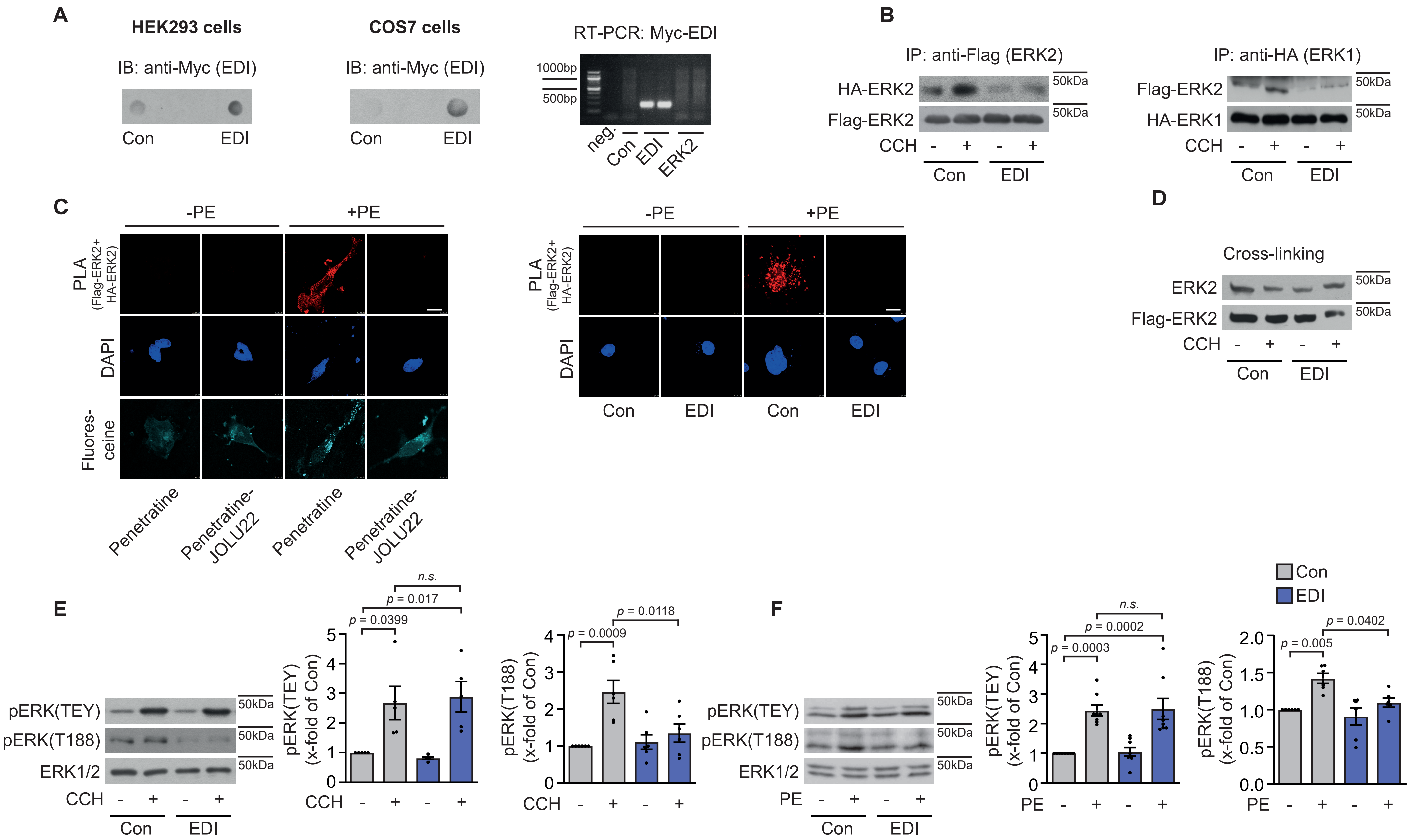

Supplementary Figure5

Supplementary Figure 5

Characterization of a peptide that interferes with ERK dimerization. (**A**) Analysis of myc-ERK2$_{309-357}$ (EDI) expression in HEK293 (left panel) and COS7 cells (middle panel) by dot blot analysis or reverse transcriptase polymerase chain reaction (RT-PCR; right panel). Comparable transfection conditions were used for experiments in Supplementary Figure 5B-F and Supplementary Figure 6C. Dot blots and RT-PCR were reproduced three times with similar results. (**B**) Shown are representative co-immunoprecipitations performed using HEK293 cells transfected with HA- and Flag-ERK2 (left panel; n=5) or HA-ERK1 and Flag-ERK2 (right panel; n=3) and either pcDNA3 (Con) or myc-EDI (EDI). Flag-ERK2 or HA-ERK1were immunoprecipitated (IP) and analyzed for co-immunoprecipitated HA-ERK2 or Flag-ERK2 in response to carbachol stimulation (CCH; 100µM, 10min) as indicated. (**C**) Representative confocal pictures of proximity ligation assays in COS7 cells. Cells were transfected with HA- and Flag-tagged ERK2-wt, and treated with either fluorescein-tagged penetratin as control or with fluorescein-tagged penetratin fused to ERK2$_{328-352}$ (JOLU22, left panel) or cells were transfected with HA- and Flag-tagged ERK2-wt and either myc-ERK$_{309-357}$ (EDI) or pcDNA3 as a control (Con) and stimulated with phenylephrine (PE; 4µM, 10min; n=4; scale bar 10µm). (**D**) Representative cross-linking experiment using HEK293 lysates, paraformaldehyde as crosslinking reagent and carbachol (CCH; 300µM, 10min) as stimulus for ERK1/2 activation. Transfection was performed as described for (B, left panel). ERK2 and its mobility shift were detected using anti-Flag and anti-ERK1/2 antibodies. Cross-linking experiment was reproduced six times with similar result. (**E**,**F**) Representative western blots and western blot analyses of ERK-phosphorylation levels in HEK293 (**E**) or COS7 (**F**) cells transfected with Flag-ERK2 and either pcDNA3 (Con) or myc-EDI (EDI) in the absence and presence of CCH (**E**; 300µM, 10min; pERK(TEY), n=5; pERK(T188), n=6) or PE (**F**; 4µM, 10min; pERK(TEY), n=8; pERK(T188), n=6). Error bars are mean±s.e.m.; n numbers represent biologically independent

experiments. For statistical analysis ordinary one-way ANOVA and Bonferroni as *post hoc* test was applied. Source data are provided as a Source Data file.

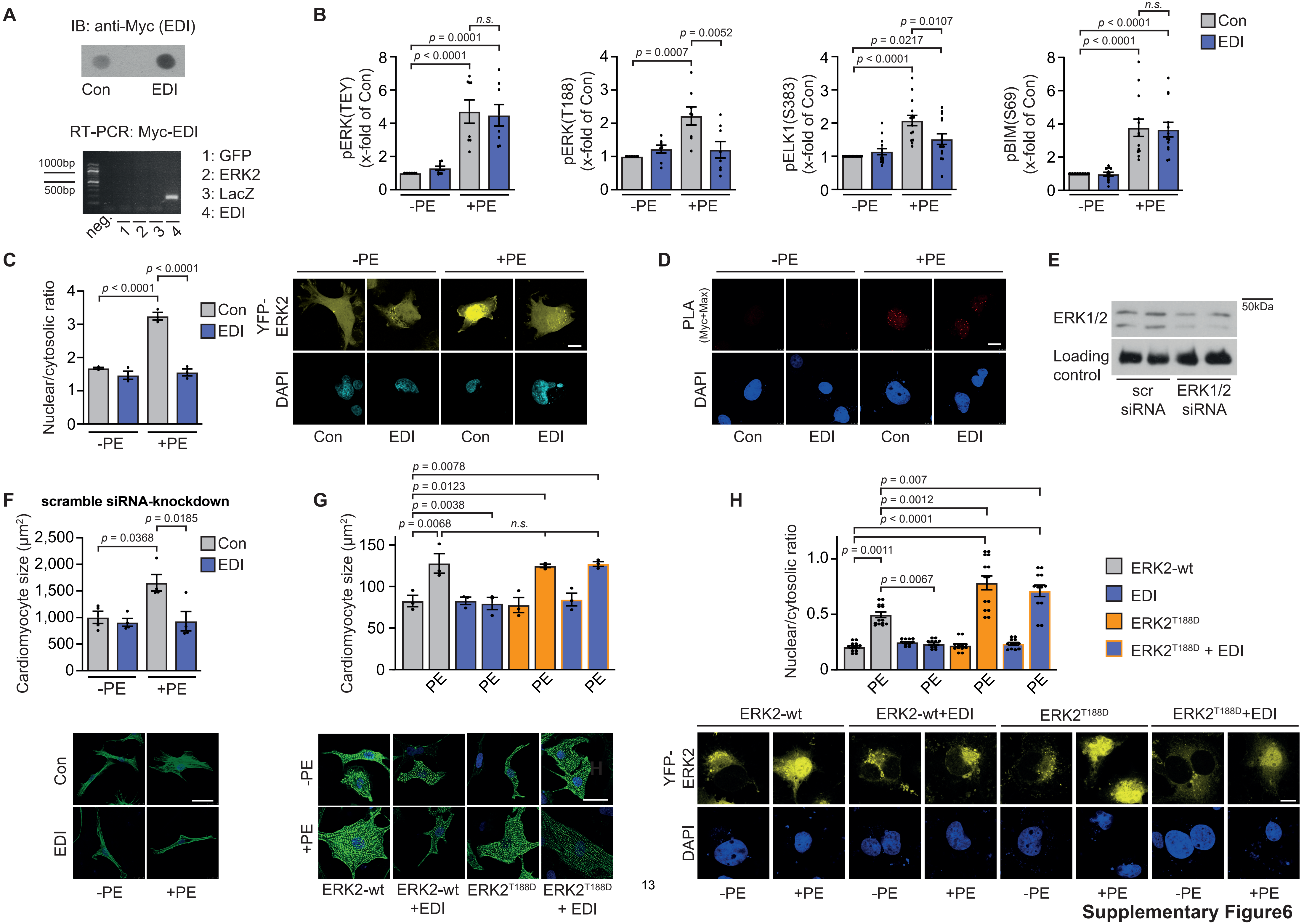

Supplementary Figure6

Supplementary Figure 6

Functional analysis of myc-EDI. (**A**) Myc-EDI expression in NRCM detected by dot blot analysis and RT-PCR. NRCM were transduced with GFP (Con), myc-EDI, LacZ or Flag-ERK2-wt. Dot blot was reproduced 4 times with similar result and RT-PCR three times. (**B**) NRCM were transduced with LacZ (Con) or EDI virus, stimulated with PE (4µM, 10min) and analyzed for phosphorylation of ERK (pERK[TEY] and pERK[T188]), nuclear ELK1 (pELK[S383]) and cytosolic BIM (pBIM[S69]) by western blot (n=8, for pBIM n=13). (**C**) Nucleocytosolic distribution of YFP-ERK2-wt (yellow) in COS7 cells in the presence of EDI and PE (10µM, 10min); DAPI-stained nuclei (blue). Shown are representative pictures and quantitative analyses (60-70 cells per group of n=3; scale bar 10µm). (**D**) Representative confocal PLA pictures in COS7 detecting the interaction of Myc and Max in the absence and presence of EDI and PE (4µM, 10min; 15-20 cells per group and experiment of n=4; scale bar 10µm). (**E**) siRNA-mediated knock-down of ERK1/2 (ERK1/2 siRNA) in NRCM and scrambled siRNA (scr siRNA) analyzed by western blot (n=4). (**F**) NRCM size after transduction with control virus or EDI and treatment with scr siRNA and PE (4µM, 24h). Representative confocal pictures of phalloidin-stained cells and quantifications (55-87 cells per group of n=4; scale bar 20µm). (**G**) NRCM were transduced with Flag-tagged ERK2-wt (ERK2-wt) or Flag-tagged $ERK2_{T188D}$ and EDI if indicated, and treated with PE (4µM, 24h). Representative phalloidin-stained NRCM (scale bar 25µm) and analyses of cell sizes (70-120 cells per group and experiment of n=3). (**H**) COS7 cells were transfected with YFP-tagged ERK2-wt (ERK2-wt), YFP-$ERK2_{T188D}$ ($ERK2_{T188D}$) and EDI as indicated and stimulated with PE (10µM, 10min). Representative confocal images of YFP-ERK2 (yellow), DAPI-stained nuclei (blue; scale bar 10µm) and respective quantitative analyses (ERK2-wt, ERK2-wt+PE, $ERK2_{T188D}$, $ERK2_{T188D}$+PE, n=7; EDI, EDI+PE, $ERK2_{T188D}$+EDI, $ERK2_{T188D}$+EDI+PE, n=6; 30-50 cells per group of the indicated number of experiments). Error bars are mean±s.e.m.; n numbers represent biologically independent experiments. For statistical analysis ordinary one-

way ANOVA and as *post hoc* test Bonferroni was applied for (B,C,F) and for (G,H) the Tukey test. Source data are provided as a Source Data file.

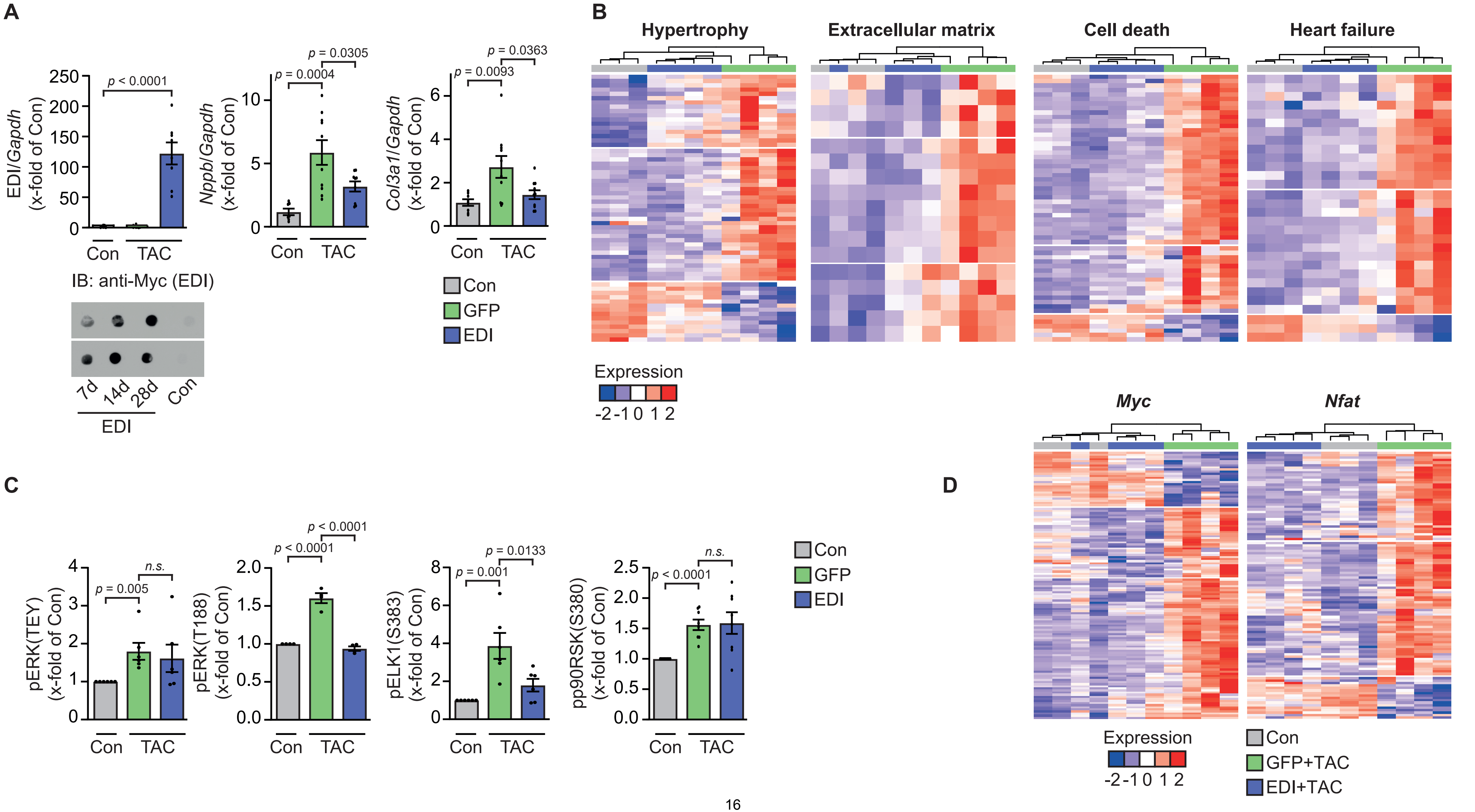

Supplementary Figure7

Supplementary Figure 7

AAV9-EDI gene therapy reduces cardiac remodeling in response to TAC. (**A-D**) Cardiac characterization of wild-type mice before (Con; 8-week old C57/BL6 mice) and 4 weeks after transverse aortic constriction (TAC) and treatment with an adeno-associated virus serotype 9 (AAV9) vector encoding EDI (EDI) or eGFP as control (GFP) under the control of the CMV-enhanced myosin light chain promotor fragment (CMV-MLC0.26kb). (**A**) mRNA expression of myc-EDI (Con, n=7; GFP+TAC, n=11; EDI+TAC, n=8), brain natriuretic peptide (*Nppb*: Con, n=7; GFP+TAC, n=10; EDI+TAC, n=9) and collagen type III alpha 1 (*Col3a1*: Con, n=8; GFP+TAC, n=10; EDI+TAC, n=10) normalized to glycerinaldehyde-3-phosphate dehydrogenase (*Gapdh*) and dot blot immunoblot analysis of myc-EDI expression (lower panel) for the indicated time points after AAV9-EDI injection (-TAC). n represents number of mice measured in triplicates. (**B**) Microarray gene expression analysis of left ventricular myocardial tissue (Con n=3, GFP and EDI n=4 mice per group). Heatmap visualization of transcriptional changes of genes related to hypertrophy, extracellular matrix, cell death and heart failure. For gene list and changes refer to Supplementary Tables 7-10. (**C**) Western blot analyses of heart lysates: pERK(TEY) (n=6) and pERK(T188) (n=4); pELK1(S383) (n=6; nuclear ERK-target) and pp90RSK(S380) (n=8; cytosolic ERK-target). (**D**) Heatmap visualization of transcriptional changes of genes related to *Nfat*- or *Myc*-signaling. Same Microarray gene expression analysis as in B. For gene list and changes refer to Supplementary Tables 11 and 12. Error bars are mean±s.e.m.; n numbers represent biologically independent animals. For statistical analysis ordinary one-way ANOVA and Tukey as *post hoc* test was applied. Source data are provided as a Source Data file.

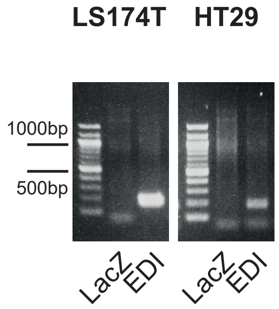

Supplementary Figure8

Supplementary Figure 8

Detection of EDI in tumor cells. LS174T and HT29 colon cancer cells adenovirally transduced with LacZ or EDI. Detection of EDI-mRNA expression by RT-PCR. Shown are representative experiments of n=4. n numbers represent biologically independent experiments. Source data are provided as a Source Data file.

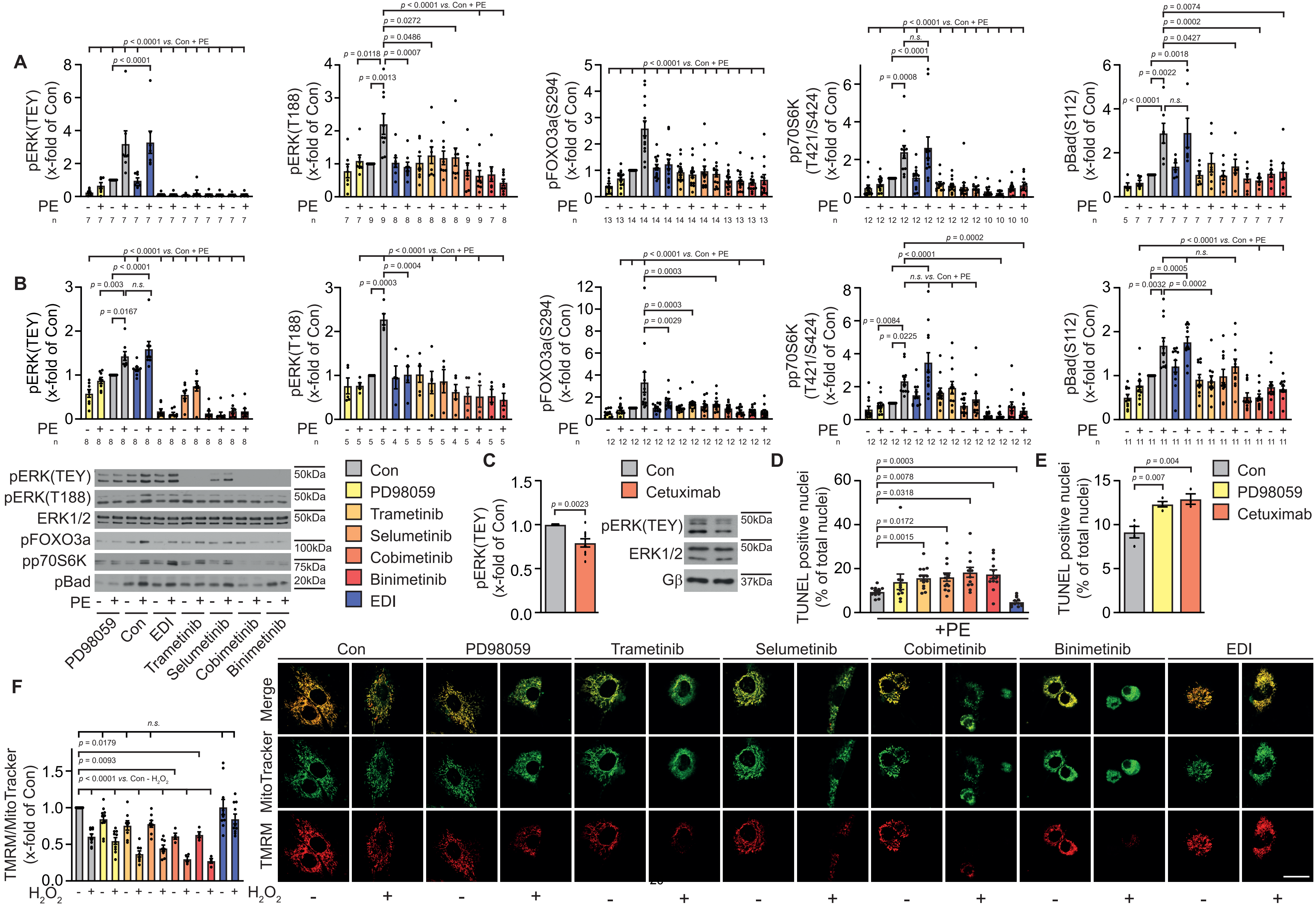

Supplementary Figure9

Supplementary Figure 9

Analysis of PD98059, newer generation MEK-inhibitors, cetuximab and EDI in cardiomyocytes. (**A,B**) NRCM (A) and H9c2 cells (B) were adenovirally transduced with EDI or LacZ (Con) and pretreated with **PD98059** (30µM), trametinib (15µM), selumetinib (1µM), cobimetinib (5µM) and binimetinib (10µM) for 1h as indicated. Shown are quantitative analyses of representative blots of Fig. 6A (A, NRCM) and H9c2 cells including representative blots (B) of pERK(TEY) and pERK(T188) and phosphorylation levels of nuclear (pFoxo3a[S294]) and cytosolic (pp70S6K[T421/S424]; pBad[S112]) ERK-targets in the absence and presence of PE (4µM, 10min). n numbers are given in the figures. (**C**) Representative western blots and western blot analysis of pERK(TEY) of untreated (Con) or cetuximab-treated NRCM (0.2 µg/µl, 24h) n=9. **(D, E)** Analysis of TUNEL-positive cardiomyocyte nuclei. (**D**) NRCM untreated (Con, n=13) or treated with **PD98059** (30µM, n=13), trametinib (15µM, n=14), selumetinib (1µM, n=13), cobimetinib (5µM, n=12), binimetinib (10µM, n=12) for 1h or transduced with EDI (n=11) were stimulated with PE (30µM, 15min, ≥100 cells per experiment and group). (**E**) NRCM treated with cetuximab (0.2µg/µl, 24h) or **PD98059** (30µM, 1h) and stimulated with H_2O_2 (100µM, 1h; Con, **PD98059**, n=4; cetuximab, n=3, ≥100 cells per experiment and group). (**F**) H9c2 cells were adenovirally transduced with EDI (n=10) or LacZ (Con, n=10) and pretreated with **PD98059** (30µM, n=10), trametinib (15µM, n=8), selumetinib (1µM, n=8), cobimetinib (5µM, n=4) and binimetinib (10µM, n=4) for 1h as indicated. Assessment of mitochondrial membrane potential in the presence or absence of H_2O_2 stimulation (100µM, 15min). Shown are representative NRCM stained with TMRM and MitoTrackerGreen (scale bar 25µm) and respective quantifications (15-40 cells per experiment). Error bars are mean±s.e.m.; n numbers represent biologically independent experiments. For statistical analysis ANOVA was applied. For (A,B,E,F) Tukey test, for (C) paired two-sided Student's *t*-test was used. Source data are provided as a Source Data file.

Figure 2D

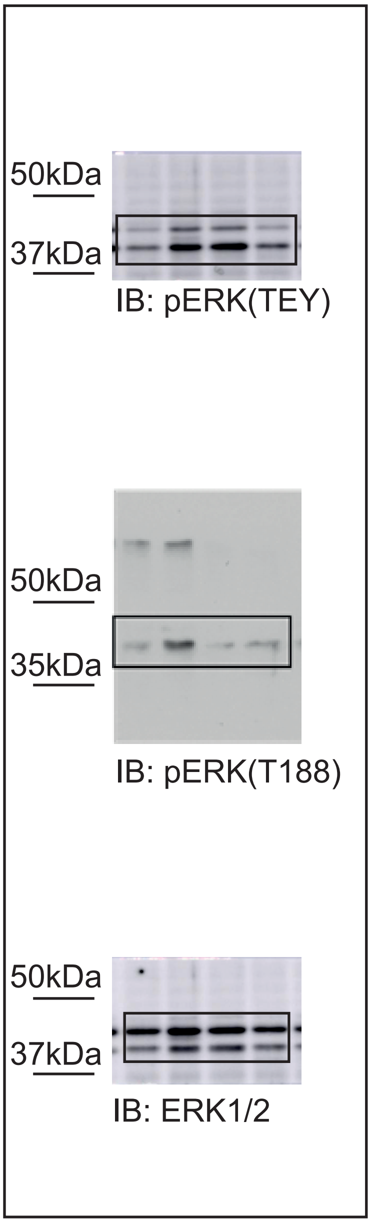

Figure 3C

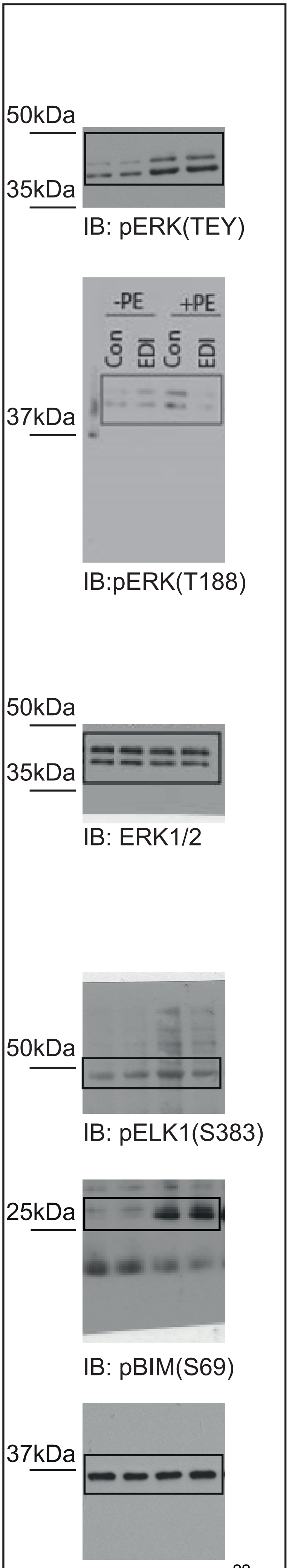

Figure 4H

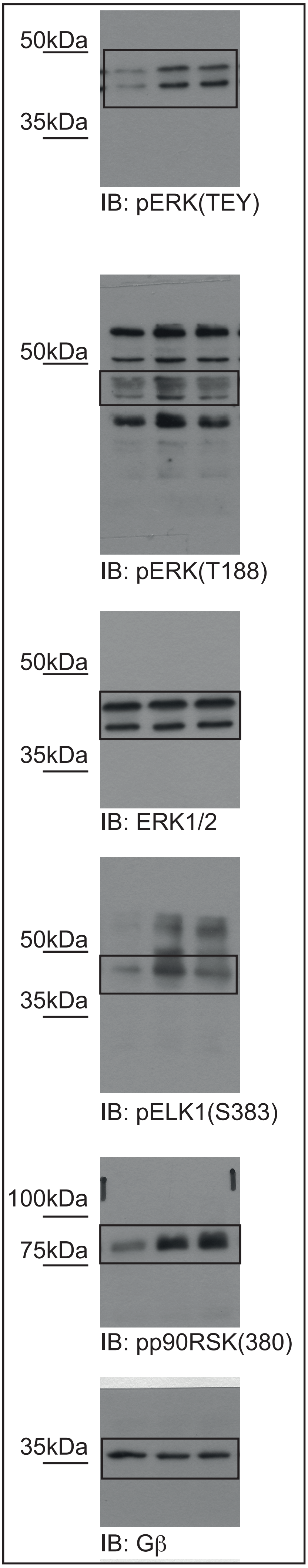

Supplementary Figure10 Uncropped Original Scans

Figure 5B

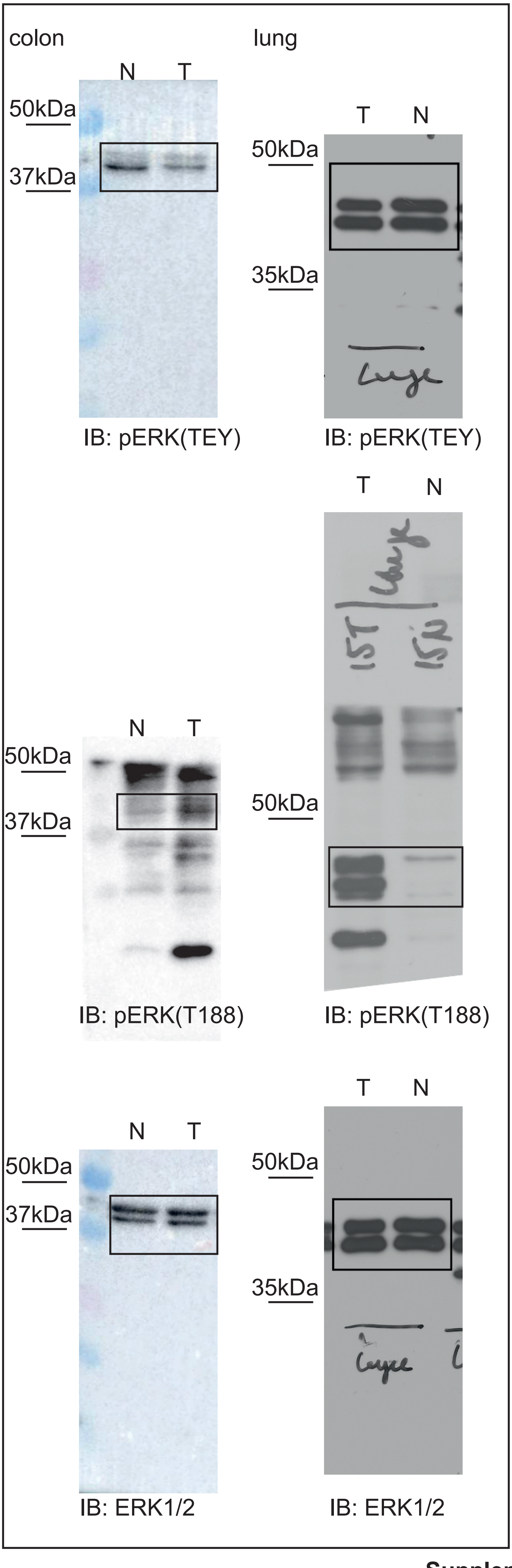

Figure 5D

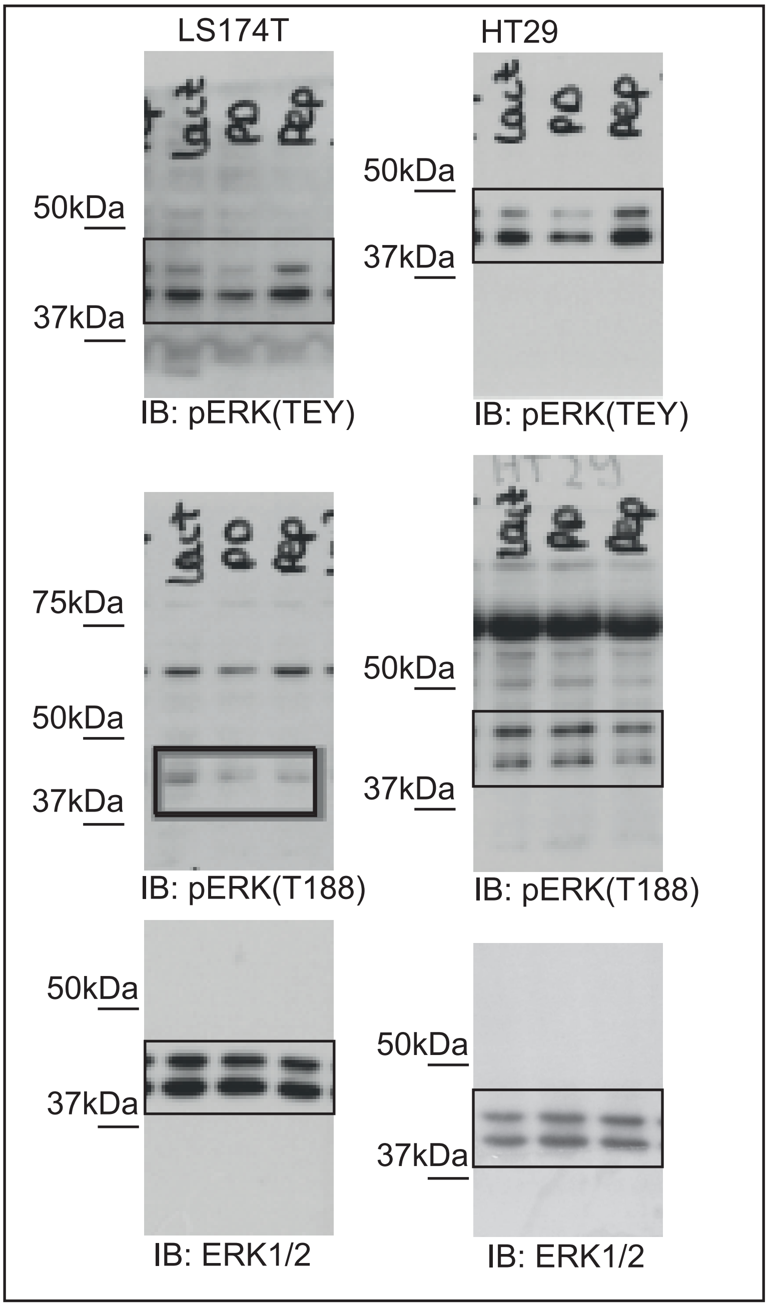

Figure 5E

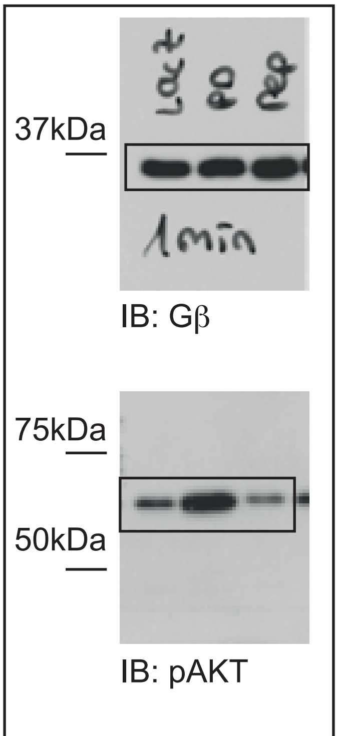

Supplementary Figure10 Uncropped Original Scans, continued

Figure 6A

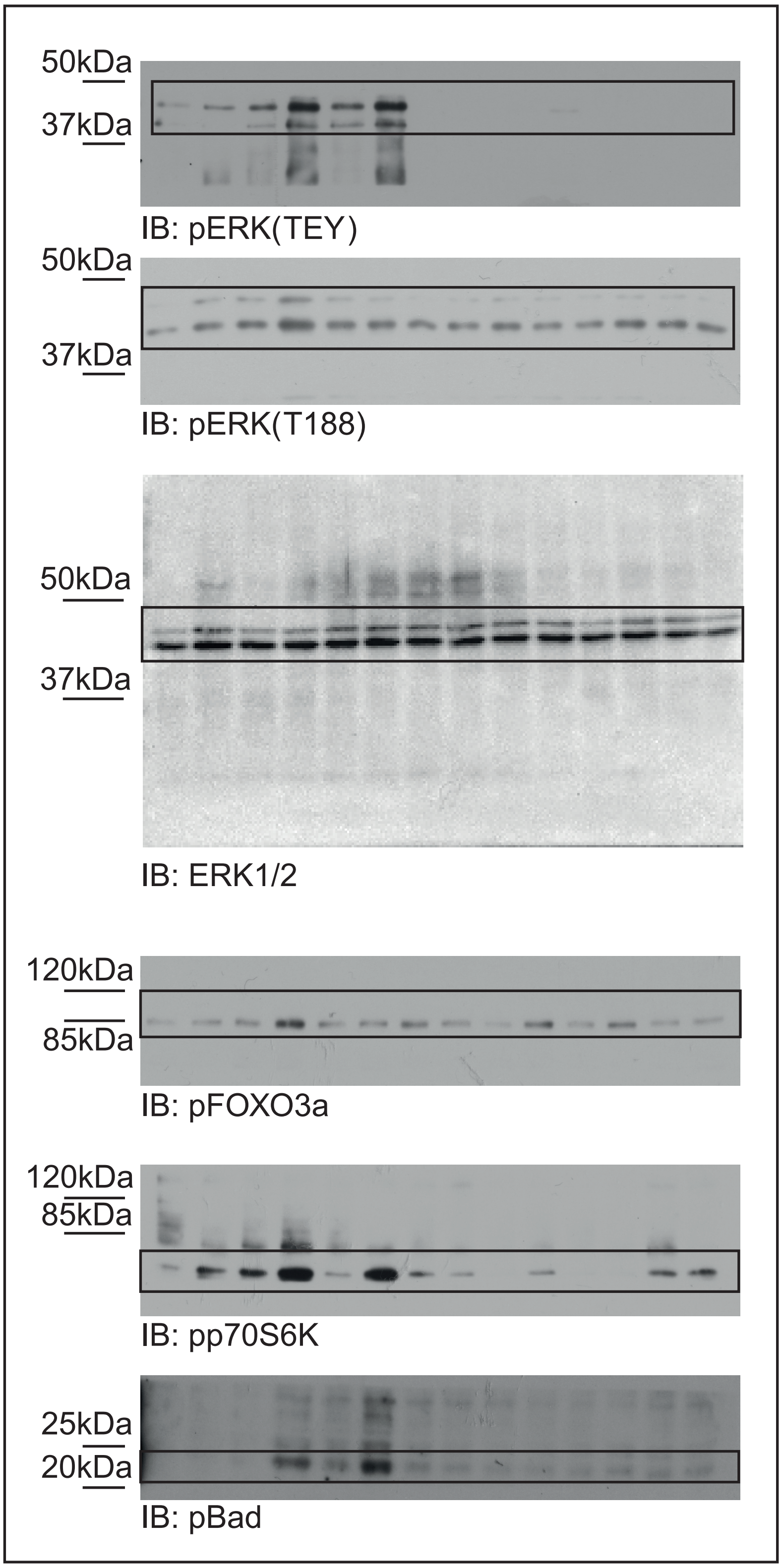

Supplementary Figure10 Uncropped Original Scans, continued

Supplementary Figure 1A

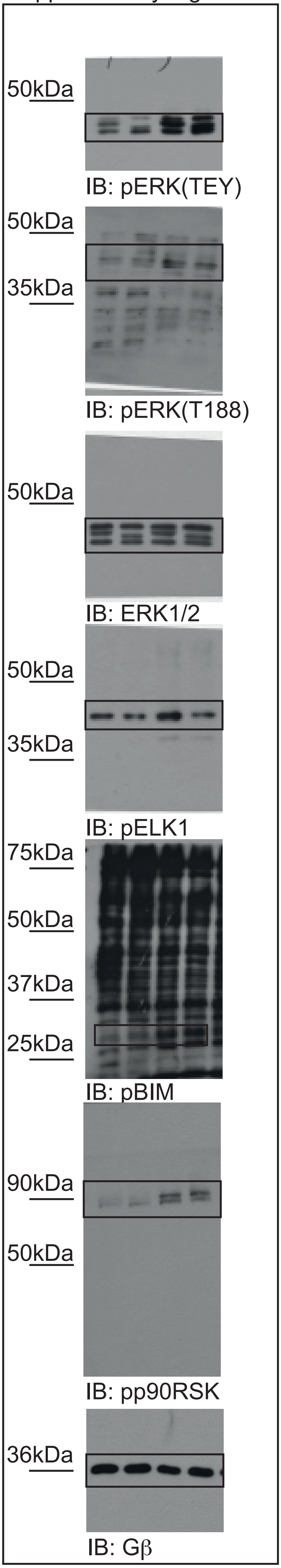

Supplementary Figure 1B

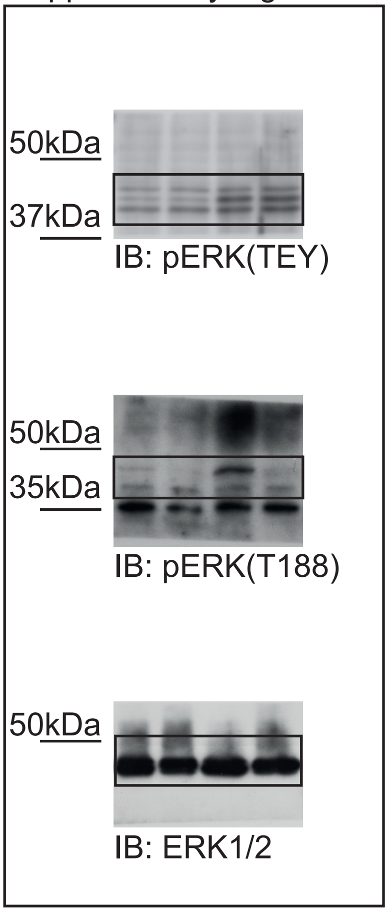

Supplementary Figure 1E

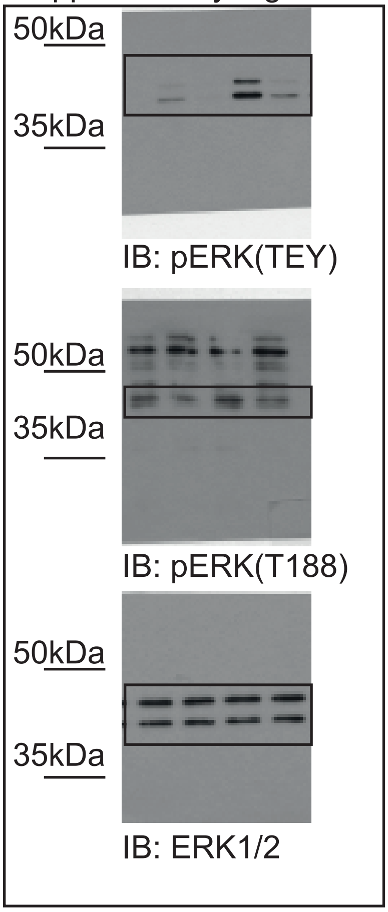

Supplementary Figure 2A

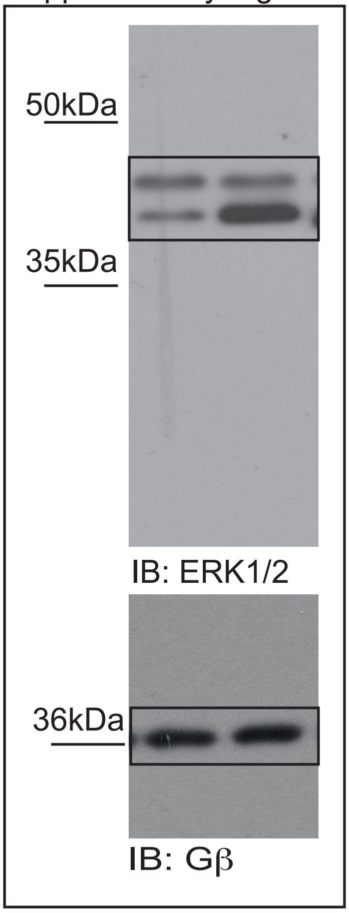

Supplementary Figure10 Uncropped Original Scans, continued

Supplementary Figure 5A

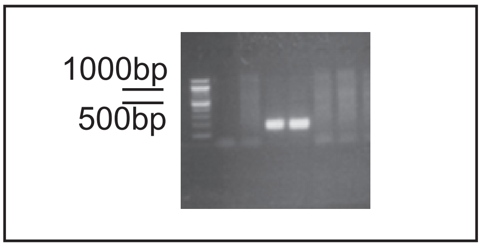

Supplementary Figure 5B

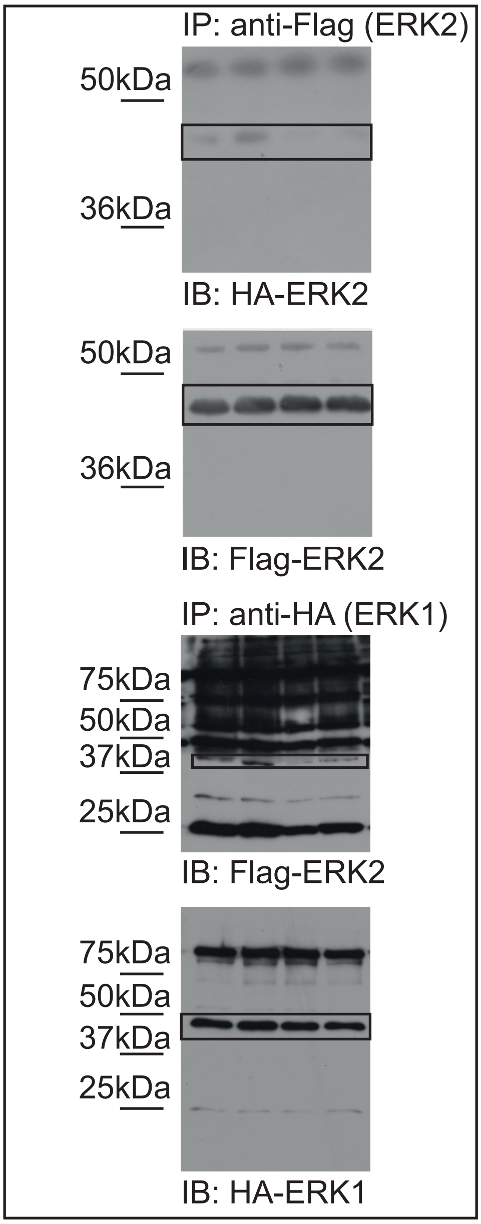

Supplementary Figure 5D

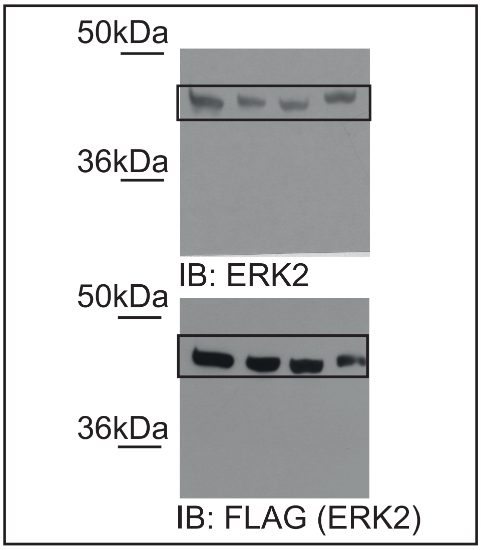

Supplementary Figure 5E

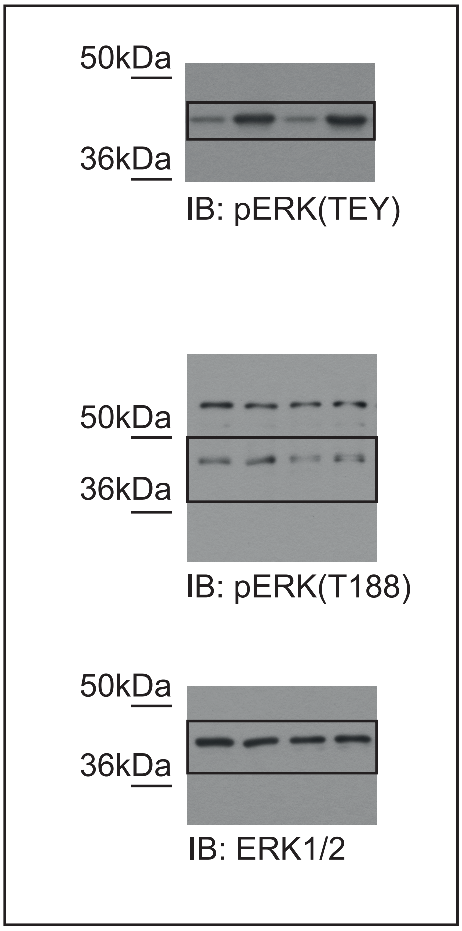

Supplementary Figure 5F

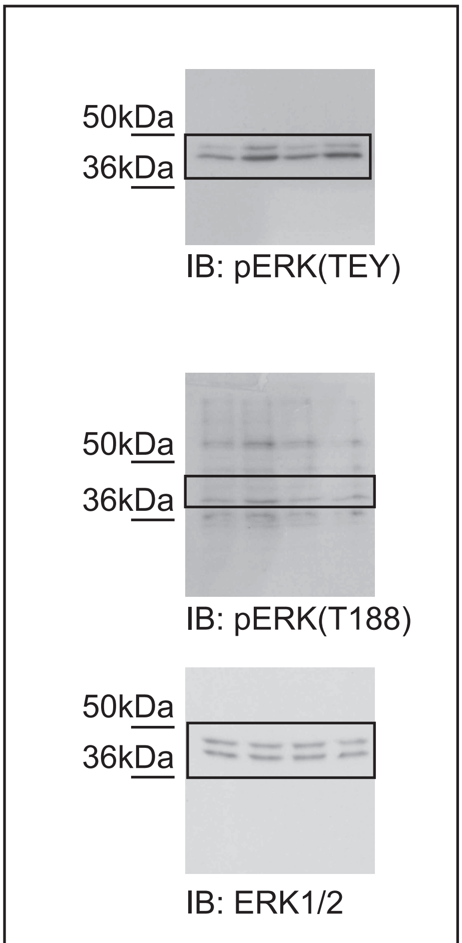

Supplementary Figure 6A

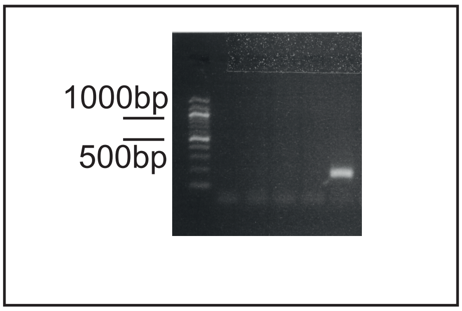

Supplementary Figure 6E

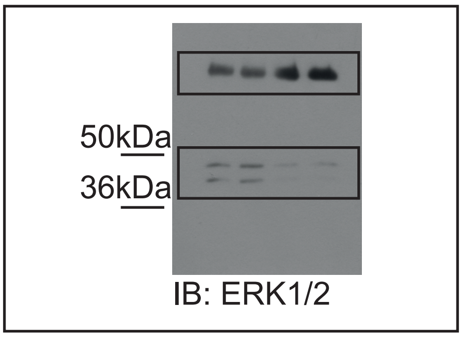

Supplementary Figure10 Uncropped Original Scans, continued

Supplementary Figure 8

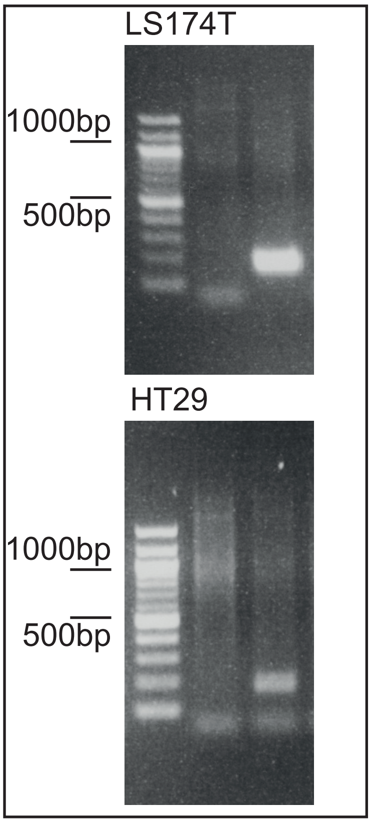

Supplementary Figure9B

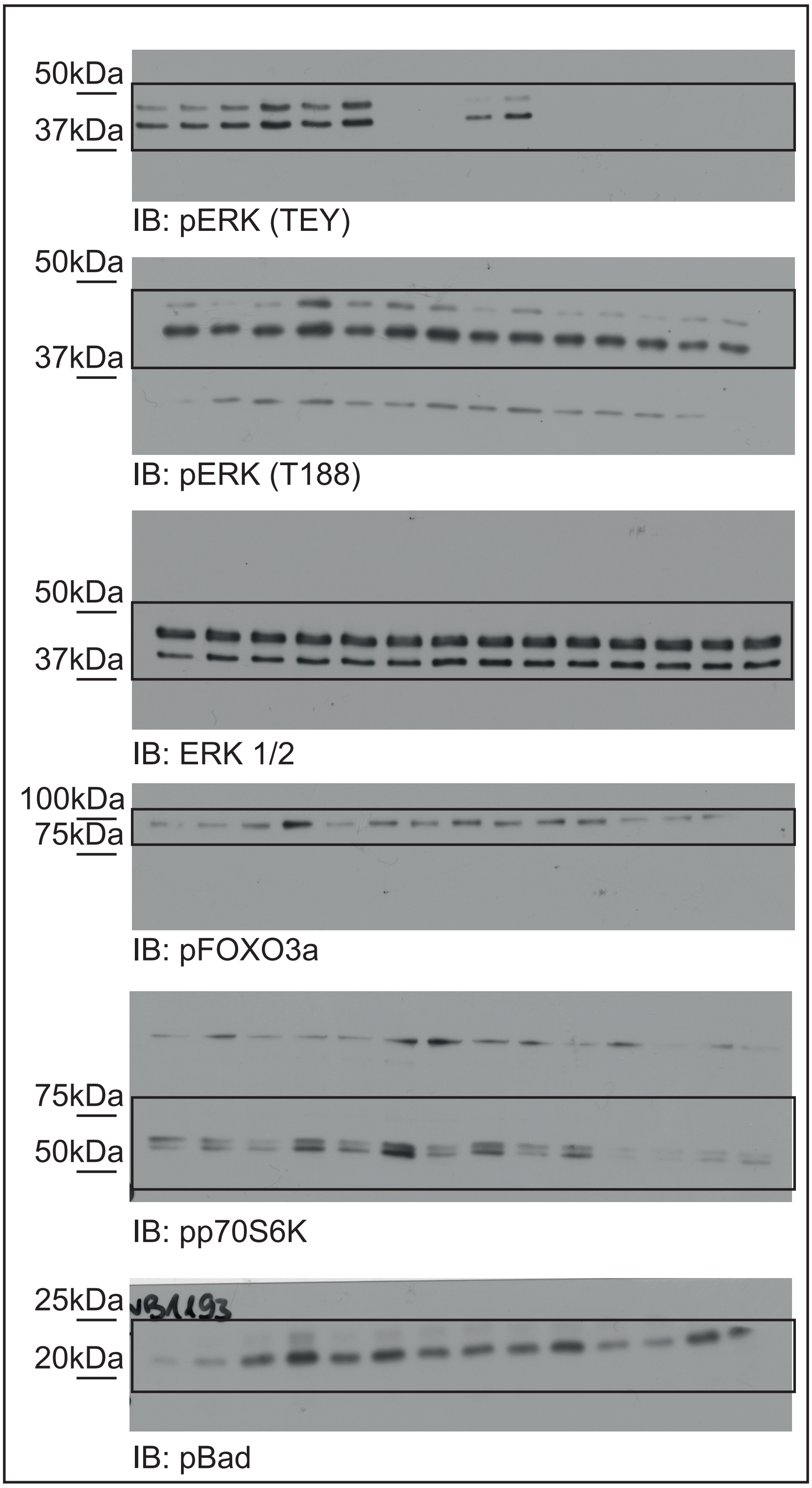

SupplementaryFigure 9C

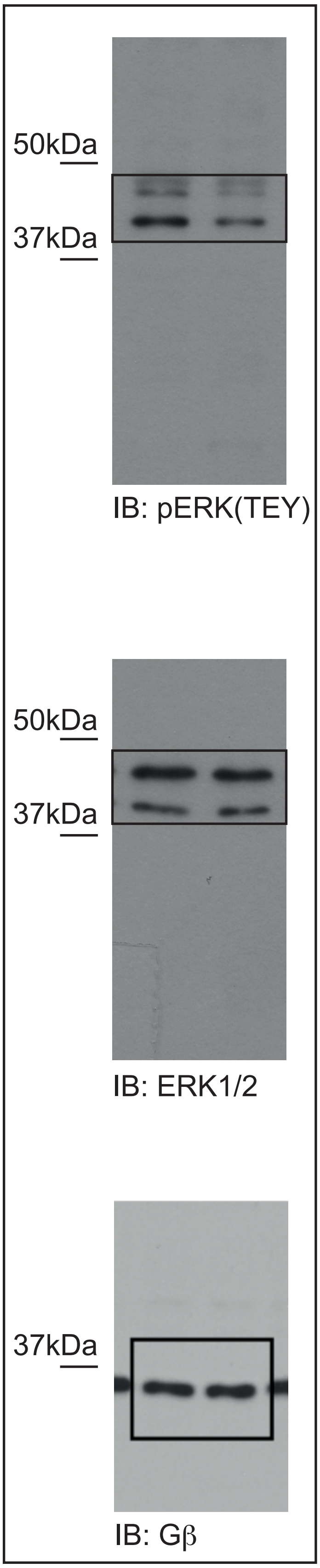

Supplementary Figure10 Uncropped Original Scans, continued

Supplementary Table 1
Characterization of cardiac function and histology of mice overexpressing monomeric ERK2 in response to TAC.

	Wt Con	ERK2-Δ4-tg Con	Wt TAC	ERK2-Δ4-tg TAC
**IVS, end-diastolic (mm)**	0.75 (±0.015)	0.78 (±0.014)	1.2 (±0.015)* $p < 0.0001$	1.0 (±0.014)*# $p < 0.0001$
**LVPW, end-diastolic (mm)**	0.77 (±0.020)	0.80 (±0.016)	1.1 (±0.023)* $p < 0.0001$	1.0 (±0.016)*# $p < 0.0001$
**LVID, end-diastolic (mm)**	3.3 (±0.097)	3.1 (±0.084)	3.2 (±0.074)	3.4 (±0.097)
**FS (%)**	38 (±1.4)	41 (±1.7)	36 (±2.0)	37 (±1.5)
**EF (%)**	69 (±1.8)	73 (±2.0)	66 (±2.8)	67 (1.8)
***n*-number**	12	10	15	10
**Heart rate (bpm)**	489 (±15)	466 (±8.9)	496 (±8.8)	473 (±9.8)
**Heart rate (dobutamine) (bpm)**	532 (±17)	504 (±10)	518 (±8.1)	490 (±8.6)
**LVP_{max} (mmHg)**	94 (±3.6)	92 (±3.3)	191 (±5.8)* $p < 0.0001$	179 (±6.1)* $p < 0.0001$
**LVP_{max} (dobutamine) (mmHg)**	100 (±3.5)	102 (±3.3)	201 (±6.3)* $p < 0.0001$	192 (±3.8)* $p < 0.0001$
**dp/dt_{min} (mmHg s^{-1})**	-6239 (±375)	-5751 (±353)	-9876 (±267)* $p < 0.0001$	-9538 (±433)* $p < 0.0001$
**dp/dt_{min} (dobutamine) (mmHg s^{-1})**	-6795 (±340)	-7110 (±366)	-10432 (±336)* $p < 0.0001$	-10398 (±377)* $p < 0.0001$
**dp/dt_{max} (mmHg s^{-1})**	6761 (±380)	6431 (±499)	8204 (±239)* $p = 0.0107$	8087 (±485)
**dp/dt_{max} (dobutamine) (mmHg s^{-1})**	8334 (±378)	8459 (±527)	9122 (±325)	9253 (±391)
***n*-number**	10	12	13	8

	Wt Con	ERK2-Δ4-tg Con	Wt TAC	ERK2-Δ4-tg TAC
**HW/TL (mg mm^{-1})**	5.7 (±0.12)	5.6 (±0.099)	10.8 (±0.25)* $p < 0.0001$	9.0 (±0.47)*# $p < 0.0001$
**HW/BW (mg g^{-1})**	4.2 (±0.10)	4.0 (±0.064)	7.1 (±0.16)* $p < 0.0001$	5.9 (±0.33)*# $p < 0.0001$
***n*-number**	13	10	20	10
**Interstitial fibrosis (A.U.)**	0.015 (±0.0042) *n*=8	0.014 (±0.0015) *n*=7	0.043 (±0.010)* $p = 0.0214$ *n*=10	0.016 (±0.0021)*# $p = 0.0173$ *n*=10
**Cardiomyocyte cross-sectional area (μm^2)**	201 (±11) *n*=8	192 (±11) *n*=9	308 (±24)*, $p = 0.0003$ *n*=10	227 (±10)*# $p = 0.0064$ *n*=9

Supplementary Table 1

Characterization of cardiac function and histology of mice overexpressing monomeric ERK2 in response to TAC. Characterization of wild-type (Wt) and ERK2$_{\Delta174\text{-}177}$ (ERK2-Δ4-tg) transgenic mice before and after TAC surgery (6 weeks). Shown are echocardiographic measurements of end-diastolic wall thicknesses (interventricular septal [IVS] and left ventricular posterior wall [LVPW]), left ventricular internal diameter (LVID), fractional shortening (FS), ejection fraction (EF) and heart rates; data derived from left ventricular cardiac catheterization with dobutamine dose response curves for dp/dt_{max} and dp/dt_{min} as determined for left ventricular pressure; heart weight-to-tibia length (HW/TL) and heart weight-to-body weight (HW/BW) ratios and data from analyses of fibrosis of Sirius Red-stained histological sections and data on cardiomyocyte cross-sectional area determined using H&E-stained histological sections of left ventricles. Values represent mean ± s.e.m.; *n*-numbers represent biologically independent animals; * *vs.* Wt control mice, # *vs.* Wt TAC mice. For statistical analysis ordinary one-way ANOVA was applied; for LVID, end-diastolic and EF Tukey test

and for all other parameters Bonferroni was applied as *post hoc* test. Source data are provided as a Source Data file.

Supplementary Table 2
Characterization of cardiac function of Wt and ERK2-Δ4 transgenic mice at the age of 9 months.

	Wt 9 months	ERK2-Δ4-tg 9 months
**IVS, end-diastolic (mm)**	0.87 (±0.016)	0.81 (±0.036)
**LVPW, end-diastolic (mm)**	0.96 (±0.017)	0.9 (±0.025)
**LVID, end-diastolic (mm)**	3.5 (±0.075)	3.5 (±0.083)
**FS (%)**	38 (±1.5)	35 (±2.3)
**EF (%)**	69 (±1.9)	65 (±3.2)
**Heart rate (bpm)**	539 (±8.5)	506 (±16)
**HW/TL (mg mm^{-1})**	7.6 (±0.23)	7.1 (±0.12)
**HW/BW (mg g^{-1})**	3.9 (±0.058)	3.7 (±0.069)
***n*-number**	9	9

Supplementary Table 2

Characterization of cardiac function of Wt and ERK2-Δ4 transgenic mice at the age of 9 months. Characterization of wild-type (Wt) and $ERK2_{\Delta174\text{-}177}$ (ERK2-Δ4) transgenic mice at the age of 9 months. Shown are echocardiographic measurements of end-diastolic wall thicknesses (interventricular septal [IVS] and left ventricular posterior wall [LVPW]), left ventricular internal diameter (LVID), fractional shortening (FS), ejection fraction (EF) and heart rates, heart weight-to-tibia length (HW/TL) and heart weight-to-body weight (HW/BW) ratios. Values represent mean ± s.e.m.. *n*-numbers represent biologically independent animals. For statistical analysis unpaired and two-sided Student's *t*-test was applied. Source data are provided as a Source Data file.

Supplementary Table 3
Characterization of Wt and ERK2-Δ4 transgenic mice after 3 weeks of voluntary running.

	Wt Con	ERK2-Δ4-tg Con	Wt RUN	ERK2-Δ4-tg RUN
**IVS, end-diastolic (mm)**	0.73 (±0.018)	0.73 (±0.015)	0.82 (±0.016)* p = 0.0087	0.85 (±0.018)* p = 0.0003
**LVPW, end-diastolic (mm)**	0.71 (±0.022)	0.71 (±0.016)	0.82 (±0.019)* p = 0.0046	0.84 (±0.014)* p = 0.0002
**LVID, end-diastolic (mm)**	3.3 (±0.098)	3.1 (±0.047)	3.3 (±0.071)	3.2 (±0.069)
**FS (%)**	39 (±1.5)	39 (±0.84)	38 (±2.0)	39 (±1.7)
**EF (%)**	63 (±4.7)	71 (±1.1)	62 (±5.4)	70 (±2.0)
**Heart rate (bpm)**	524 (±18)	545 (±11)	541 (±14)	520 (±15)
**Running distance**			371078 (±40582)	351894 (±45195)
***n*-number**	8	16	9	12
**HW/TL (mg mm^{-1})**	5.7 (±0.12)	5.6 (±0.099)	6.2 (±0.092)* p = 0.0164	6.2 (±0.12)* p = 0.004
**HW/BW (mg g^{-1})**	4.2 (±0.10)	4.0 (±0.064)	4.5 (±0.045)	4.6 (±0.17)
***n*-number**	13	10	9	13
**Interstitial fibrosis (A.U.)**	0.90 (±0.63) *n*=5	0.53 (±0.12) *n*=5	0.87 (±0.34) *n*=6	0.91 (±0.39) *n*=6
**Cardiomyocyte cross-sectional area (μm^2)**	290 (±22) *n*=7	287 (±10) *n*=7	386 (±10)* p = 0.001 *n*=6	368 (±12)* p = 0.004 *n*=8

Supplementary Table 3

Characterization of Wt and ERK2-Δ4 transgenic mice after 3 weeks of voluntary running. Characterization of wild-type (Wt) and ERK2$_{\Delta 174\text{-}177}$ (ERK2-Δ4-tg) transgenic mice before and after voluntary exercise in a running wheel (RUN) (3 weeks). Shown are echocardiographic measurements of end-diastolic wall thicknesses (interventricular septal [IVS] and left ventricular posterior wall [LVPW]), left ventricular internal diameter (LVID), fractional shortening (FS), ejection fraction (EF) and heart rates; heart weight-to-tibia length (HW/TL) and heart weight-to-body weight (HW/BW) ratios as well as and data from analyses of interstitial fibrosis of Sirius Red-stained histological sections and data on cardiomyocyte cross-sectional area determined using H&E-stained histological sections of left ventricles. Values represent mean ± s.e.m., *n*-numbers represent biologically independent animals; * *vs.* Wt control mice. For statistical analysis ordinary one-way ANOVA was applied, Bonferroni was applied as *post hoc* test. Source data are provided as a Source Data file.

Supplementary Table 4
Analysis of Wt mice 4 weeks after TAC surgery and AAV9-EDI treatment.

	**Wt<br>Con**	**Wt<br>+ AAV9-GFP<br>TAC**	**Wt<br>+ AAV-EDI**
**IVS, end-diastolic (mm)**	0,64 (±0.013)	1,0 (±0.024)*<br>$p < 0.0001$	0,80 (±0.019)*#<br>$p < 0.0001$
**LVPW, end-diastolic (mm)**	0,69 (±0.014)<br>$p < 0.0001$	0.99 (±0.030)*<br>$p < 0.0001$	0.82 (±0.0090)*#<br>$p < 0.0001$
**LVID, end-diastolic (mm)**	3,6 (±0.063)	3,9 (±0.060)*<br>$p = 0.011$	3,6 (±0.055)#<br>$p = 0.0213$
**FS (% of LVID)**	35 (±1.6)	22 (±1.2)*<br>$p < 0.0001$	36 (±1.5)#<br>$p < 0.0001$
**EF (%)**	64 (±2.1)	45 (±2.1)*<br>$p < 0.0001$	66 (±1.9)#<br>$p < 0.0001$
**Heart rate (bpm)**	509 (±12)	499 (±18)	531 (±17)
***n*-number**	19	11	14
**HW/TL (mg mm^{-1})**	5.2<br>(±0.17)	9.5<br>(±0.47)*<br>$p < 0.0001$	7.6<br>(±0.29)*#<br>* $p = 0.0004$<br># $p = 0.0013$
**HW/BW (mg g^{-1})**	4.3<br>(±0.12)	7.6<br>(±0.38)*<br>$p = 0.0054$	3.2<br>(±0.93)#<br>$p < 0.0001$
**LW/TL (mg mm^{-1})**	7.7<br>(±0.12)	9.1<br>(±0.23)*<br>$p = 0.0018$	7.9<br>(±0.30)#<br>$p = 0.0032$
***n*-number**	8	11	11
**Interstitial fibrosis (A.U.)**	0.38<br>(±0.065)<br>*n*=7	0.79<br>(±0.13)*<br>$p = 0.0467$<br>*n*=11	0.32<br>(±0.085)#<br>$p = 0.0076$<br>*n*=11
**Cardiomyocyte cross-sectional area (µm^2)**	175<br>(±5.2)<br>*n*=9	297<br>(±12)*<br>$p < 0.0001$<br>*n*=11	228<br>(±13)*#<br>* $p = 0.0102$<br># $p = 0.0004$<br>*n*=11

Supplementary Table 4

Analysis of Wt mice 4 weeks after TAC surgery and AAV9-EDI treatment. Basic echocardiographic analyses of untreated wild-type (Wt) and AAV9-EDI or AAV9-GFP treated mice after transverse aortic constriction (TAC; 4 weeks). Shown are echocardiographic measurements of end-diastolic wall thicknesses (interventricular septal [IVS] and left ventricular posterior wall [LVPW]), left ventricular internal diameter (LVID), fractional shortening (FS), ejection fraction (EF) and heart rates; heart weight to tibia length (HW/TL), heart weight-to-body weight (HW/BW) and lung weight-to-tibia length (LW/TL) ratios as well as data from analyses of interstitial fibrosis of Sirius Red-stained histological sections and data on cardiomyocyte cross-sectional area determined using H&E-stained histological sections of left ventricles. Values represent mean ± s.e.m., *n*-numbers represent biologically independent animals; * *vs.* Wt control mice, # *vs.* AAV9-GFP TAC mice. For statistical analysis ordinary one-way ANOVA was applied; for HW/TL, HW/BW, LW/TL, interstitial fibrosis, cardiomyocyte cross-sectional area Bonferroni, for FS Sidak, for IVS, end-diastolic, LVPW, end-diastolic LVID, end-diastolic, EF and heart rate Tukey test was applied as *post hoc* test. Source data are provided as a Source Data file.

Supplementary Table 5
Analysis of wild-type mice after AAV9-EDI treatment.

	Wt<br>Con	Wt<br>+ AAV9-GFP	Wt<br>+ AAV9-EDI
**IVS, end-diastolic (mm)**	0.65 (±0.016)	0.70 (±0.031)	0.69 (±0.012)
**LVPW, end-diastolic (mm)**	0.66 (±0.014)	0.65 (±0.0010)	0.66 (±0.012)
**LVID, end-diastolic (mm)**	3.5 (±0.058)	3.7 (±0.090)	3.4 (±0.059)
**FS (% of LVID)**	40 (±1.7)	40 (±3.5)	46 (±1.6)
**EF (%)**	71 (±2.2)	71 (±4.3)	78 (±1.7)
**Heart rate (bpm)**	621 (±15)	613 (±39)	634 (±26)
***n*-number**	18	6	8
**HW/TL (mg mm^{-1})**	5.8 (±0.095)	6.1 (±0.12)	6.1 (±0.29)
**HW/BW (mg mg^{-1})**	4.2 (±0.10)	4.3 (±0.067)	4.5 (±0.15)
**LW/TL (mg mm^{-1})**	8.0 (±0.35)	9.2 (±0.97)	8.9 (±1.0)
***n*-number**	11 (LW/TL12)	7	8
**Interstitial fibrosis (A.U.)**	0.09 (±0.014) *n*=6	0.11 (±0.023) *n*=6	0.09 (±0.019) *n*=8
**Cardiomyocyte cross-sectional area ($μm^2$)**	281 (±21) *n*=9	310 (±23) *n*=7	297 (±21) *n*=8

Supplementary Table 5

Analysis of wild-type mice after AAV9-EDI treatment. Echocardiographic analyses of wild-type (Wt) and Wt mice 4 weeks after AAV9-EDI or AAV9-GFP treatment. Shown are echocardiographic measurements of end-diastolic wall thicknesses (interventricular septal

[IVS] and left ventricular posterior wall [LVPW]), left ventricular internal diameter (LVID), fractional shortening (FS), ejection fraction (EF) and heart rates; heart weight-to-tibia length (HW/TL), heart weight-to-body weight (HW/BW) and lung weight to tibia length (LW/TL) ratios as well as data from analyses of interstitial fibrosis of Sirius Red-stained histological sections and data on cardiomyocyte cross-sectional area determined using H&E-stained histological sections of left ventricles. Values represent mean ± s.e.m.; *n*-numbers represent biologically independent animals. For statistical analysis ordinary one-way ANOVA and Bonferroni as *post hoc* test was applied. Tukey test was used as *post hoc* test. Source data are provided as a Source Data file.

Supplementary Table 6
Top 100 regulated genes in wild-type mice 4 weeks after TAC surgery and AAV9-EDI treatment. Table refers to heat map shown in Fig. 4G.

Gene symbol	Gene name	Fold change (*P*-value)		
		AAV9-GFP TAC *vs* Con	AAV9-EDI TAC *vs* Con	AAV9-EDI TAC *vs* AAV9-GFP TAC
Cilp	cartilage intermediate layer protein, nucleotide pyrophosphohydrolase	17.6 (1.37E-04)	3.37 (1.22E-01)	-5.21 (2.13E-03)
Ltbp2	latent transforming growth factor beta binding protein 2	9.91 (2.80E-03)	1.89 (7.39E-01)	-5.24 (1.42E-02)
Postn	periostin. osteoblast specific factor	8.35 (9.57E-05)	1.90 (2.66E-01)	-4.40 (9.31E-04)
S100a9	S100 calcium binding protein A9 (calgranulin B)	8.04 (5.03E-02)	4.96 (5.02E-01)	-1.62 (7.63E-01)
Serpina3n	serine (or cysteine) peptidase inhibitor. clade A. member 3N	5.95 (4.31E-02)	2.78 (6.57E-01)	-2.14 (4.40E-01)
Crlf1	cytokine receptor-like factor 1	5.70 (2.12E-03)	1.73 (6.60E-01)	-3.29 (1.38E-02)
S100a8	S100 calcium binding protein A8 (calgranulin A)	5.18 (1.13E-01)	3.32 (6.64E-01)	-1.56 (7.87E-01)
Col8a1	Collagen, type VIII, alpha 1	5.13 (1.60E-04)	1.52 (4.76E-01)	-3.37 (9.91E-04)
Thbs4	thrombospondin 4	5.06 (5.60E-03)	1.75 (7.03E-01)	-2.89 (3.76E-02)
Mfap4	microfibrillar-associated protein 4	4.78 (5.18E-04)	1.55 (5.71E-01)	-3.09 (2.74E-03)
Cdkn1a	cyclin-dependent kinase inhibitor 1A (P21)	4.46 (5.33E-03)	1.51 (7.79E-01)	-2.95 (2.36E-02)
Mybpc2	myosin binding protein C, fast-type	4.16 (3.41E-04)	2.73 (4.60E-02)	-1.52 (1.06E-01)
Itgbl1	Integrin, beta-like 1	4.02 (1.09E-03)	1.69 (4.65E-01)	-2.38 (1.26E-02)
Prg4	proteoglycan 4 (megakaryocyte stimulating factor, articular superficial zone protein)	3.84 (2.67E-02)	2.36 (5.12E-01)	-1.63 (4.70E-01)
Ccl8	chemokine (C-C motif) ligand 8	3.79 (3.58E-03)	1.99 (3.99E-01)	-1.91 (9.45E-02)
Mt2	metallothionein 2	3.72 (3.35E-03)	1.56 (6.68E-01)	-2.38 (2.56E-02)

Gene symbol	Gene name	Fold change (*P*-value)		
		AAV9-GFP TAC *vs* Con	AAV9-EDI TAC *vs* Con	AAV9-EDI TAC *vs* AAV9-GFP TAC
Nppa	natriuretic peptide type A	3.57 (1.37E-04)	2.02 (5.68E-02)	-1.77 (7.98E-03)
Nppb	natriuretic peptide type B	3.37 (5.11E-03)	1.65 (5.87E-01)	-2.04 (5.43E-02)
Svep1	Sushi, von Willebrand factor type A. EGF and pentraxin domain containing 1	3.33 (6.54E-04)	1.59 (3.86E-01)	-2.09 (9.13E-03)
Tnfrsf12a	tumor necrosis factor receptor superfamily. member 12a	3.01 (3.03E-03)	1.63 (4.75E-01)	-1.85 (4.48E-02)
Ddit4	DNA-damage-inducible transcript 4	2.72 (2.05E-02)	1.62 (6.62E-01)	-1.68 (2.12E-01)
Myh7	myosin. heavy polypeptide 7, cardiac muscle. beta	7.94 (4.39E-04)	5.8 (3.04E-02)	-1.37 (5.54E-01)
Cnksr1	connector enhancer of kinase suppressor of Ras 1	4.80 (1.08E-03)	3.30 (8.07E-02)	-1.45 (3.68E-01)
Myot	myotilin	4.27 (5.19E-04)	3.68 (3.04E-02)	-1.16 (7.64E-01)
Acta1	actin. alpha 1, skeletal muscle	4.26 (3.80E-05)	3.14 (3.75E-03)	-1.36 (6.72E-02)
Cpxm2	carboxypeptidase X 2 (M14 family)	4.12 (1.37E-04)	2.97 (1.83E-02)	-1.39 (1.27E-01)
Gck	glucokinase	3.73 (7.58E-04)	3.01 (4.89E-02)	-1.24 (5.53E-01)
Wisp2	WNT1 inducible signaling pathway protein 2	2.60 (4.10E-02)	1.89 (5.47E-01)	-1.38 (5.83E-01)
Lcn2	lipocalin 2	2.29 (4.32E-01)	2.37 (7.47E-01)	1.03 (9.87E-01)
Mmp3	matrix metallopeptidase 3	2.00 (3.07E-01)	1.92 (7.17E-01)	-1.04 (9.79E-01)
C3	complement component 3	1.78 (3.00E-01)	1.77 (6.87E-01)	-1.01 (9.93E-01)
Ift122	intraflagellar transport 122	1.71 (2.50E-01)	-1.97 (4.90E-01)	-3.38 (9.67E-03)
Top2a	topoisomerase (DNA) II alpha	1.82 (1.87E-01)	-1.62 (6.94E-01)	-2.95 (1.64E-02)
Comp	cartilage oligomeric matrix protein	6.88 (5.64E-03)	1.20 (9.12E-01)	-5.72 (8.24E-03)

Gene symbol	Gene name	Fold change (*P*-value)		
		AAV9-GFP TAC *vs* Con	AAV9-EDI TAC *vs* Con	AAV9-EDI TAC *vs* AAV9-GFP TAC
Thbs1	thrombospondin 1	5.64 (2.24E-04)	1.00 (9.99E-01)	-5.63 (6.09E-04)
Timp1	tissue inhibitor of metalloproteinase 1	5.92 (8.15E-04)	1.46 (7.56E-01)	-4.05 (3.21E-03)
Gdf15	growth differentiation factor 15	5.62 (4.79E-03)	1.27 (8.79E-01)	-4.43 (9.57E-03)
Ctgf	connective tissue growth factor	5.01 (1.37E-04)	1.39 (5.81E-01)	-3.61 (8.44E-04)
Col5a2	collagen. type V, alpha 2	4.73 (3.60E-04)	1.20 (8.49E-01)	-3.94 (9.82E-04)
Fmod	fibromodulin	4.49 (2.80E-03)	1.18 (8.88E-01)	-3.79 (5.12E-03)
Mfap5	microfibrillar associated protein 5	4.36 (2.62E-04)	1.50 (5.12E-01)	-2.92 (1.74E-03)
Egr2	early growth response 2	3.61 (1.58E-02)	-1.06 (9.70E-01)	-3.83 (1.11E-02)
Apold1	apolipoprotein L domain containing 1	3.92 (3.80E-05)	1.17 (7.47E-01)	-3.36 (8.51E-05)
Col3a1	collagen. type III, alpha 1	3.21 (3.75E-03)	-1.21 (8.59E-01)	-3.9 (1.74E-03)
Spp1	secreted phosphoprotein 1	3.56 (1.56E-02)	1.07 (9.67E-01)	-3.33 (1.87E-02)
Col8a2	collagen. type VIII, alpha 2	3.54 (6.09E-03)	1.12 (9.18E-01)	-3.15 (9.29E-03)
Col1a2	collagen. type I, alpha 2	3.46 (4.84E-04)	1.05 (9.54E-01)	-3.30 (9.59E-04)
1500015O10Rik	RIKEN cDNA 1500015O10 gene	3.43 (3.72E-03)	1.12 (9.09E-01)	-3.07 (5.90E-03)
Col1a1	collagen. type I, alpha 1	3.05 (2.51E-03)	-1.11 (9.06E-01)	-3.38 (1.90E-03)
Meox1	mesenchyme homeobox 1	3.01 (2.61E-03)	-1.12 (8.92E-01)	-3.38 (1.77E-03)
Pamr1	peptidase domain containing associated with muscle regeneration 1	3.57 (1.19E-02)	1.49 (7.81E-01)	-2.39 (5.63E-02)
Frzb	frizzled-related protein	3.47 (7.88E-04)	1.31 (7.50E-01)	-2.65 (3.21E-03)

Gene symbol	Gene name	Fold change (*P*-value)		
		AAV9-GFP TAC *vs* Con	AAV9-EDI TAC *vs* Con	AAV9-EDI TAC *vs* AAV9-GFP TAC
Fbn1	fibrillin 1	3.19 (6.47E-04)	1.00 (9.98E-01)	-3.18 (9.82E-04)
Fstl1	follistatin-like 1	3.14 (3.22E-04)	1.13 (8.61E-01)	-2.79 (9.51E-04)
Rcan1	regulator of calcineurin 1	3.02 (4.64E-04)	1.05 (9.41E-01)	-2.88 (9.82E-04)
Loxl2	lysyl oxidase-like 2	2.72 (3.63E-03)	-1.11 (9.01E-01)	-3.02 (2.27E-03)
Emp1	epithelial membrane protein 1	2.99 (1.60E-04)	1.12 (8.42E-01)	-2.68 (6.72E-04)
Bgn	biglycan	3.10 (3.44E-04)	1.33 (5.90E-01)	-2.32 (2.00E-03)
Rbp1	retinol binding protein 1, cellular	3.00 (4.76E-03)	1.18 (8.68E-01)	-2.54 (1.01E-02)
Sfrp2	secreted frizzled-related protein 2	3.01 (1.82E-02)	1.24 (8.63E-01)	-2.41 (4.15E-02)
Nmrk2	nicotinamide riboside kinase 2	3.04 (1.21E-01)	1.33 (8.85E-01)	-2.28 (2.57E-01)
Vcan	versican	2.86 (5.19E-04)	1.08 (9.04E-01)	-2.66 (1.10E-03)
Cthrc1	collagen triple helix repeat containing 1	2.92 (9.51E-03)	1.17 (8.82E-01)	-2.48 (1.87E-02)
Aldh 1a2	aldehyde dehydrogenase family 1, subfamily A2	2.94 (7.48E-03)	1.22 (8.60E-01)	-2.40 (1.94E-02)
Cebpd	CCAAT/enhancer binding protein (C/EBP), delta	2.97 (1.68E-03)	1.48 (5.61E-01)	-2.00 (1.86E-02)
Lox	lysyl oxidase	2.88 (4.23E-03)	1.23 (8.32E-01)	-2.34 (1.18E-02)
Serpine1	serine (or cysteine) peptidase inhibitor, clade E, member 1	2.31 (2.67E-02)	-1.21 (8.62E-01)	-2.79 (9.48E-03)
Col 15a1	collagen. type XV, alpha 1	2.05 (2.00E-02)	-1.39 (6.88E-01)	-2.85 (2.78E-03)
Serpinf1	serine (or cysteine) peptidase inhibitor. clade F. member 1	2.82 (1.70E-03)	1.30 (7.36E-01)	-2.16 (8.31E-03)
Gm 30873	predicted gene, 30873	2.72 (8.10E-03)	1.37 (7.52E-01)	-1.98 (4.32E-02)

Gene symbol	Gene name	Fold change (*P*-value)		
		AAV9-GFP TAC *vs* Con	AAV9-EDI TAC *vs* Con	AAV9-EDI TAC *vs* AAV9-GFP TAC
Tgfb2	transforming growth factor. beta 2	2.58 (8.60E-03)	1.08 (9.38E-01)	-2.39 (1.19E-02)
Ms 4a6d	membrane-spanning 4-domains. subfamily A, member 6D	2.68 (8.81E-03)	1.30 (8.06E-01)	-2.07 (3.53E-02)
Arntl	aryl hydrocarbon receptor nuclear translocator-like	2.19 (5.24E-02)	-1.19 (8.82E-01)	-2.6 (1.79E-02)
Gem	GTP binding protein (gene overexpressed in skeletal muscle)	2.15 (7.46E-02)	1.00 (9.98E-01)	-2.14 (6.61E-02)
Sprr1a	small proline-rich protein 1A	1.92 (2.26E-01)	1.10 (9.50E-01)	-1.74 (2.97E-01)
Nlrc5	NLR family, CARD domain containing 5	1.69 (3.66E-01)	-1.13 (9.40E-01)	-1.91 (2.23E-01)
Angpt1	angiopoietin 1	-5.3 (1.37E-04)	-2.17 (8.41E-02)	2.44 (3.42E-03)
Gm 19277	predicted gene, 19277	-3.85 (1.41E-03)	-1.86 (3.58E-01)	2.07 (3.25E-02)
Cngb3	cyclic nucleotide gated channel beta 3	-3.21 (2.52E-03)	-1.97 (2.79E-01)	1.63 (1.23E-01)
Snord 14e	small nucleolar RNA, C/D box 14E	-2.92 (2.20E-02)	-1.83 (5.75E-01)	1.60 (3.16E-01)
Gpr22	G protein-coupled receptor 22	-4.21 (1.17E-03)	1.02 (9.88E-01)	4.29 (1.45E-03)
Ces1d	carboxylesterase 1D	-3.85 (4.80E-04)	-1.49 (5.38E-01)	2.58 (3.49E-03)
Pfkfb1	6-phosphofructo-2-kinase/fructose-2.6-biphosphatase 1	-3.48 (-.32E-04)	-1.23 (6.94E-01)	2.83 (6.17E-04)
Il15	interleukin 15	-3.12 (3.60E-04)	-1.35 (5.63E-01)	2.31 (2.35E-03)
Inmt	indolethylamine N-methyltransferase	-2.11 (3.58E-01)	1.49 (8.55E-01)	3.15 (1.14E-01)
Mylk4	myosin light chain kinase family. member 4	-2.78 (8.92E-04)	-1.06 (9.39E-01)	2.63 (1.74E-03)
Acot1	acyl-CoA thioesterase 1	-2.72 (9.53E-03)	-1.40 (7.47E-01)	1.95 (5.64E-02)
Dbp	D site albumin promoter binding protein	-2.48 (5.49E-02)	-1.02 (9.93E-01)	2.44 (5.12E-02)

Gene symbol	Gene name	Fold change (*P*-value)		
		AAV9-GFP TAC *vs* Con	AAV9-EDI TAC *vs* Con	AAV9-EDI TAC *vs* AAV9-GFP TAC
Ppip 5k2	diphosphoinositol pentakisphosphate kinase 2	-2.45 (8.55E-03)	-1.17 (8.62E-01)	2.10 (2.00E-02)
Tmem 267	transmembrane protein 267	-1.68 (4.56E-01)	-1.15 (9.37E-01)	1.46 (6.56E-01)
Egr1	early growth response 1	1.22 (8.28E-01)	-2.30 (5.78E-01)	-2.80 (9.52E-02)
Fos	FBJ osteosarcoma oncogene	-1.15 (8.40E-01)	-2.61 (2.95E-01)	-2.28 (7.39E-02)
Iigp1	interferon inducible GTPase 1	-1.31 (7.52E-01)	-1.51 (8.27E-01)	-1.15 (9.15E-01)
Irgm2	immunity-related GTPase family M member 2	1.42 (6.79E-01)	-1.26 (8.99E-01)	-1.79 (4.38E-01)
BC 023105	cDNA sequence BC023105	1.21 (9.26E-01)	-1.38 (9.28E-01)	-1.68 (8.05E-01)
Igtp	interferon gamma induced GTPase	1.20 (8.54E-01)	-1.26 (8.97E-01)	-1.51 (6.39E-01)
Saa3	serum amyloid A 3	1.34 (6.21E-01)	1.13 (9.22E-01)	-1.18 (8.48E-01)
Cyp 26b1	cytochrome P450. family 26. subfamily b. polypeptide 1	-1.1 (9.29E-01)	1.13 (9.47E-01)	1.24 (8.53E-01)
C1s1	complement component 1. s subcomponent 1	1.10 (9.37E-01)	1.03 (9.93E-01)	-1.07 (9.71E-01)

Supplementary Table 6

Top 100 regulated genes in wild-type mice 4 weeks after TAC surgery and AAV9-EDI treatment. The table refers to the heat map shown in Fig. 4G; gene array of untreated wild type (Wt; n=3) and AAV9-EDI (n=4) or AAV9-GFP (n=4) treated mice after transverse aortic constriction (TAC; 4 weeks). The table indicates the gene symbol, gene name and fold-changes and *p*-values; *n*-numbers represent samples of individual mice. For statistical analysis ANOVA with Tukey *post hoc* test was applied. Source data are provided under the following link: https://www.ebi.ac.uk/arrayexpress/experiments/E-MTAB-8110.

Supplementary Table 7
Regulated genes associated with cardiac hypertrophy in wild-type mice 4 weeks after TAC surgery and AAV9-EDI treatment. Table refers to the heat map shown in Supplementary Figure 7B.

Gene symbol	Gene name	Fold change (*P*-value)		
		AAV9-GFP TAC *vs* Con	AAV9-EDI TAC *vs* Con	AAV9-EDI TAC *vs* AAV9-GFP TAC
Postn	periostin, osteoblast specific factor	8.34 (1.70E-05)	1.89 (1.20E-01)	-4.40 (4.37E-04)
Myh7	myosin, heavy polypeptide 7, cardiac muscle, beta	6.20 (7.95E-04)	4.53 (3.11E-02)	-1.37 (6.30E-01)
Ctgf	connective tissue growth factor	4.84 (3.12E-05)	1.34 (4.91E-01)	-3.61 (4.21E-04)
S100a8	S100 calcium binding protein A8 (calgranulin A)	4.61 (8.49E-02)	2.95 (5.68E-01)	-1.56 (7.68E-01)
Cdkn1a	cyclin-dependent kinase inhibitor 1A (P21)	4.61 (1.54E-03)	1.56 (6.35E-01)	-2.95 (1.76E-02)
Mybpc2	myosin binding protein C, fast-type	4.52 (7.20E-05)	2.97 (1.13E-02)	-1.52 (1.08E-01)
Nppb	natriuretic peptide type B	3.80 (1.55E-03)	1.86 (3.25E-01)	-2.04 (5.75E-02)
Nppa	natriuretic peptide type A	3.74 (3.31E-05)	2.12 (1.26E-02)	-1.77 (6.44E-03)
Prg4	proteoglycan 4 (megakaryocyte stimulating factor, articular superficial zone protein)	3.41 (2.08E-02)	2.10 (4.49E-01)	-1.63 (4.52E-01)
Rcan1	regulator of calcineurin 1	3.15 (1.02E-04)	1.09 (8.56E-01)	-2.88 (5.38E-04)
Pi16	peptidase inhibitor 16	2.75 (1.13E-03)	1.39 (5.37E-01)	-1.99 (1.69E-02)
Fstl3	follistatin-like 3	2.68 (1.71E-04)	1.74 (6.37E-02)	-1.54 (3.55E-02)
Fn1	fibronectin 1	2.65 (2.29E-04)	1.06 (9.05E-01)	-2.49 (9.27E-04)
Nox4	NADPH oxidase 4	2.62 (4.87E-03)	1.49 (5.18E-01)	-1.76 (9.01E-02)
Tgfb2	transforming growth factor, beta 2	2.48 (4.20E-03)	1.04 (9.60E-01)	-2.39 (8.45E-03)
Fhl1	four and a half LIM domains 1	2.48 (1.32E-03)	1.48 (3.56E-01)	-1.68 (4.29E-02)
Errfi1	ERBB receptor feedback inhibitor 1	2.41 (7.04E-03)	1.19 (8.21E-01)	-2.03 (3.12E-02)

Gene symbol	Gene name	Fold change (*P*-value)		
		AAV9-GFP TAC *vs* Con	AAV9-EDI TAC *vs* Con	AAV9-EDI TAC *vs* AAV9-GFP TAC
Fgl2	fibrinogen-like protein 2	2.20 (8.58E-03)	1.05 (9.47E-01)	-2.10 (1.81E-02)
Eln	elastin	2.20 (1.67E-04)	-1.00 (9.90E-01)	-2.21 (5.38E-04)
Il6	interleukin 6	2.16 (1.56E-03)	1.04 (9.47E-01)	-2.09 (3.70E-03)
Cdk1	cyclin-dependent kinase 1	2.13 (1.33E-02)	1.08 (9.16E-01)	-1.97 (3.09E-02)
Mmp2	matrix metallopeptidase 2	2.06 (1.45E-03)	1.06 (9.08E-01)	-1.94 (4.52E-03)
Apod	apolipoprotein D	2.04 (4.78E-04)	1.54 (8.51E-02)	-1.32 (1.04E-01)
Hbegf	heparin-binding EGF-like growth factor	2.02 (9.19E-04)	1.19 (6.72E-01)	-1.69 (9.32E-03)
Ace	angiotensin I converting enzyme (peptidyl-dipeptidase A) 1	1.94 (4.71E-04)	1.2 (5.47E-01)	-1.62 (6.00E-03)
Edn3	endothelin 3	1.93 (1.15E-04)	1.63 (1.52E-02)	-1.19 (1.92E-01)
Tlr4	toll-like receptor 4	1.90 (1.81E-02)	1.23 (7.38E-01)	-1.54 (1.28E-01)
Bcl2	B cell leukemia/lymphoma 2	1.90 (1.10E-03)	1.06 (8.96E-01)	-1.80 (3.71E-03)
F2r	coagulation factor II (thrombin) receptor	1.88 (1.89E-03)	-1.08 (8.49E-01)	-2.03 (1.82E-03)
Igf1	insulin-like growth factor 1	1.87 (5.32E-04)	1.02 (9.64E-01)	-1.84 (1.51E-03)
Abat	4-aminobutyrate aminotransferase	1.86 (3.07E-03)	1.89 (2.81E-02)	1.02 (9.75E-01)
Col14a 1	collagen, type XIV, alpha 1	1.82 (2.33E-03)	1.00 (9.93E-01)	-1.81 (4.21E-03)
Nr4a1	nuclear receptor subfamily 4, group A, member 1	1.82 (2.04E-01)	-1.07 (9.51E-01)	-1.95 (1.61E-01)
Plat	plasminogen activator, tissue	1.79 (1.06E-03)	1.06 (8.78E-01)	-1.69 (4.35E-03)
Lbp	lipopolysaccharide binding protein	1.76 (1.73E-03)	1.34 (2.70E-01)	-1.32 (9.55E-02)
Shisa3	shisa family member 3	1.74 (4.06E-02)	1.08 (9.12E-01)	-1.61 (9.50E-02)

Gene symbol	Gene name	Fold change (*P*-value)		
		AAV9-GFP TAC *vs* Con	AAV9-EDI TAC *vs* Con	AAV9-EDI TAC *vs* AAV9-GFP TAC
Tnfaip6	tumor necrosis factor alpha induced protein 6	1.66 (1.79E-03)	1.04 (9.14E-01)	-1.59 (5.31E-03)
Foxc2	forkhead box C2	1.58 (1.19E-02)	-1.01 (9.90E-01)	-1.59 (1.71E-02)
Gpx3	glutathione peroxidase 3	1.58 (6.52E-02)	1.14 (8.27E-01)	-1.39 (2.43E-01)
Kif5b	kinesin family member 5B	1.56 (1.40E-03)	1.12 (6.74E-01)	-1.39 (1.38E-02)
Smad1	SMAD family member 1	1.53 (3.08E-03)	-1.11 (7.34E-01)	-1.70 (1.66E-03)
Clec4d	C-type lectin domain family 4, member d	1.51 (5.76E-02)	1.16 (7.79E-01)	-1.31 (2.83E-01)
Kcnk2	potassium channel, subfamily K, member 2	1.43 (3.40E-02)	-1.13 (7.70E-01)	-1.61 (1.22E-02)
Trf	transferrin	1.43 (5.58E-02)	-1.13 (7.90E-01)	-1.61 (2.01E-02)
Fetub	fetuin beta	1.39 (1.13E-01)	1.32 (4.92E-01)	-1.05 (9.12E-01)
Wisp1	WNT1 inducible signaling pathway protein 1	1.36 (2.80E-01)	-1.18 (7.93E-01)	-1.61 (9.79E-02)
Grk5	G protein-coupled receptor kinase 5	1.34 (4.23E-01)	-1.16 (8.55E-01)	-1.55 (2.30E-01)
Tmem 100	transmembrane protein 100	-1.40 (1.83E-01)	-1.17 (7.87E-01)	1.19 (6.24E-01)
Parp1	poly (ADP-ribose) polymerase family, member 1	-1.55 (1.00E-03)	-1.19 (3.94E-01)	1.30 (2.89E-02)
Adrb1	adrenergic receptor, beta 1	-1.59 (2.27E-02)	-1.40 (3.19E-01)	1.13 (6.87E-01)
Tbx5	T-box 5	-1.63 (1.33E-03)	-1.04 (9.03E-01)	1.57 (3.96E-03)
Myocd	myocardin	-1.64 (7.28E-05)	-1.25 (8.60E-02)	1.31 (5.79E-03)
Corin	corin	-1.64 (1.67E-03)	-1.03 (9.32E-01)	1.59 (4.52E-03)
Tcap	titin-cap	-1.66 (4.92E-03)	-1.04 (9.17E-01)	1.59 (1.28E-02)
Amy1	amylase 1, salivary	-2.05 (2.67E-03)	1.11 (8.40E-01)	2.28 (2.12E-03)

Gene symbol	Gene name	Fold change (*P*-value)		
		AAV9-GFP TAC *vs* Con	AAV9-EDI TAC *vs* Con	AAV9-EDI TAC *vs* AAV9-GFP TAC
Rgs2	regulator of G-protein signaling 2	-2.21 (1.61E-03)	-1.77 (8.71E-02)	1.25 (3.97E-01)
Kcnd2	potassium voltage-gated channel, Shal-related family, member 2	-2.23 (4.72E-04)	-1.49 (1.74E-01)	1.50 (3.97E-02)
Penk	preproenkephalin	-2.30 (6.02E-04)	-1.47 (2.26E-01)	1.57 (3.52E-02)
Mylk4	myosin light chain kinase family, member 4	-2.73 (2.69E-04)	-1.04 (9.47E-01)	2.63 (9.70E-04)
Phkg1	phosphorylase kinase gamma 1	-2.84 (4.16E-05)	-1.75 (2.89E-02)	1.62 (7.96E-03)
Ces1d	carboxylesterase 1D	-3.79 (1.25E-04)	-1.47 (3.88E-01)	2.58 (2.17E-03)

Supplementary Table 7

Regulated genes associated with cardiac hypertrophy in wild-type mice 4 weeks after TAC surgery and AAV9-EDI treatment. The table refers to the heat map shown in Supplementary Figure 7B; genes reported by Gene Ontology as associated with cardiac hypertrophy and regulated in untreated wild-type (Wt, n=3) versus AAV9-EDI (n=4) or AAV9-GFP (n=4) treated mice after transverse aortic constriction (TAC; 4 weeks). The table indicates the gene symbol, gene name and fold-changes and *p*-values; *n*-numbers represent samples of individual mice. For statistical analysis ANOVA with Tukey *post hoc* test was applied. Source data are provided under the following link: https://www.ebi.ac.uk/arrayexpress/experiments/E-MTAB-8110.

Supplementary Table 8
Regulated genes associated with extracellular matrix in wild-type mice 4 weeks after TAC surgery and AAV9-EDI treatment. The table refers to the heat map shown in Supplementary Figure 7B.

Gene symbol	Gene name	Fold change (*P*-value)		
		AAV9-GFP TAC *vs* Con	AAV9-EDI TAC *vs* Con	AAV9-EDI TAC *vs* AAV9-GFP TAC
Postn	periostin, osteoblast specific factor	8.34 (1.70E-05)	1.89 (1.20E-01)	-4.40 (4.37E-04)
Mfap4	microfibrillar-associated protein 4	4.76 (1.16E-04)	1.54 (4.00E-01)	-3.09 (1.61E-03)
Mfap5	microfibrillar associated protein 5	4.59 (5.25E-05)	1.57 (2.63E-01)	-2.92 (1.06E-03)
Fstl3	follistatin-like 3	2.68 (1.71E-04)	1.74 (6.37E-02)	-1.54 (3.55E-02)
Fn1	fibronectin 1	2.65 (2.29E-04)	1.06 (9.05E-01)	-2.49 (9.27E-04)
Nox4	NADPH oxidase 4	2.62 (4.87E-03)	1.49 (5.18E-01)	-1.76 (9.01E-02)
Serpine 1	serine (or cysteine) peptidase inhibitor, clade E, member 1	2.28 (1.23E-02)	-1.22 (8.02E-01)	-2.79 (6.36E-03)
Eln	elastin	2.20 (1.67E-04)	-1.00 (9.90E-01)	-2.21 (5.38E-04)
Fbln2	fibulin 2	2.04 (1.67E-03)	1.43 (2.92E-01)	-1.43 (8.42E-02)
Hbegf	heparin-binding EGF-like growth factor	2.02 (9.19E-04)	1.19 (6.72E-01)	-1.69 (9.32E-03)
Ace	angiotensin I converting enzyme (peptidyl-dipeptidase A) 1	1.94 (4.71E-04)	1.20 (5.47E-01)	-1.62 (6.00E-03)
Plat	plasminogen activator, tissue	1.79 (1.06E-03)	1.06 (8.78E-01)	-1.69 (4.35E-03)
Xirp1	xin actin-binding repeat containing 1	1.59 (1.21E-02)	1.01 (9.86E-01)	-1.58 (1.93E-02)
Mfap2	microfibrillar-associated protein 2	1.54 (2.11E-03)	1.21 (4.11E-01)	-1.28 (6.23E-02)
Entpd1	ectonucleoside triphosphate diphosphohydrolase 1	1.51 (1.14E-02)	1.33 (2.66E-01)	-1.13 (5.68E-01)
Egr1	early growth response 1	1.45 (5.96E-01)	-1.93 (6.04E-01)	-2.80 (9.68E-02)

Gene symbol	Gene name	Fold change (*P*-value)		
		AAV9-GFP TAC *vs* Con	AAV9-EDI TAC *vs* Con	AAV9-EDI TAC *vs* AAV9-GFP TAC
Fbn2	fibrillin 2	1.34 (1.12E-01)	-1.24 (6.01E-01)	-1.66 (1.39E-02)

Supplementary Table 8

Regulated genes associated with extracellular matrix in wild-type mice 4 weeks after TAC surgery and AAV9-EDI treatment. The table refers to the heat map shown in Supplementary Figure 7B; genes reported by Gene Ontology as associated with extracellular matrix and regulated in untreated wild-type (Wt, n=3) versus AAV9-EDI (n=4) or AAV9-GFP (n=4) treated mice after transverse aortic constriction (TAC; 4 weeks). The table indicates the gene symbol, gene name and fold-changes and *p*-values; *n*-numbers represent samples of individual mice. For statistical analysis ANOVA with Tukey *post hoc* test was applied. Source data are provided under the following link: https://www.ebi.ac.uk/arrayexpress/experiments/E-MTAB-8110.

Supplementary Table 9
Regulated genes associated with cell death in wild-type mice 4 weeks after TAC surgery and AAV9-EDI treatment. Table refers to the heat map shown in Supplementary Figure 7B.

Gene symbol	Gene name	Fold change (*P*-value)		
		AAV9-GFP TAC *vs* Con	AAV9-EDI TAC *vs* Con	AAV9-EDI TAC *vs* AAV9-GFP TAC
Postn	periostin, osteoblast specific factor	8.34 (1.70E-05)	1.89 (1.20E-01)	-4.40 (4.37E-04)
Myh7	myosin, heavy polypeptide 7, cardiac muscle, beta	6.20 (7.95E-04)	4.53 (3.11E-02)	-1.37 (6.30E-01)
Thbs1	thrombospondin 1	5.55 (4.48E-05)	-1.01 (9.85E-01)	-5.63 (3.23E-04)
Ctgf	connective tissue growth factor	4.84 (3.12E-05)	1.34 (4.91E-01)	-3.61 (4.21E-04)
Rcan1	regulator of calcineurin 1	3.15 (1.02E-04)	1.09 (8.56E-01)	-2.88 (5.38E-04)
Bgn	biglycan	3.10 (7.20E-05)	1.33 (4.12E-01)	-2.32 (1.11E-03)
Spp1	secreted phosphoprotein 1	3.08 (1.65E-02)	-1.08 (9.48E-01)	-3.33 (1.78E-02)
Col1a1	collagen, type I, alpha 1	3.04 (7.57E-04)	-1.11 (8.78E-01)	-3.38 (1.06E-03)
Sfrp2	secreted frizzled-related protein 2	2.97 (7.65E-03)	1.23 (8.25E-01)	-2.41 (3.11E-02)
Vcan	versican	2.92 (1.11E-04)	1.10 (8.39E-01)	-2.66 (5.81E-04)
Fn1	fibronectin 1	2.65 (2.29E-04)	1.06 (9.05E-01)	-2.49 (9.27E-04)
Nox4	NADPH oxidase 4	2.62 (4.87E-03)	1.49 (5.18E-01)	-1.76 (9.01E-02)
Cyr61	cysteine rich protein 61	2.60 (1.05E-03)	-1.02 (9.81E-01)	-2.64 (1.88E-03)
Tgfb2	transforming growth factor, beta 2	2.48 (4.20E-03)	1.04 (9.60E-01)	-2.39 (8.45E-03)
Stc1	stanniocalcin 1	2.39 (8.01E-05)	1.51 (8.09E-02)	-1.58 (7.89E-03)
Igfbp7	insulin-like growth factor binding protein 7	2.35 (1.28E-05)	1.14 (5.47E-01)	-2.07 (2.39E-04)

Gene symbol	Gene name	Fold change (*P*-value)		
		AAV9-GFP TAC *vs* Con	AAV9-EDI TAC *vs* Con	AAV9-EDI TAC *vs* AAV9-GFP TAC
Col4a1	collagen, type IV, alpha 1	2.29 (3.77E-04)	-1.08 (8.62E-01)	-2.48 (5.97E-04)
Nupr1	nuclear protein transcription regulator 1	2.28 (5.80E-03)	1.10 (8.96E-01)	-2.08 (1.74E-02)
Serpine1	serine (or cysteine) peptidase inhibitor, clade E, member 1	2.28 (1.23E-02)	-1.22 (8.02E-01)	-2.79 (6.36E-03)
Sulf1	sulfatase 1	2.23 (2.59E-04)	1.11 (8.07E-01)	-2.01 (1.53E-03)
Anxa1	annexin A1	2.18 (1.49E-04)	1.21 (5.06E-01)	-1.80 (1.80E-03)
Pla2g4a	phospholipase A2, group IVA (cytosolic, calcium-dependent)	2.13 (1.63E-03)	1.01 (9.86E-01)	-2.11 (3.32E-03)
Sparc	secreted acidic cysteine rich glycoprotein	2.10 (1.41E-04)	-1.04 (9.13E-01)	-2.19 (4.38E-04)
Casp12	caspase 12	2.06 (6.50E-03)	1.29 (6.47E-01)	-1.59 (7.32E-02)
Lrp1	low density lipoprotein receptor-related protein 1	2.04 (6.97E-03)	1.12 (8.47E-01)	-1.82 (2.47E-02)
Col4a2	collagen, type IV, alpha 2	2.04 (5.00E-04)	-1.02 (9.66E-01)	-2.07 (1.06E-03)
Pmepa1	prostate transmembrane protein, androgen induced 1	2.03 (6.02E-04)	1.05 (9.11E-01)	-1.94 (1.86E-03)
Igfbp6	insulin-like growth factor binding protein 6	2.03 (1.79E-02)	1.40 (5.96E-01)	-1.45 (2.55E-01)
Hbegf	heparin-binding EGF-like growth factor	2.02 (9.19E-04)	1.19 (6.72E-01)	-1.69 (9.32E-03)
Nuak1	NUAK family, SNF1-like kinase, 1	2.00 (1.84E-04)	1.11 (7.58E-01)	-1.81 (1.14E-03)
Tgfb3	transforming growth factor, beta 3	1.96 (2.90E-04)	1.15 (6.73E-01)	-1.70 (2.54E-03)
Tlr4	toll-like receptor 4	1.90 (1.81E-02)	1.23 (7.38E-01)	-1.54 (1.28E-01)
Bcl2	B cell leukemia/lymphoma 2	1.90 (1.10E-03)	1.06 (8.96E-01)	-1.80 (3.71E-03)
Haus8	4HAUS augmin-like complex, subunit 8	1.90 (4.97E-03)	1.15 (7.78E-01)	-1.65 (2.54E-02)

Gene symbol	Gene name	Fold change (*P*-value)		
		AAV9-GFP TAC *vs* Con	AAV9-EDI TAC *vs* Con	AAV9-EDI TAC *vs* AAV9-GFP TAC
Plat	plasminogen activator, tissue	1.79 (1.06E-03)	1.06 (8.78E-01)	-1.69 (4.35E-03)
Rtn4	reticulon 4	1.76 (1.28E-03)	1.05 (8.90E-01)	-1.67 (4.19E-03)
Vim	vimentin	1.75 (7.20E-05)	1.01 (9.75E-01)	-1.74 (4.27E-04)
Lum	lumican	1.73 (8.97E-05)	1.11 (6.05E-01)	-1.55 (9.27E-04)
Cyp 1b1	cytochrome P450, family 1, subfamily b, polypeptide 1	1.70 (2.59E-03)	1.09 (8.13E-01)	-1.55 (1.29E-02)
Mmp 14	matrix metallopeptidase 14 (membrane-inserted)	1.67 (1.17E-03)	-1.05 (8.95E-01)	-1.75 (1.52E-03)
Plcg2	phospholipase C, gamma 2	1.64 (1.98E-03)	1.11 (7.55E-01)	-1.48 (1.31E-02)
Tnip1	TNFAIP3 interacting protein 1	1.60 (3.18E-03)	1.13 (7.18E-01)	-1.42 (2.53E-02)
Axl	AXL receptor tyrosine kinase	1.58 (8.73E-04)	1.14 (6.08E-01)	-1.39 (1.09E-02)
Mvp	major vault protein	1.56 (1.06E-02)	1.09 (8.32E-01)	-1.43 (4.29E-02)
Sema 3f	sema domain, immunoglobulin domain (Ig), short basic domain, secreted, (semaphorin) 3F	1.55 (6.14E-03)	-1.00 (9.98E-01)	-1.55 (8.93E-03)
Ccdc 80	coiled-coil domain containing 80	1.53 (9.39E-03)	1.08 (8.39E-01)	-1.42 (3.52E-02)
Serpin g1	serine (or cysteine) peptidase inhibitor, clade G, member 1	1.49 (8.98E-02)	1.17 (7.72E-01)	-1.27 (4.17E-01)
Ccnd1	cyclin D1	1.45 (2.35E-01)	-1.27 (7.30E-01)	-1.84 (5.44E-02)
Cfh	complement component factor h	1.43 (3.38E-01)	1.20 (8.28E-01)	-1.19 (7.66E-01)
Creb 3l2	cAMP responsive element binding protein 3-like 2	1.42 (1.61E-02)	-1.06 (8.70E-01)	-1.50 (1.06E-02)
Rhoc	ras homolog family member C	1.41 (1.11E-01)	-1.27 (6.20E-01)	-1.78 (1.56E-02)
Grk5	G protein-coupled receptor kinase 5	1.34 (4.23E-01)	-1.16 (8.55E-01)	-1.55 (2.30E-01)

Gene symbol	Gene name	Fold change (*P*-value)		
		AAV9-GFP TAC *vs* Con	AAV9-EDI TAC *vs* Con	AAV9-EDI TAC *vs* AAV9-GFP TAC
Ptgds	prostaglandin D2 synthase (brain)	1.32 (2.52E-01)	1.75 (1.22E-01)	1.33 (2.93E-01)
Herpud1	homocysteine-inducible, endoplasmic reticulum stress-inducible, ubiquitin-like domain member 1	-1.51 (5.38E-02)	1.30 (5.35E-01)	1.96 (6.53E-03)
Camk2a	calcium/calmodulin-dependent protein kinase II alpha	-1.51 (3.07E-04)	-1.25 (1.05E-01)	1.21 (4.65E-02)
As3mt	arsenic (+3 oxidation state) methyltransferase	-1.58 (5.68E-05)	-1.06 (7.57E-01)	1.50 (5.05E-04)
Sirt1	sirtuin 1	-1.61 (6.79E-03)	-1.12 (7.78E-01)	1.45 (3.69E-02)
Myocd	myocardin	-1.64 (7.28E-05)	-1.25 (8.60E-02)	1.31 (5.79E-03)
Zbtb16	zinc finger and BTB domain containing 16	-1.80 (9.37E-03)	1.02 (9.78E-01)	1.82 (1.24E-02)

Supplementary Table 9

Regulated genes associated with cell death in wild-type mice 4 weeks after TAC surgery and AAV9-EDI treatment. The table refers to the heat map shown in Supplementary Figure 7B; genes reported by Gene Ontology as associated with cell death and regulated in untreated wild-type (Wt, n=3) versus AAV9-EDI (n=4) or AAV9-GFP (n=4) treated mice after transverse aortic constriction (TAC; 4 weeks). The table indicates the gene symbol, gene name and fold-changes and *p*-values; *n*-numbers represent samples of individual mice. For statistical analysis ANOVA with Tukey *post hoc* test was applied. Source data are provided under the following link: https://www.ebi.ac.uk/arrayexpress/experiments/E-MTAB-8110.

Supplementary Table 10
Regulated genes associated with heart failure in wild-type mice 4 weeks after TAC surgery and AAV9-EDI treatment. The table refers to the heat map shown in Supplementary Figure 7B.

Gene symbol	Gene name	Fold change (*P*-value)		
		AAV9-GFP TAC *vs* Con	AAV9-EDI TAC *vs* Con	AAV9-EDI TAC *vs* AAV9-GFP TAC
Postn	periostin, osteoblast specific factor	8.34 (1.70E-05)	1.89 (1.20E-01)	-4.40 (4.37E-04)
Comp	cartilage oligomeric matrix protein	6.67 (2.07E-03)	1.17 (9.11E-01)	-5.72 (5.41E-03)
Thbs4	thrombospondin 4	4.96 (1.93E-03)	1.71 (5.68E-01)	-2.89 (2.81E-02)
Ctgf	connective tissue growth factor	4.84 (3.12E-05)	1.34 (4.91E-01)	-3.61 (4.21E-04)
Frzb	frizzled-related protein	3.46 (1.89E-04)	1.30 (6.34E-01)	-2.65 (1.84E-03)
Tnfrsf12a	tumor necrosis factor receptor superfamily, member 12a	3.39 (9.75E-04)	1.84 (2.29E-01)	-1.85 (5.38E-02)
Fstl1	follistatin-like 1	3.21 (6.32E-05)	1.15 (7.63E-01)	-2.79 (5.18E-04)
Lox	lysyl oxidase	2.86 (1.35E-03)	1.22 (7.61E-01)	-2.34 (7.81E-03)
Fn1	fibronectin 1	2.65 (2.29E-04)	1.06 (9.05E-01)	-2.49 (9.27E-04)
Nox4	NADPH oxidase 4	2.62 (4.87E-03)	1.49 (5.18E-01)	-1.76 (9.01E-02)
Cyr61	cysteine rich protein 61	2.60 (1.05E-03)	-1.02 (9.81E-01)	-2.64 (1.88E-03)
Wisp2	WNT1 inducible signaling pathway protein 2	2.59 (1.93E-02)	1.88 (3.63E-01)	-1.38 (5.48E-01)
Tgfb2	transforming growth factor, beta 2	2.48 (4.20E-03)	1.04 (9.60E-01)	-2.39 (8.45E-03)
Fhl1	four and a half LIM domains 1	2.48 (1.32E-03)	1.48 (3.56E-01)	-1.68 (4.29E-02)
Col4a1	collagen, type IV, alpha 1	2.29 (3.77E-04)	-1.08 (8.62E-01)	-2.48 (5.97E-04)
Serpine1	serine (or cysteine) peptidase inhibitor, clade E, member 1	2.28 (1.23E-02)	-1.22 (8.02E-01)	-2.79 (6.36E-03)

Gene symbol	Gene name	Fold change (*P*-value)		
		AAV9-GFP TAC *vs* Con	AAV9-EDI TAC *vs* Con	AAV9-EDI TAC *vs* AAV9-GFP TAC
Mmp2	matrix metallopeptidase 2	2.06 (1.45E-03)	1.06 (9.08E-01)	-1.94 (4.52E-03)
Fbln2	fibulin 2	2.04 (1.67E-03)	1.43 (2.92E-01)	-1.43 (8.42E-02)
Tgfb3	transforming growth factor, beta 3	1.96 (2.90E-04)	1.15 (6.73E-01)	-1.70 (2.54E-03)
Bcl2	B cell leukemia/lymphoma 2	1.90 (1.10E-03)	1.06 (8.96E-01)	-1.80 (3.71E-03)
Sfrp1	secreted frizzled-related protein 1	1.73 (1.80E-02)	1.03 (9.54E-01)	-1.67 (3.41E-02)
Lum	lumican	1.73 (8.97E-05)	1.11 (6.05E-01)	-1.55 (9.27E-04)
P4ha1	procollagen-proline, 2-oxoglutarate 4-dioxygenase (proline 4-hydroxylase), alpha 1 polypeptide	1.69 (7.04E-03)	1.04 (9.43E-01)	-1.63 (1.56E-02)
Tgm2	transglutaminase 2, C polypeptide	1.64 (4.94E-04)	1.29 (1.59E-01)	-1.27 (4.71E-02)
Stat3	signal transducer and activator of transcription 3	1.48 (2.70E-05)	1.05 (7.14E-01)	-1.41 (3.23E-04)
Dusp6	dual specificity phosphatase 6	1.43 (1.15E-01)	-1.11 (8.49E-01)	-1.59 (5.68E-02)
Grk5	G protein-coupled receptor kinase 5	1.34 (4.23E-01)	-1.16 (8.55E-01)	-1.55 (2.30E-01)
Maob	monoamine oxidase B	-2.14 (6.74E-05)	-1.20 (4.40E-01)	1.79 (9.70E-04)
Rgs2	regulator of G-protein signaling 2	-2.21 (1.61E-03)	-1.77 (8.71E-02)	1.25 (3.97E-01)
Rbfox1	RNA binding protein, fox-1 homolog (C. elegans) 1	-2.54 (3.50E-05)	-1.51 (4.31E-02)	1.68 (1.89E-03)

Supplementary Table 10

Regulated genes associated with heart failure in wild-type mice 4 weeks after TAC surgery and AAV9-EDI treatment. The table refers to the heat map shown in Supplementary Figure 7B; genes reported by Gene Ontology as associated with heart failure and regulated in untreated wild-type (Wt, n=3) versus AAV9-EDI (n=4) or AAV9-GFP (n=4) treated mice after transverse aortic constriction (TAC; 4 weeks). The table indicates the gene symbol, gene name and fold-changes and *p*-values; *n*-numbers represent samples of individual mice. For statistical analysis ANOVA with Tukey *post hoc* test was applied. Source data are provided under the following link: https://www.ebi.ac.uk/arrayexpress/experiments/E-MTAB-8110.

Supplementary table 11
Regulated genes associated with Myc-related gene networks in wild-type mice 4 weeks after TAC surgery and AAV9-EDI treatment. Table refers to the heat map shown in Supplementary Figure 7D.

Gene symbol	Gene name	Fold change (*P*-value)		
		AAV9-GFP TAC *vs* Con	AAV9-EDI TAC *vs* Con	AAV9-EDI TAC *vs* AAV9-GFP TAC
Myh7	myosin. heavy polypeptide 7. cardiac muscle. beta	6.20 (7.95E-04)	4.53 (3.11E-02)	-1.37 (6.30E-01)
Timp1	tissue inhibitor of metalloproteinase 1	5.74 (2.19E-04)	1.42 (6.74E-01)	-4.05 (1.88E-03)
Thbs1	thrombospondin 1	5.55 (4.48E-05)	-1.01 (9.85E-01)	-5.63 (3.23E-04)
Acta1	actin. alpha 1. skeletal muscle	4.49 (5.78E-06)	3.31 (5.69E-04)	-1.36 (6.62E-02)
Fmod	fibromodulin	4.42 (8.99E-04)	1.17 (8.69E-01)	-3.79 (3.21E-03)
Gck	glucokinase	3.97 (1.84E-04)	3.19 (1.24E-02)	-1.24 (5.37E-01)
Itgbl1	integrin. beta-like 1	3.94 (3.00E-04)	1.65 (3.19E-01)	-2.38 (8.51E-03)
Nppb	natriuretic peptide type B	3.80 (1.55E-03)	1.86 (3.25E-01)	-2.04 (5.75E-02)
Nppa	natriuretic peptide type A	3.74 (3.31E-05)	2.12 (1.26E-02)	-1.77 (6.44E-03)
Egr2	early growth response 2	3.72 (5.47E-03)	-1.03 (9.78E-01)	-3.83 (7.52E-03)
Frzb	frizzled-related protein	3.46 (1.89E-04)	1.30 (6.34E-01)	-2.65 (1.84E-03)
Mt2	metallothionein 2	3.37 (2.39E-03)	1.41 (6.74E-01)	-2.38 (2.38E-02)
Fbn1	fibrillin 1	3.33 (1.49E-04)	1.05 (9.39E-01)	-3.18 (5.38E-04)
Fstl1	follistatin-like 1	3.21 (6.32E-05)	1.15 (7.63E-01)	-2.79 (5.18E-04)
Emp1	epithelial membrane protein 1	3.10 (3.50E-05)	1.16 (6.94E-01)	-2.68 (4.03E-04)
Spp1	secreted phosphoprotein 1	3.08 (1.65E-02)	-1.08 (9.48E-01)	-3.33 (1.78E-02)
Cebpd	CCAAT/enhancer binding protein (C/EBP). delta	2.96 (4.84E-04)	1.48 (3.84E-01)	-2.00 (1.31E-02)

Gene symbol	Gene name	Fold change (*P*-value)		
		AAV9-GFP TAC *vs* Con	AAV9-EDI TAC *vs* Con	AAV9-EDI TAC *vs* AAV9-GFP TAC
Lox	lysyl oxidase	2.86 (1.35E-03)	1.22 (7.61E-01)	-2.34 (7.81E-03)
Uck2	uridine-cytidine kinase 2	2.83 (1.49E-04)	1.19 (7.11E-01)	-2.38 (1.21E-03)
Rbp1	retinol binding protein 1. cellular	2.82 (2.45E-03)	1.11 (8.93E-01)	-2.54 (7.66E-03)
Fn1	fibronectin 1	2.65 (2.29E-04)	1.06 (9.05E-01)	-2.49 (9.27E-04)
Ddit4	DNA-damage-inducible transcript 4	2.64 (1.02E-02)	1.57 (5.37E-01)	-1.68 (1.86E-01)
Tnc	tenascin C	2.58 (9.19E-04)	1.06 (9.24E-01)	-2.44 (2.50E-03)
Tgfb2	transforming growth factor. beta 2	2.48 (4.20E-03)	1.04 (9.60E-01)	-2.39 (8.45E-03)
Adamts 1	a disintegrin-like and metallopeptidase (reprolysin type) with thrombospondin type 1 motif. 1	2.28 (1.92E-04)	1.41 (2.04E-01)	-1.61 (9.71E-03)
Serpine 1	serine (or cysteine) peptidase inhibitor. clade E. member 1	2.28 (1.23E-02)	-1.22 (8.02E-01)	-2.79 (6.36E-03)
Cst6	cystatin E/M	2.22 (3.38E-03)	1.47 (3.80E-01)	-1.51 (1.12E-01)
Mgp	matrix Gla protein	2.22 (1.15E-03)	1.41 (3.45E-01)	-1.57 (3.94E-02)
Ccnb1	cyclin B1	2.18 (6.79E-03)	1.06 (9.37E-01)	-2.06 (1.54E-02)
Cdk1	cyclin-dependent kinase 1	2.13 (1.33E-02)	1.08 (9.16E-01)	-1.97 (3.09E-02)
Myc	myelocytomatosis oncogene	2.12 (1.11E-02)	1.24 (7.46E-01)	-1.71 (6.87E-02)
Sparc	secreted acidic cysteine rich glycoprotein	2.10 (1.41E-04)	-1.04 (9.13E-01)	-2.19 (4.38E-04)
Pmp22	peripheral myelin protein 22	2.05 (5.60E-05)	1.09 (7.46E-01)	-1.87 (5.09E-04)
Fbln2	fibulin 2	2.04 (1.67E-03)	1.43 (2.92E-01)	-1.43 (8.42E-02)
Csrp2	cysteine and glycine-rich protein 2	2.00 (1.69E-03)	1.01 (9.78E-01)	-1.97 (3.50E-03)

Gene symbol	Gene name	Fold change (*P*-value)		
		AAV9-GFP TAC *vs* Con	AAV9-EDI TAC *vs* Con	AAV9-EDI TAC *vs* AAV9-GFP TAC
Bcl2	B cell leukemia/lymphoma 2	1.90 (1.10E-03)	1.06 (8.96E-01)	-1.8 (3.71E-03)
Ccna2	cyclin A2	1.90 (1.95E-02)	-1.05 (9.47E-01)	-1.99 (1.91E-02)
Ccl6	chemokine (C-C motif) ligand 6	1.88 (3.18E-02)	1.49 (4.69E-01)	-1.26 (5.61E-01)
Uchl1	ubiquitin carboxy-terminal hydrolase L1	1.88 (6.93E-03)	1.19 (7.30E-01)	-1.58 (4.64E-02)
Ptgs2	prostaglandin-endoperoxide synthase 2	1.85 (7.31E-04)	1.10 (7.81E-01)	-1.68 (4.03E-03)
Icam1	intercellular adhesion molecule 1	1.84 (1.89E-02)	1.16 (8.13E-01)	-1.58 (8.84E-02)
Cd44	CD44 antigen	1.75 (5.83E-03)	-1.05 (9.20E-01)	-1.84 (6.20E-03)
Vim	vimentin	1.75 (7.20E-05)	1.01 (9.75E-01)	-1.74 (4.27E-04)
Cd9	CD9 antigen	1.74 (1.41E-04)	1.18 (3.71E-01)	-1.47 (2.57E-03)
Ecm1	extracellular matrix protein 1	1.74 (1.60E-02)	1.05 (9.38E-01)	-1.67 (3.43E-02)
Lum	lumican	1.73 (8.97E-05)	1.11 (6.05E-01)	-1.55 (9.27E-04)
Sfrp1	secreted frizzled-related protein 1	1.73 (1.80E-02)	1.03 (9.54E-01)	-1.67 (3.41E-02)
Zfp36	zinc finger protein 36	1.73 (1.35E-03)	1.13 (7.23E-01)	-1.54 (1.03E-02)
Asns	asparagine synthetase	1.70 (4.10E-03)	1.25 (5.00E-01)	-1.36 (8.58E-02)
Cdc20	cell division cycle 20	1.70 (3.02E-02)	-1.14 (8.31E-01)	-1.93 (1.55E-02)
Ptprc	protein tyrosine phosphatase. receptor type. C	1.70 (8.53E-02)	1.03 (9.76E-01)	-1.66 (1.21E-01)
Sgk1	serum/glucocorticoid regulated kinase 1	1.70 (8.46E-03)	1.38 (3.27E-01)	-1.23 (3.53E-01)
Lxn	latexin	1.69 (1.45E-02)	-1.03 (9.50E-01)	-1.75 (1.48E-02)

Gene symbol	Gene name	Fold change (*P*-value)		
		AAV9-GFP TAC *vs* Con	AAV9-EDI TAC *vs* Con	AAV9-EDI TAC *vs* AAV9-GFP TAC
Anxa5	annexin A5	1.67 (6.42E-04)	1.08 (7.96E-01)	-1.54 (3.58E-03)
Birc5	baculoviral IAP repeat-containing 5	1.67 (8.02E-02)	-1.16 (8.30E-01)	-1.95 (3.39E-02)
Pycr1	pyrroline-5-carboxylate reductase 1	1.66 (4.47E-02)	1.12 (8.51E-01)	-1.48 (1.43E-01)
Inhba	inhibin beta-A	1.65 (8.15E-04)	1.09 (7.74E-01)	-1.51 (4.91E-03)
Itga1	integrin alpha 1	1.65 (1.31E-03)	-1.01 (9.68E-01)	-1.67 (2.05E-03)
Tspo	translocator protein	1.65 (3.49E-02)	1.03 (9.56E-01)	-1.60 (6.01E-02)
Crip1	cysteine-rich protein 1 (intestinal)	1.64 (2.34E-04)	1.14 (5.09E-01)	-1.44 (3.33E-03)
Nfkbia	nuclear factor of kappa light polypeptide gene enhancer in B cells inhibitor. alpha	1.60 (5.61E-03)	1.08 (8.44E-01)	-1.48 (2.03E-02)
Tagln2	transgelin 2	1.60 (9.24E-04)	-1.02 (9.47E-01)	-1.64 (1.55E-03)
Serpine 2	serine (or cysteine) peptidase inhibitor. clade E. member 2	1.59 (7.27E-02)	-1.22 (7.49E-01)	-1.93 (1.99E-02)
Axl	AXL receptor tyrosine kinase	1.58 (8.73E-04)	1.14 (6.08E-01)	-1.39 (1.09E-02)
Fbln5	fibulin 5	1.58 (1.54E-03)	1.16 (5.98E-01)	-1.37 (2.31E-02)
Klf4	Kruppel-like factor 4 (gut)	1.58 (1.50E-03)	1.20 (4.43E-01)	-1.32 (3.67E-02)
Pam	peptidylglycine alpha-amidating monooxygenase	1.58 (3.95E-05)	1.25 (3.96E-02)	-1.26 (4.52E-03)
Ctdspl	CTD (carboxy-terminal domain. RNA polymerase II. polypeptide A) small phosphatase-like	1.56 (1.80E-02)	1.07 (8.81E-01)	-1.46 (5.57E-02)
Alb	albumin	1.54 (2.73E-01)	-1.04 (9.68E-01)	-1.61 (2.70E-01)
Clu	clusterin	1.54 (5.26E-03)	1.24 (3.93E-01)	-1.24 (1.71E-01)
Hmox1	heme oxygenase 1	1.53 (7.02E-03)	1.28 (3.45E-01)	-1.20 (2.86E-01)

Gene symbol	Gene name	Fold change (*P*-value)		
		AAV9-GFP TAC *vs* Con	AAV9-EDI TAC *vs* Con	AAV9-EDI TAC *vs* AAV9-GFP TAC
Smad1	SMAD family member 1	1.53 (3.08E-03)	-1.11 (7.34E-01)	-1.70 (1.66E-03)
Abcc3	ATP-binding cassette. sub-family C (CFTR/MRP). member 3	1.51 (2.22E-03)	1.18 (4.49E-01)	-1.27 (5.25E-02)
Mrc1	mannose receptor. C type 1	1.51 (1.22E-02)	1.01 (9.83E-01)	-1.5 (2.01E-02)
Tns3	tensin 3	1.50 (7.00E-04)	1.03 (9.17E-01)	-1.46 (1.88E-03)
Actn1	actinin. alpha 1	1.49 (5.90E-04)	1.01 (9.73E-01)	-1.48 (1.52E-03)
Dbn1	drebrin 1	1.49 (4.01E-03)	1.07 (8.17E-01)	-1.39 (1.88E-02)
Oasl2	2'-5' oligoadenylate synthetase-like 2	1.49 (2.55E-01)	-1.24 (7.96E-01)	-1.84 (8.99E-02)
Ccnb2	cyclin B2	1.48 (2.42E-01)	-1.19 (8.24E-01)	-1.77 (8.99E-02)
Mt1	metallothionein 1	1.48 (3.00E-02)	1.28 (4.92E-01)	-1.16 (5.51E-01)
Mcm6	minichromosome maintenance complex component 6	1.46 (1.96E-01)	-1.23 (7.66E-01)	-1.80 (5.31E-02)
Ccnd1	cyclin D1	1.45 (2.35E-01)	-1.27 (7.30E-01)	-1.84 (5.44E-02)
Egr1	early growth response 1	1.45 (5.96E-01)	-1.93 (6.04E-01)	-2.80 (9.68E-02)
Rrm2	ribonucleotide reductase M2	1.45 (1.99E-01)	-1.20 (7.99E-01)	-1.74 (6.71E-02)
Serpinh1	serine (or cysteine) peptidase inhibitor. clade H. member 1	1.45 (4.18E-02)	-1.30 (4.24E-01)	-1.89 (3.84E-03)
Bcl6	B cell leukemia/lymphoma 6	1.43 (1.21E-01)	-1.23 (7.09E-01)	-1.76 (2.59E-02)
Cald1	caldesmon 1	1.43 (6.61E-03)	-1.12 (6.89E-01)	-1.6 (2.18E-03)
Dusp6	dual specificity phosphatase 6	1.43 (1.15E-01)	-1.11 (8.49E-01)	-1.59 (5.68E-02)
Fap	fibroblast activation protein	1.43 (8.53E-02)	-1.16 (7.71E-01)	-1.66 (2.55E-02)

Gene symbol	Gene name	Fold change (*P*-value)		
		AAV9-GFP TAC *vs* Con	AAV9-EDI TAC *vs* Con	AAV9-EDI TAC *vs* AAV9-GFP TAC
Chrnb1	cholinergic receptor. nicotinic. beta polypeptide 1 (muscle)	1.40 (3.33E-02)	-1.20 (6.05E-01)	-1.68 (5.35E-03)
Ptn	pleiotrophin	1.40 (1.52E-01)	-1.12 (8.44E-01)	-1.56 (6.97E-02)
Akap12	A kinase (PRKA) anchor protein (gravin) 12	1.39 (1.27E-02)	-1.17 (5.43E-01)	-1.62 (2.27E-03)
Flna	filamin. alpha	1.38 (4.61E-02)	-1.16 (6.90E-01)	-1.60 (9.78E-03)
Wisp1	WNT1 inducible signaling pathway protein 1	1.36 (2.80E-01)	-1.18 (7.93E-01)	-1.61 (9.79E-02)
Usp18	ubiquitin specific peptidase 18	1.35 (2.49E-01)	-1.19 (7.71E-01)	-1.61 (7.10E-02)
Casp8	caspase 8	1.34 (1.48E-01)	-1.17 (7.50E-01)	-1.57 (3.50E-02)
Fbn2	fibrillin 2	1.34 (1.12E-01)	-1.24 (6.01E-01)	-1.66 (1.39E-02)
Itga6	integrin alpha 6	1.34 (7.79E-02)	-1.31 (3.66E-01)	-1.76 (4.31E-03)
Fos	FBJ osteosarcoma oncogene	1.17 (8.25E-01)	-1.94 (5.43E-01)	-2.28 (1.48E-01)
Klf10	Kruppel-like factor 10	-1.16 (6.50E-01)	-1.51 (4.08E-01)	-1.30 (4.53E-01)
Mgst1	microsomal glutathione S-transferase 1	-1.27 (4.79E-01)	1.21 (7.83E-01)	1.53 (1.76E-01)
Usp54	ubiquitin specific peptidase 54	-1.35 (6.26E-02)	1.11 (7.83E-01)	1.50 (2.16E-02)
Got1	glutamic-oxaloacetic transaminase 1. soluble	-1.40 (4.01E-02)	1.17 (6.73E-01)	1.64 (8.26E-03)
Cobll1	Cobl-like 1	-1.43 (5.09E-02)	1.06 (8.93E-01)	1.52 (3.17E-02)
Fkbp4	FK506 binding protein 4	-1.43 (1.28E-03)	-1.08 (7.37E-01)	1.33 (8.95E-03)
Noct	nocturnin	-1.43 (7.49E-02)	1.05 (9.20E-01)	1.51 (5.64E-02)
Idh1	isocitrate dehydrogenase 1 (NADP+). soluble	-1.45 (9.78E-03)	1.18 (5.26E-01)	1.71 (1.75E-03)

Gene symbol	Gene name	Fold change (*P*-value)		
		AAV9-GFP TAC *vs* Con	AAV9-EDI TAC *vs* Con	AAV9-EDI TAC *vs* AAV9-GFP TAC
Bckdhb	branched chain ketoacid dehydrogenase E1. beta polypeptide	-1.54 (1.84E-04)	-1.05 (8.33E-01)	1.47 (1.04E-03)
Pfas	phosphoribosylformylglycinamidine synthase (FGAR amidotransferase)	-1.54 (3.57E-03)	-1.28 (2.70E-01)	1.2 (2.24E-01)
Ezr	ezrin	-1.55 (1.59E-04)	-1.34 (2.64E-02)	1.15 (1.14E-01)
Parp1	poly (ADP-ribose) polymerase family. member 1	-1.55 (1.00E-03)	-1.19 (3.94E-01)	1.30 (2.89E-02)
Dusp7	dual specificity phosphatase 7	-1.56 (7.30E-05)	-1.16 (2.42E-01)	1.35 (1.90E-03)
Ppl	periplakin	-1.57 (1.86E-02)	-1.01 (9.84E-01)	1.55 (2.80E-02)
Sumo3	small ubiquitin-like modifier 3	-1.58 (1.23E-02)	-1.29 (4.49E-01)	1.23 (3.31E-01)
Gpt	glutamic pyruvic transaminase. soluble	-1.63 (6.47E-04)	1.01 (9.86E-01)	1.64 (1.35E-03)
Acss1	acyl-CoA synthetase short-chain family member 1	-1.68 (1.73E-03)	1.01 (9.86E-01)	1.69 (2.91E-03)
Irx3	Iroquois related homeobox 3	-1.69 (8.78E-03)	-1.48 (1.85E-01)	1.14 (6.44E-01)
Bmper	BMP-binding endothelial regulator	-1.71 (1.58E-02)	-1.16 (7.83E-01)	1.48 (8.35E-02)
F3	coagulation factor III	-1.77 (3.30E-03)	-1.19 (6.65E-01)	1.49 (3.65E-02)
Ppat	phosphoribosyl pyrophosphate amidotransferase	-1.79 (1.71E-04)	-1.06 (8.30E-01)	1.69 (9.27E-04)
Rtn2	reticulon 2 (Z-band associated protein)	-1.81 (5.78E-06)	-1.32 (9.67E-03)	1.37 (7.90E-04)
H19	H19. imprinted maternally expressed transcript	-1.97 (5.26E-04)	-1.69 (3.11E-02)	1.17 (4.31E-01)
Efemp1	epidermal growth factor-containing fibulin-like extracellular matrix protein 1	-2.15 (2.84E-02)	-1.34 (7.18E-01)	1.61 (2.14E-01)
Lamb3	laminin. beta 3	-2.74 (2.78E-05)	-1.35 (1.74E-01)	2.03 (5.38E-04)
Pfkfb1	6-phosphofructo-2-kinase/fructose-2.6-biphosphatase 1	-3.66 (2.50E-05)	-1.29 (4.13E-01)	2.83 (3.89E-04)

Gene symbol	Gene name	Fold change (*P*-value)		
		AAV9-GFP TAC *vs* Con	AAV9-EDI TAC *vs* Con	AAV9-EDI TAC *vs* AAV9-GFP TAC
Angpt1	angiopoietin 1	-5.35 (2.78E-05)	-2.20 (2.64E-02)	2.44 (2.04E-03)

Supplementary Table 11

Regulated genes associated with Myc-related gene networks in wild-type mice 4 weeks after TAC surgery and AAV9-EDI treatment. The table refers to the heat map shown in Supplementary Figure 7D; genes reported by Gene Ontology as associated with Myc-related gene networks and regulated in untreated wild-type (Wt, n=3) versus AAV9-EDI (n=4) or AAV9-GFP (n=4) treated mice after transverse aortic constriction (TAC; 4 weeks). The table indicates the gene symbol, gene name and fold-changes and *p*-values; *n*-numbers represent samples of individual mice. For statistical analysis ANOVA with Tukey *post hoc* test was applied. Source data are provided under the following link: https://www.ebi.ac.uk/arrayexpress/experiments/E-MTAB-8110.

Supplementary Table 12
Regulated genes associated with NFAT-related gene networks in wild-type mice 4 weeks after TAC surgery and AAV9-EDI treatment. Table refers to the heat map shown in Supplementary Figure 7D.

		**Fold change (p-value)**		
**Gene symbol**	**Gene name**	AAV9-GFP TAC vs Con	AAV9-EDI TAC vs Con	AAV9-EDI TAC vs AAV9-GFP TAC
Myh7	myosin, heavy polypeptide 7, cardiac muscle, beta	6.20 (7.95E-05)	4.53 (3.11E-2)	-1.37 (6.30E-1)
Gdf15	growth differentiation factor 15	5.77 (1.40E-3)	1.3 (8.18E-1)	-4.43 (6.40E-3)
Ctgf	connective tissue growth factor	4.84 (3.12E-5)	1.34 (4.91E-1)	-3.61 (4.21E-4)
Acta1	actin, alpha 1, skeletal muscle	4.49 (5.78E-6)	3.31 (5.69E-4)	-1.36 (6.62E-2)
Nppb	natriuretic peptide type B	3.8 (1.55E-3)	1.86 (3.25E-1)	-2.04 (5.75E-2)
Nppa	natriuretic peptide type A	3.74 (3.31E-5)	2.12 (1.26E-2)	-1.77 (6.44E-3)
Egr2	early growth response 2	3.72 (5.47E-3)	-1.03 (9.78E-1)	-3.83 (7.52E-3)
Ccl8	chemokine (C-C motif) ligand 8	3.43 (2.27E-3)	1.8 (3.60E-1)	-1.91 (8.90E-2)
Rcan1	regulator of calcineurin 1	3.15 (1.02E-4)	1.09 (8.56E-1)	-2.88 (5.38E-4)
Spp1	secreted phosphoprotein 1	3.08 (1.65E-2)	-1.08 (9.48E-1)	-3.33 (1.78E-2)
Fn1	fibronectin 1	2.65 (2.29E-4)	1.06 (9.05E-1)	-2.49 (9.27E-4)
Ddit4	DNA-damage-inducible transcript 4	2.64 (1.02E-2)	1.57 (5.37E-1)	-1.68 (1.86E-1)
Nox4	NADPH oxidase 4	2.62 (4.87E-3)	1.49 (5.18E-1)	-1.76 (9.01E-2)
Cyr61	cysteine rich protein 61	2.6 (1.05E-3)	-1.02 (9.81E-1)	-2.64 (1.88E-3)
Fhl1	four and a half LIM domains 1	2.48 (1.32E-3)	1.48 (3.56E-1)	-1.68 (4.29E-2)

		Fold change (p-value)		
**Gene symbol**	**Gene name**	AAV9-GFP TAC vs Con	AAV9-EDI TAC vs Con	AAV9-EDI TAC vs AAV9-GFP TAC
Tgfb2	transforming growth factor, beta 2	2.48 (4.20E-3)	1.04 (9.60E-1)	-2.39 (8.45E-3)
Dkk3	dickkopf WNT signaling pathway inhibitor 3	2.43 (5.63E-5)	1.26 (3.66E-1)	-1.93 (9.27E-4)
Serpine 1	serine (or cysteine) peptidase inhibitor, clade E, member 1	2.28 (1.23E-2)	-1.22 (8.02E-1)	-2.79 (6.36E-3)
Eln	elastin	2.20 (1.67E-4)	-1.00 (9.90E-1)	-2.21 (5.38E-4)
Fgl2	fibrinogen-like protein 2	2.2 (8.58E-3)	1.05 (9.47E-1)	-2.1 (1.81E-2)
Il6	interleukin 6	2.16 (1.56E-3)	1.04 (9.47E-1)	-2.09 (3.70E-3)
Cdk1	cyclin-dependent kinase 1	2.13 (1.33E-2)	1.08 (9.16E-1)	-1.97 (3.09E-2)
Myc	myelocytomatosis oncogene	2.12 (1.11E-2)	1.24 (7.46E-1)	-1.71 (6.87E-2)
Mmp2	matrix metallopeptidase 2	2.06 (1.45E-3)	1.06 (9.08E-1)	-1.94 (4.52E-3)
Nfkbiz	nuclear factor of kappa light polypeptide gene enhancer in B cells inhibitor, zeta	2.06 (1.44E-2)	1.01 (9.84E-1)	-2.03 (2.34E-2)
Mapk4	mitogen-activated protein kinase 4	2.05 (1.14E-3)	1.5 (1.78-E-1)	-1.37 (1.14E-1)
Pmp22	peripheral myelin protein 22	2.05 (5.60E-5)	1.09 (7.46E-1)	-1.87 (5.09E-4)
Hbegf	heparin-binding EGF-like growth factor	2.02 (9.19E-4)	1.19 (6.72E-1)	-1.69 (9.32E-3)
Tgfb3	transforming growth factor, beta 3	1.96 (2.90E-4)	1.15 (6.73E-1)	-1.70 (2.54E-3)
Ace	angiotensin I converting enzyme (peptidyl-dipeptidase A) 1	1.94 (4.71E-4)	1.20 (5.47E-1)	-1.62 (6.0E-3)
Bcl2	B cell leukemia/lymphoma 2	1.90 (1.10E-3)	1.06 (8.96E-1)	-1.8 (3.71E-3)
Haus8	4HAUS augmin-like complex, subunit 8	1.90 (4.97E-3)	1.15 (7.78E-1)	-1.65 (2.54E-2)
Tlr4	toll-like receptor 4	1,9 (1.81E-2)	1,23 (7.38E-1)	-1,54 (1.28E-1)

Gene symbol	Gene name	Fold change (p-value) AAV9-GFP TAC vs Con	AAV9-EDI TAC vs Con	AAV9-EDI TAC vs AAV9-GFP TAC
Ptgs2	prostaglandin-endoperoxide synthase 2	1.85 (7.31E-4)	1.1 (7.81E-1)	-1.68 (4.03E-3)
Spc25	SPC25, NDC80 kinetochore complex component, homolog (S. cerevisiae)	1.85 (2.61E-2)	1.08 (9.14E-1)	-1.72 (6.14E-2)
Icam1	intercellular adhesion molecule 1	1.84 (1.89E-2)	1.16 (8.13E-1)	-1.58 (8.84E-2)
Cd14	CD14 antigen	1.83 (3.36E-4)	1.03 (9.38E-1)	-1.78 (1.06E-3)
Ifit2	interferon-induced protein with tetratricopeptide repeats 2	1.82 (8.43E-3)	1.08 (8.90E-1)	-1.69 (2.29E-2)
Atf3	activating transcription factor 3	1.81 (3.91E-2)	1.12 (8.81E-1)	-1.62 (1.09E-1)
Plat	plasminogen activator, tissue	1.79 (0,00106)	1.06 (0,878)	-1.69 (0,00435)
Egr3	early growth response 3	1.77 (2.64E-3)	1.06 (9.00E-1)	-1.68 (7.73E-3)
Vim	vimentin	1.75 (7.20E-5)	1.01 (9.75E-1)	-1.74 (4.27E-4)
Zfp36	zinc finger protein 36	1.73 (1.35E-3)	1.13 (7.23E-1)	-1.54 (1.03E-2)
Lcp1	lymphocyte cytosolic protein 1	1.72 (9.28E-3)	-1.21 (6.98E-1)	-2.08 (2.89E-3)
Mmp3	matrix metallopeptidase 3	1.72 (3.76E-1)	1.65 (7.01E-1)	-1.04 (9.79E-1)
Met	met proto-oncogene	1.71 (9.92E-4)	1.15 (6.66E-1)	-1.49 (1.00E-2)
Cdc20	cell division cycle 20	1.70 (3.02E-2)	-1.14 (8.31E-1)	-1.93 (1.55E-2)
Sgk1	serum/glucocorticoid regulated kinase 1	1.70 (8.46E-3)	1.38 (3.27E-1)	-1.23 (3.53E-1)
Birc5	baculoviral IAP repeat-containing 5	1,67 (8.02E-2)	-1,16 (8.30E-1)	-1,95 (3.39E-2)
Mmp14	matrix metallopeptidase 14 (membrane-inserted)	1,67 (1.17E-3)	-1,05 (8.95E-1)	-1,75 (1.52E-3)
Osmr	oncostatin M receptor	1,67 (4.90E-4)	1,21 (3.83E-1)	-1,38 (1.51E-2)

Gene symbol	Gene name	Fold change (p-value) AAV9-GFP TAC vs Con	AAV9-EDI TAC vs Con	AAV9-EDI TAC vs AAV9-GFP TAC
Socs3	suppressor of cytokine signaling 3	1.67 (8.52E-4)	1.02 (9.59E-1)	-1.64 (2.12E-3)
Inhba	inhibin beta-A	1.65 (8.15E-4)	1.09 (7.74E-1)	-1.51 (4.91E-3)
Itga1	integrin alpha 1	1.65 (1.31E-3)	-1.01 (9.68E-1)	-1.67 (2.05E-3)
Plcg2	phospholipase C, gamma 2	1.64 (1.98E-3)	1.11 (7.55E-1)	-1.48 (1.31E-2)
Tgm2	transglutaminase 2, C polypeptide	1.64 (4.94E-4)	1.29 (1.59E-1)	-1.27 (04.71E-2)
Ctsk	cathepsin K	1.63 (5.49E-3)	1.04 (9.34E-1)	-1.58 (1.30E-2)
Ereg	epiregulin	1.63 (1.79E-3)	-1.03 (9.28E-1)	-1.69 (2.35E-3)
Nfkbia	nuclear factor of kappa light polypeptide gene enhancer in B cells inhibitor, alpha	1.6 (5.61E-3)	1.08 (8.44E-1)	-1.48 (2.03E-2)
Ifitm3	interferon induced transmembrane protein 3	1.58 (4.02E-3)	-1.03 (9.37E-1)	-1.62 (5.19E-3)
Itgb2	integrin beta 2	1.58 (5.74E-3)	1.09 (8.18E-1)	-1.45 (2.53E-2)
Efemp2	epidermal growth factor-containing fibulin-like extracellular matrix protein 2	1,56 (1.19E-2)	1,03 (9.54E-1)	-1,52 (2.54E-2)
Tlr2	toll-like receptor 2	1,55 (6.11E-3)	1,14 (7.00E-1)	-1,35 (5.63E-2)
Cd74	CD74 antigen (invariant polypeptide of major histocompatibility complex, class II antigen-associated)	1,53 (6.06E-2)	1,02 (9.75E-1)	-1,5 (9.01E-2)
Cdca8	cell division cycle associated 8	1,52 (2.61E-2)	-1,03 (9.44E-1)	-1,57 (2.47E-2)
Mrc1	mannose receptor, C type 1	1,51 (1.22E-2)	1,01 (9.83E-1)	-1,5 (2.01E-2)
Rhoj	ras homolog family member J	1,5 (5.52E-2)	-1,1 (8.49E-1)	-1,65 (2.94E-2)
Ptger4	prostaglandin E receptor 4 (subtype EP4)	1,48 (1.15E-1)	1,08 (9.03E-1)	-1,37 (2.62E-1)
C3	complement component 3	1,47 (5.00E-1)	1,46 (7.44E-1)	-1,01 (9.93E-1)

		Fold change (p-value)		
**Gene symbol**	**Gene name**	AAV9-GFP TAC vs Con	AAV9-EDI TAC vs Con	AAV9-EDI TAC vs AAV9-GFP TAC
Jak3	Janus kinase 3	1.46 (5.97E-2)	1.05 (9.20E-1)	-1.38 (1.34E-1)
Ccnd1	cyclin D1	1.45 (2.35E-1)	-1.27 (7.30E-1)	-1.84 (5.44E-2)
Egr1	early growth response 1	1.45 (5.96E-1)	-1.93 (6.04E-1)	-2.8 (9.68E-2)
Rrm2	ribonucleotide reductase M2	1.45 (1.99E-1)	-1.2 (7.99E-1)	-1.74 (6.71E-2)
Aif1	allograft inflammatory factor 1	1.44 (1.80E-1)	-1.25 (7.24E-1)	-1.79 (4.09Ee-2)
Itga9	integrin alpha 9	1.44 (1.92E-2)	-1.08 (8.40E-1)	-1.55 (1.04E-2)
Cfh	complement component factor h	1.43 (3.38E-1)	1.2 (8.28E-1)	-1.19 (7.66E-1)
Dusp6	dual specificity phosphatase 6	1.43 (1.15E-1)	-1.11 (8.49E-1)	-1.59 (5.68E-2)
Cdca3	cell division cycle associated 3	1.41 (4.47E-2)	-1.1 (8.15E-1)	-1.56 (1.90E-2)
Isg20	interferon-stimulated protein	1.41 (2.60E-2)	-1.1 (8.03E-1)	-1.55 (1.03E.2)
Rhoc	ras homolog family member C	1.41 (1.11E-1)	-1.27 (6.20E-1)	-1.78 (1.56E-2)
Cenpe	centromere protein E	1.39 (1.77E-2)	-1.11 (7.30E-1)	-1.55 (5.25E.3)
Cdt1	chromatin licensing and DNA replication factor 1	1.36 (2.88E-1)	-1.21 (7.81E-1)	-1.64 (8.92E-2)
Itga6	integrin alpha 6	1.34 (7.79E-2)	-1.31 (3.66E-1)	-1.76 (4.31E-3)
Nusap1	nucleolar and spindle associated protein 1	1.23 (4.49E-1)	-1.3 (6.29E-1)	-1.6 (6.26E-2)
Cxcl9	chemokine (C-X-C motif) ligand 9	1.18 (6.35E-1)	-1.3 (6.97E-1)	-1.53 (1.63E-1)
Ccl2	chemokine (C-C motif) ligand 2	1.15 (2.98E-1)	-1.02 (9.47E-1)	-1.17 (2.52E-1)
Nfatc4	nuclear factor of activated T cells, cytoplasmic, calcineurin dependent 4	1.12 (4.83E-2)	1.02 (8.94E-1)	-1.09 (1.26E-1)

Gene symbol	Gene name	Fold change (p-value) AAV9-GFP TAC vs Con	AAV9-EDI TAC vs Con	AAV9-EDI TAC vs AAV9-GFP TAC
Csf2	colony stimulating factor 2 (granulocyte-macrophage)	1.11 (5.22E-1)	1.17 (6.07E-1)	1.05 (8.54E-1)
Nfatc2	nuclear factor of activated T cells, cytoplasmic, calcineurin dependent 2	1.03 (5.95E-1)	-1.01 (9.07E-1)	-1.05 (4.52E-1)
Cxcl3	chemokine (C-X-C motif) ligand 3	1.02 (8.57E-1)	1.08 (7.38E-1)	1.05 (7.42E-1)
Nfat5	nuclear factor of activated T cells 5	1.00 (9.69E-1)	1.03 (8.78E-1)	1.02 (8.74E-1)
Nfatc1	nuclear factor of activated T cells, cytoplasmic, calcineurin dependent 1	1.00 (9.73E-1)	-1.01 (9.55E-1)	-1.01 (9.36E-1)
Nfatc3	nuclear factor of activated T cells, cytoplasmic, calcineurin dependent 3	-1.11 (4.55E-1)	-1.07 (8.26E-1)	1.04 (8.90E-1)
Gna12	guanine nucleotide binding protein, alpha 12	-1.40 (4.21E-2)	1.18 (6.45E-1)	1.66 (7.43E-3)
Mapk10	mitogen-activated protein kinase 10	-1.46 (1.88E-3)	1.08 (7.78E-1)	1.57 (1.42E-3)
Lifr	leukemia inhibitory factor receptor	-1.5 (1.11E-3)	-1.07 (7.83E-1)	1.40 (6.29E-3)
Hdac11	histone deacetylase 11	-1.55 (1.96E-4)	-1.20 (1.92E-1)	1.29 (1.14E-2)
Adrb1	adrenergic receptor, beta 1	-1.59 (2.27E-2)	-1.40 (3.19E-1)	1.13 (6.87E-1)
Cacnb2	calcium channel, voltage-dependent, beta 2 subunit	-1.59 (3.87E-3)	-1.03 (9.44E-1)	1.55 (8.97E-3)
Mme	membrane metallo endopeptidase	-1.61 (2.48E-3)	-1.00 (9.96E-1)	1.61 (4.21E-3)
Ctf1	cardiotrophin 1	-1.64 (4.27E-4)	-1.04 (9.00E-1)	1.59 (1.52E-3)
Myocd	myocardin	-1.64 (7.28E-5)	-1.25 (8.60E-2)	1.31 (5.79E-3)
Rgs2	regulator of G-protein signaling 2	-2.21 (1.61E-3)	-1.77 (8.71E-2)	1.25 (3.97E-1)
Kcnd2	potassium voltage-gated channel, Shal-related family, member 2	-2.23 (4.72E-4)	-1.49 (1.74E-1)	1.50 (03.97E-2)
Enpp2	ectonucleotide pyrophosphatase/phosphodiesterase 2	-2.53 (2.84E-3)	-1.25 (7.24E-1)	2.02 (2.01E-2)

Gene symbol	Gene name	Fold change (p-value) AAV9-GFP TAC vs Con	AAV9-EDI TAC vs Con	AAV9-EDI TAC vs AAV9-GFP TAC
Il15	interleukin 15	-3.25 (7.41E-5)	-1.41 (3.01E-1)	2.31 (1.53E-3)

Supplementary Table 12

Regulated genes associated with NFAT-related gene networks in wild-type mice 4 weeks after TAC surgery and AAV9-EDI treatment. The table refers to the heat map shown in Supplementary Figure 7D; genes reported by Gene Ontology as associated with NFAT-related gene networks and regulated in untreated wild-type (Wt, n=3) versus AAV9-EDI (n=4) or AAV9-GFP (n=4) treated mice after transverse aortic constriction (TAC; 4 weeks). The table indicates the gene symbol, gene name and fold-changes and *p*-values; *n*-numbers represent samples of individual mice. For statistical analysis ANOVA with Tukey *post hoc* test was applied. Source data are provided under the following link: https://www.ebi.ac.uk/arrayexpress/experiments/E-MTAB-8110.

Supplementary Table 13
Top 100 regulated genes in LS174T cells transduced with LacZ (Con) or EDI. The table refers to the heat map shown in Fig. 5F.

Gene symbol	Gene name	Fold change (*P*-value)<br>AAV9-EDI *vs* AAV9-LacZ
Kcng2	potassium voltage-gated channel modifier subfamily G member 2	5.80<br>(1.10E-07)
Reg1b	regenerating family member 1 beta	4.18<br>(3.47E-07)
Hist2h2be	histone cluster 2 H2B family member e	3.99<br>(5.69E-06)
Agpat4	1-acylglycerol-3-phosphate O-acyltransferase 4	3.72<br>(2.62E-06)
Fxyd4	FXYD domain containing ion transport regulator 4	3.48<br>(2.89E-05)
Hla-dma	major histocompatibility complex, class II, DM alpha	3.40<br>(3.00E-04)
Tff2	trefoil factor 2	3.40<br>(1.96E-05)
Hist1h2bd	histone cluster 1 H2B family member d	3.24<br>(1.93E-05)
Stc1	stanniocalcin 1	3.24<br>(1.08E-08)
Loc105375773	-	3.12<br>(2.98E-06)
Krt20	keratin 20	3.08<br>(1.94E-03)
Anxa10	annexin A10	3.07<br>(4.47E-04)
Linc00520	long intergenic non-protein coding RNA 520	3.01<br>(2.15E-05)
Dpcr1	diffuse panbronchiolitis critical region 1	2.94<br>(2.69E-04)
Loc102725220	-	2.89<br>(8.29E-05)
Ceacam5	carcinoembryonic antigen related cell adhesion molecule 5	2.81<br>(2.84E-03)
Loc102723684	-	2.76<br>(2.63E-03)
Tm4sf20	transmembrane 4 L six family member 20	2.75<br>(2.15E-05)

Gene symbol	Gene name	Fold change (*P*-value) AAV9-EDI *vs* AAV9-LacZ
Diras2	DIRAS family GTPase 2	2.71 (1.66E-05)
Mmp1	matrix metallopeptidase 1	2.68 (7.74E-05)
Tff1	trefoil factor 1	2.61 (1.04E-03)
Srpx	sushi repeat containing protein, X-linked	2.57 (1.81E-05)
Hist1h3d	histone cluster 1 H3 family member d	2.53 (1.85E-03)
Otub2	OTU deubiquitinase, ubiquitin aldehyde binding 2	2.53 (1.09E-06)
Ceacam6	carcinoembryonic antigen related cell adhesion molecule 6	2.48 (2.00E-03)
Ndrg1	N-myc downstream regulated 1	2.47 (1.33E-05)
Nts	neurotensin	2.45 (5.81E-04)
Dfb1	defensin beta 1	2.42 (3.84E-05)
Capn10-as1	CAPN10 antisense RNA 1 (head to head)	2.42 (7.42E-06)
Hmgcs2	3-hydroxy-3-methylglutaryl-CoA synthase 2	2.34 (1.13E-03)
Mgp	matrix Gla protein	2.20 (3.37E-02)
Ccl26	C-C motif chemokine ligand 26	2.07 (1.05E-02)
Itm2a	integral membrane protein 2A	-2.43 (2.07E-04)
Stil	SCL/TAL1 interrupting locus	-2.43 (7.06E-05)
Kif4a	kinesin family member 4A	-2.44 (1.47E-06)
Psrc1	proline and serine rich coiled-coil 1	-2.46 (4.88E-05)
Mir924hg	MIR924 host gene	-2.48 (8.48E-04)

Gene symbol	Gene name	Fold change (*P*-value) AAV9-EDI *vs* AAV9-LacZ
Ube2c	ubiquitin conjugating enzyme E2 C	-2.49 (2.08E-07)
Sacs	sacsin molecular chaperone	-2.49 (4.17E-07)
Knl1	kinetochore scaffold 1	-2.49 (7.72E-07)
Linc01224	long intergenic non-protein coding RNA 1224	-2.5 (3.17E-05)
Mastl	microtubule associated serine/threonine kinase like	-2.53 (4.12E-05)
Kif2c	kinesin family member 2C	-2.54 (2.10E-06)
Ncapg2	non-SMC condensin II complex subunit G2	-2.54 (6.79E-08)
Cdk1	cyclin dependent kinase 1	-2.56 (3.68E-10)
Cdca8	cell division cycle associated 8	-2.58 (6.82E-08)
Ccdc77	coiled-coil domain containing 77	-2.58 (1.39E-08)
Cenpf	centromere protein F	-2.59 (1.31E-08)
Cdc20	cell division cycle 20	-2.60 (7.22E-07)
Nek2	NIMA related kinase 2	-2.60 (5.17E-07)
Bora	bora, aurora kinase A activator	-2.63 (6.24E-07)
Brip1	BRCA1 interacting protein C-terminal helicase 1	-2.70 (6.52E-07)
Ncaph	non-SMC condensin I complex subunit H	-2.70 (8.75E-08)
C1orf112	chromosome 1 open reading frame 112	-2.70 (2.24E-07)
Prim1	primase (DNA) subunit 1	-2.72 (1.58E-05)
Asf1b	anti-silencing function 1B histone chaperone	-2.72 (7.44E-05)

Gene symbol	Gene name	Fold change (***P***-value)<br>AAV9-EDI *vs* AAV9-LacZ
Ccna2	cyclin A2	-2.72<br>(3.57E-10)
Ascl2	achaete-scute family bHLH transcription factor 2	-2.74<br>(9.50E-08)
Cenpa	centromere protein A	-2.75<br>(1.24E-08)
Aspm	abnormal spindle microtubule assembly	-2.75<br>(1.05E-06)
Gen1	GEN1, Holliday junction 5' flap endonuclease	-2.77<br>(1.39E-04)
Cenph	centromere protein H	-2.79<br>(6.52E-08)
Mnd1	meiotic nuclear divisions 1	-2.80<br>(8.58E-06)
Nmu	neuromedin U	-2.80<br>(1.83E-04)
Pbk	PDZ binding kinase	-2.80<br>(4.04E-09)
Ccnb1	cyclin B1	-2.82<br>(7.49E-08)
Pif1	PIF1 5'-to-3' DNA helicase	-2.84<br>(2.30E-05)
Ttf2	transcription termination factor 2	-2.85<br>(4.87E-06)
Kif23	kinesin family member 23	-2.85<br>(5.69E-06)
Tmed10	transmembrane p24 trafficking protein 10	-2.86<br>(1.85E-08)
Taf5	TATA-box binding protein associated factor 5	-2.87<br>(6.45E-10)
Hjurp	Holliday junction recognition protein	-2.88<br>(1.69E-07)
Kif15	kinesin family member 15	-2.88<br>(8.62E-08)
Rad51ap1	RAD51 associated protein 1	-2.90<br>(1.13E-07)
Arhgap19	Rho GTPase activating protein 19	-2.91<br>(4.38E-08)

Gene symbol	Gene name	Fold change (*P*-value)<br>AAV9-EDI *vs* AAV9-LacZ
Gins1	GINS complex subunit 1	-2.92<br>(1.38E-08)
Depdc1b	DEP domain containing 1B	-2.93<br>(8.71E-06)
Kif20a	kinesin family member 20A	-2.93<br>(2.42E-08)
Trappc4	trafficking protein particle complex 4	-2.94<br>(2.23E-08)
Kif14	kinesin family member 14	-2.99<br>(6.32E-08)
Hmmr	hyaluronan mediated motility receptor	-3.02<br>(5.29E-09)
Tex30	testis expressed 30	-3.04<br>(7.54E-09)
Mtfr2	mitochondrial fission regulator 2	-3.06<br>(4.48E-09)
Sgo2	shugoshin 2	-3.14<br>(3.23E-07)
Ncapg	non-SMC condensin I complex subunit G	-3.19<br>(7.88E-08)
Oip5	Opa interacting protein 5	-3.21<br>(4.08E-09)
Vrk1	vaccinia related kinase 1	-3.21<br>(2.71E-08)
Depdc1	DEP domain containing 1	-3.22<br>(1.87E-08)
Anln	anillin actin binding protein	-3.22<br>(2.22E-08)
Nuf2	NUF2, NDC80 kinetochore complex component	-3.22<br>(4.08E-09)
Ttk	TTK protein kinase	-3.30<br>(8.60E-09)
Iglv2-14	immunoglobulin lambda variable 2-14	-3.31<br>(1.12E-05)
Cenpe	centromere protein E	-3.32<br>(2.84E-09)
Dlgap5	DLG associated protein 5	-3.41<br>(3.02E-09)

Gene symbol	Gene name	Fold change (*P*-value)<br>AAV9-EDI *vs* AAV9-LacZ
Kif11	kinesin family member 11	-3.48<br>(4.08E-09)
Arl6ip1	ADP ribosylation factor like GTPase 6 interacting protein 1	-3.52<br>(5.95E-09)
Spc25	SPC25, NDC80 kinetochore complex component	-3.61<br>(1.19E-06)
Ska1	spindle and kinetochore associated complex subunit 1	-3.65<br>(3.26E-06)
Dtymk	deoxythymidylate kinase	-3.84<br>(2.32E-08)
Ndc80	NDC80, kinetochore complex component	-4.23<br>(3.89E-09)

Supplementary Table 13

Top 100 regulated genes in LS174T cells transduced with LacZ (Con) or EDI. The table refers to the heat map shown in Fig. 5E; microarray gene expression analysis of LS174 cells transduced with LacZ (Con) or EDI (n=3 per condition). The table indicates the gene symbol, gene name and fold-changes and *p*-values; *n*-numbers represent biologically independent experiments. For statistical analysis unpaired two-sided Student's *t*-test was applied. Source data are provided under the following link: http://www.ebi.ac.uk/arrayexpress/experiments/E-MTAB-8111.

Supplementary Table 14
Regulated genes associated with proliferation in LS174T cells transduced with LacZ (Con) or EDI. The table refers to the heat map shown in Fig. 5E

Gene symbol	Gene name	Fold change (*P*-value) AAV9-EDI *vs* AAV9-LacZ
Tspan1	tetraspanin 1	1.64 (1.95E-02)
Traip	TRAF interacting protein	-2.20 (2.76E-06)
Ndrg1	N-myc downstream regulated 1	2.47 (1.33E-05)
Cenpf	centromere protein F	-2.59 1.31E-08
Kif2c	kinesin family member 2C	-2.54 (2.10E-06)
Crip2	cysteine rich protein 2	1.59 (1.58E-02)
Cse1l	chromosome segregation 1 like	-1.53 (6.94E-08)
Dkc1	dyskerin pseudouridine synthase 1	-1.58 (5.66E-07)
Dnmt1	DNA methyltransferase 1	-2.24 (9.14E-07)
Dtymk	deoxythymidylate kinase	-3.84 (2.32E-08)
Ezh2	enhancer of zeste 2 polycomb repressive complex 2 subunit	-1.59 (1.41E-04)
Fabp1	fatty acid binding protein 1	1.75 (6.99E-04)
Tpx2	TPX2, microtubule nucleation factor	-2.22 (1.34E-06)
Foxm1	forkhead box M1	-1.52 (7.87E-04)
Angpt1	angiopoietin 1	1.89 (1.75E-06)
Grk5	G protein-coupled receptor kinase 5	1.58 (1.66E-03)
Hdgf	heparin binding growth factor	-1.93 (1.93E-06)
Birc5	baculoviral IAP repeat containing 5	-2.05 (3.95E-06)

Gene symbol	Gene name	**Fold change (*P*-value)**<br>AAV9-EDI *vs* AAV9-LacZ
Rbpj	recombination signal binding protein for immunoglobulin kappa J region	-1.77<br>(1.33E-05)
Cxcr2	C-X-C motif chemokine receptor 2	1.82<br>(7.34E-03)
Il15	interleukin 15	-1.86<br>(4.58E-04)
Isg20	interferon stimulated exonuclease gene 20	2.03<br>(1.21E-05)
Itga2	integrin subunit alpha 2	1.54<br>(5.87E-03)
Trnp1	TMF1-regulated nuclear protein 1	1.63<br>(8.15E-03)
Mxd1	MAX dimerization protein 1	1.65<br>(7.53E-04)
Mcm7	minichromosome maintenance complex component 7	-1.93<br>(6.07E-06)
Mki67	marker of proliferation Ki-67	-2.07<br>(5.77E-05)
Mre11	MRE11 homolog, double strand break repair nuclease	-1.92<br>(4.66E-05)
Nasp	nuclear autoantigenic sperm protein	-1.95<br>(2.35E-06)
Ceacam6	carcinoembryonic antigen related cell adhesion molecule 6	2.48<br>(2.00E-03)
Hp1bp3	heterochromatin protein 1 binding protein 3	-1.96<br>(2.22E-08)
Sidt2	SID1 transmembrane family member 2	2.34<br>(4.89E-08)
Pcna	proliferating cell nuclear antigen	-1.70<br>(1.36E-04)
Ier5	immediate early response 5	-1.51<br>(6.05E-04)
Plac8	placenta specific 8	1.78<br>(3.91E-03)
Map3k20	mitogen-activated protein kinase kinase kinase 20	-1.65<br>(3.07E-06)
Plk1	polo like kinase 1	-2.29<br>(4.42E-05)

Gene symbol	Gene name	Fold change (*P*-value) AAV9-EDI *vs* AAV9-LacZ
Pola1	DNA polymerase alpha 1, catalytic subunit	-1.55 (3.05E-05)
Tipin	TIMELESS interacting protein	-1.70 (7.63E-05)
Pak1ip1	PAK1 interacting protein 1	-1.58 (6.63E-03)
Phip	pleckstrin homology domain interacting protein	-1.67 (1.62E-05)
Styk1	serine/threonine/tyrosine kinase 1	1.64 (3.23E-04.
Mcm10	minichromosome maintenance 10 replication initiation factor	-2.03 (2.50E-06)
Cdca7l	cell division cycle associated 7 like	-1.57 (1.15E-04)
Prox1	prospero homeobox 1	1.63 (5.50E-04)
Kif15	kinesin family member 15	-2.88 (8.62E-08)
Cnot6	CCR4-NOT transcription complex subunit 6	-1.70 (5.40E-07)
Rac2	ras-related C3 botulinum toxin substrate 2 (rho family, small GTP binding protein Rac2)	1.54 (7.29E-03)
Ccnd1	cyclin D1	-1.57 (2.64E-04)
Reg1b	regenerating family member 1 beta	4.18 (3.47E-07)
Rpa3	replication protein A3	-1.73 (2.05E-05)
Prdm1	PR/SET domain 1	1.53 (4.21E-03)
Stil	SCL/TAL1 interrupting locus	-2.43 (7.06E-05)
Ndrg4	NDRG family member 4	1.63 (2.09E-04)
Skp2	S-phase kinase associated protein 2	-1.50 (1.04E-03)
Brca1	BRCA1, DNA repair associated	-2.26 (2.71E-08)

Gene symbol	Gene name	Fold change (*P*-value) AAV9-EDI *vs* AAV9-LacZ
Stc1	stanniocalcin 1	3.24 (1.08E-08)
Tcf19	transcription factor 19	-1.55 (4.45E-03)
Bub1	BUB1 mitotic checkpoint serine/threonine kinase	-2.45 (5.09E-07)
Bub1b	BUB1 mitotic checkpoint serine/threonine kinase B	-2.48 (5.05E-09)
Tgfbi	transforming growth factor beta induced	1.61 (5.78E-03)
Tgfbr3	transforming growth factor beta receptor 3	-1.62 (4.08E-03)
Tnfrsf1b	TNF receptor superfamily member 1B	1.52 (2.77E-02)
Ttk	TTK protein kinase	-3.30 (8.60E-09)
Ccr2	C-C motif chemokine receptor 2	1.81 (4.36E-05)
E2f8	E2F transcription factor 8	-2.38 (1.58E-06)
Nr4a3	nuclear receptor subfamily 4 group A member 3	1.52 (1.78E-03)
Fam83d	family with sequence similarity 83 member D	-1.82 (1.01E-06)
Cdc7	cell division cycle 7	-2.13 (8.62E-08)
Lgr5	leucine rich repeat containing G protein-coupled receptor 5	-1.61 (1.97E-03)
Ccnb1	cyclin B1	-2.82 (7.49E-08)
Usp13	ubiquitin specific peptidase 13 (isopeptidase T-3)	-1.53 (5.73E-04)
Prc1	protein regulator of cytokinesis 1	-1.99 (1.34E-07)
Aurkb	aurora kinase B	-2.10 (4.11E-06)
Klf4	Kruppel like factor 4	1.57 (2.75E-02)

Gene symbol	Gene name	Fold change (*P*-value) AAV9-EDI *vs* AAV9-LacZ
Lipg	lipase G, endothelial type	-1.68 (3.50E-04)
Kif20b	kinesin family member 20B	-2.04 (4.17E-04)
Dlgap5	DLG associated protein 5	-3.41 (3.02E-09)
Cdk1	cyclin dependent kinase 1	-2.56 (3.68E-10)
Melk	maternal embryonic leucine zipper kinase	-2.02 (4.99E-09)
Cdc20	cell division cycle 20	-2.60 (7.22E-07)
Arnt2	aryl hydrocarbon receptor nuclear translocator 2	1.56 (7.24E-03)
Kif14	kinesin family member 14	-2.99 (6.32E-08)
Cdc25c	cell division cycle 25C	-2.27 (3.15E-04)
Fgfbp1	fibroblast growth factor binding protein 1	1.58 (1.35E-03)

Supplementary Table 14

Regulated genes associated with proliferation in LS174T cells transduced with LacZ (Con) or EDI. The table refers to the heat map shown in Fig. 5E; microarray gene expression analysis of LS174 cells transduced with LacZ (Con) or EDI (n=3 per condition). The table indicates the gene symbol, gene name and fold-changes and *p*-values; *n*-numbers represent biologically independent experiments. For statistical analysis unpaired two-sided Student's *t*-test was applied. Source data are provided under the following link: http://www.ebi.ac.uk/arrayexpress/experiments/E-MTAB-8111.

Supplementary Table 15
Regulated genes associated with cell cycle in LS174T cells transduced with LacZ (Con) or EDI. The table refers to the heat map shown in Fig. 5E.

Gene symbol	Gene name	Fold change (*P*-value) AAV9-EDI *vs* AAV9-LacZ
Rgs2	regulator of G-protein signaling 2	2.43 (6.15E-07)
Trnp1	TMF1-regulated nuclear protein 1	1.63 (8.15E-03)
Nt5e	5'-nucleotidase ecto	1.58 (5.38E-03)
Zwint	ZW10 interacting kinetochore protein	-1.52 (4.56E-05)
Foxm1	forkhead box M1	-1.52 (7.87E-04)
Hace1	HECT domain and ankyrin repeat containing E3 ubiquitin protein ligase 1	-1.53 (1.60E-03)
Erh	enhancer of rudimentary homolog (Drosophila)	-1.54 (6.14E-05)
Lig1	DNA ligase 1	-1.55 (3.19E-03)
Prkcd	protein kinase C delta	-1.55 (1.69E-02)
Suv39h2	suppressor of variegation 3-9 homolog 2	-1.55 (1.32E-03)
Cdt1	chromatin licensing and DNA replication factor 1	-1.56 (7.88E-05)
Ccnd1	cyclin D1	-1.57 (2.64E-04)
Ccne2	cyclin E2	-1.57 (1.60E-02)
Cenps	centromere protein S	-1.64 (2.56E-05)
Rhno1	RAD9-HUS1-RAD1 interacting nuclear orphan 1	-1.64 (7.26E-06)
Ncapd3	non-SMC condensin II complex subunit D3	-1.65 (3.07E-06)
Timeless	timeless circadian clock	-1.65 (1.66E-04)
Kpna2	karyopherin subunit alpha 2	-1.66 (1.18E-04)

Gene symbol	Gene name	Fold change (*P*-value)<br>AAV9-EDI *vs* AAV9-LacZ
Haus4	HAUS augmin like complex subunit 4	-1.66<br>(5.38E-04)
Usp37	ubiquitin specific peptidase 37	-1.66<br>(8.18E-07)
Esco2	establishment of sister chromatid cohesion N-acetyltransferase 2	-1.67<br>(1.46E-03)
Chaf1a	chromatin assembly factor 1 subunit A	-1.68<br>(8.46E-05)
Zwilch	zwilch kinetochore protein	-1.68<br>(4.59E-08)
Cntrl	centriolin	-1.69<br>(1.33E-04)
Katna1	katanin catalytic subunit A1	-1.69<br>(3.17E-05)
Chtf18	chromosome transmission fidelity factor 18	-1.69<br>(1.54E-03)
Tipin	TIMELESS interacting protein	-1.70<br>(7.63E-05)
Chaf1b	chromatin assembly factor 1 subunit B	-1.72<br>(2.08E-05)
Arpp19	cAMP regulated phosphoprotein 19	-1.74<br>(6.57E-05)
Eri1	exoribonuclease 1	-1.75<br>(1.60E-07)
Kntc1	kinetochore associated 1	-1.77<br>(6.14E-06)
Pimreg	PICALM interacting mitotic regulator	-1.78<br>(2.96E-04)
Cenpw	centromere protein W	-1.80<br>(8.96E-06)
Wrn	Werner syndrome RecQ like helicase	-1.80<br>(4.08E-05)
Fam83d	family with sequence similarity 83 member D	-1.82<br>(1.01E-06)
Nup37	nucleoporin 37	-1.85<br>(3.16E-07)
Nedd1	neural precursor cell expressed, developmentally down-regulated 1	-1.87<br>(6.46E-07)

Gene symbol	Gene name	Fold change (*P*-value) AAV9-EDI *vs* AAV9-LacZ
Nup43	nucleoporin 43	-1.89 (5.05E-03)
Cdca5	cell division cycle associated 5	-1.90 (8.04E-06)
Hells	helicase, lymphoid-specific	-1.91 (2.70E-05)
Ncapd2	non-SMC condensin I complex subunit D2	-1.94 (2.41E-05)
Nasp	nuclear autoantigenic sperm protein	-1.95 (2.35E-06)
Aurka	aurora kinase A	-1.96 (1.39E-08)
Smc2	structural maintenance of chromosomes 2	-1.97 (2.42E-07)
Mis18a	MIS18 kinetochore protein A	-1.97 (2.71E-08)
Knstrn	kinetochore localized astrin/SPAG5 binding protein	-1.99 (3.76E-08)
Haus1	HAUS augmin like complex subunit 1	-2.01 (6.00E-05)
Haus7	HAUS augmin like complex subunit 7	-2.02 (8.53E-06)
Cdc6	cell division cycle 6	-2.02 (3.00E-04)
Haus8	HAUS augmin like complex subunit 8	-2.03 (3.26E-06)
Kif20b	kinesin family member 20B	-2.04 (4.17E-04)
Birc5	baculoviral IAP repeat containing 5	-2.05 (3.95E-06)
Kifc1	kinesin family member C1	-2.07 (2.54E-06)
Mki67	marker of proliferation Ki-67	-2.07 (5.77E-05)
Mis18bp1	MIS18 binding protein 1	-2.11 (5.61E-06)
Smc4	structural maintenance of chromosomes 4	-2.13 (3.24E-07)

**Gene symbol**	**Gene name**	**Fold change (*P*-value)** AAV9-EDI *vs* AAV9-LacZ
Rad21	RAD21 cohesin complex component	-2.13 (3.52E-07)
Cdc7	cell division cycle 7	-2.13 (8.62E-08)
Cdca2	cell division cycle associated 2	-2.14 (1.31E-07)
Cdca3	cell division cycle associated 3	-2.14 (1.40E-06)
Cdknn3	cyclin dependent kinase inhibitor 3	-2.15 (2.40E-07)
Kif18b	kinesin family member 18B	-2.16 (1.27E-06)
Spag5	sperm associated antigen 5	-2.18 (9.56E-07)
Lin9	lin-9 DREAM MuvB core complex component	-2.18 (2.45E-06)
Tpx2	TPX2, microtubule nucleation factor	-2.22 (1.34E-06)
Pttg1	pituitary tumor-transforming 1	-2.25 (3.10E-07)
Cdc25c	cell division cycle 25C	-2.27 (3.15E-04)
Mad2l1	mitotic arrest deficient 2 like 1	-2.33 (3.00E-07)
Cenpj	centromere protein J	-2.41 (7.38E-07)
Bub1	BUB1 mitotic checkpoint serine/threonine kinase	-2.45 (5.09E-07)
Ccnb2	cyclin B2	-2.45 (3.70E-07)
Psrc1	proline and serine rich coiled-coil 1	-2.46 (4.88E-05)
Bub1b	BUB1 mitotic checkpoint serine/threonine kinase B	-2.48 (5.05E-09)
Ube2c	ubiquitin conjugating enzyme E2 C	-2.49 (2.08E-07)
Knl1	kinetochore scaffold 1	-2.49 (7.72E-07)

Gene symbol	Gene name	Fold change (*P*-value)<br>AAV9-EDI *vs* AAV9-LacZ
Fbxo5	F-box protein 5	-2.50<br>(7.99E-09)
Mastl	microtubule associated serine/threonine kinase like	-2.53<br>(4.12E-05)
Kif2c	kinesin family member 2C	-2.54<br>(2.10E-06)
Ncapg2	non-SMC condensin II complex subunit G2	-2.54<br>(6.79E-08)
Cdk1	cyclin dependent kinase 1	-2.56<br>(3.68E-10)
Cenpf	centromere protein F	-2.59<br>(1.31E-08)
Nek2	NIMA related kinase 2	-2.60<br>(5.17E-07)
Cdc20	cell division cycle 20	-2.60<br>(7.22E-07)
Bora	bora, aurora kinase A activator	-2.63<br>(6.24E-07)
Ncaph	non-SMC condensin I complex subunit H	-2.70<br>(8.75E-08)
Ccna2	cyclin A2	-2.72<br>(3.57E-10)
Ccnb1	cyclin B1	-2.82<br>(7.49E-08)
Hjurp	Holliday junction recognition protein	-2.88<br>(1.69E-07)
Kif14	kinesin family member 14	-2.99<br>(6.32E-08)
Sgo2	shugoshin 2	-3.14<br>(3.23E-07)
Oip5	Opa interacting protein 5	-3.21<br>(4.08E-09)
Vrk1	vaccinia related kinase 1	-3.21<br>(2.71E-08)
Cenpe	centromere protein E	-3.32<br>(2.84E-09)
Kif11	kinesin family member 11	-3.48<br>(4.08E-09)

Gene symbol	Gene name	Fold change (*P*-value) AAV9-EDI *vs* AAV9-LacZ
Dtymk	deoxythymidylate kinase	-3.84 (2.32E-08)
Ndc80	NDC80, kinetochore complex component	-4.23 (3.89E-09)

Supplementary Table 15

Regulated genes associated with cell cycle in LS174T cells transduced with LacZ (Con) or EDI. The table refers to the heat map shown in Fig. 5E; microarray gene expression analysis of LS174 cells transduced with LacZ (Con) or EDI (n=3 per condition). The table indicates the gene symbol, gene name and fold-changes and *p*-values; *n*-numbers represent biologically independent experiments. For statistical analysis unpaired two-sided Student's *t*-test was applied. Source data are provided under the following link: http://www.ebi.ac.uk/arrayexpress/experiments/E-MTAB-8111.
