# Supplementary material for: Interference with ERK-dimerization at the nucleocytosolic interface targets pathological ERK1/2 signaling without cardiotoxic side-effects
Source: Nat Commun. 2020 Apr 7;11:1733. doi: 10.1038/s41467-020-15505-4 (PMC7138859; doi:10.1038/s41467-020-15505-4)
Supplement: Supplementary file 3 — Reporting Summary [file 41467_2020_15505_MOESM3_ESM.pdf]

nature research

Corresponding author(s): Kristina Lorenz

Last updated by author(s): 08/02/2020

Reporting Summary

Nature Research wishes to improve the reproducibility of the work that we publish. This form provides structure for consistency and transparency in reporting. For further information on Nature Research policies, see Authors & Referees and the Editorial Policy Checklist .

Statistics

For all statistical analyses, confirm that the following items are present in the figure legend, table legend, main text, or Methods section.

n/a	Confirmed	
☐	☒	The exact sample size (n) for each experimental group/condition, given as a discrete number and unit of measurement
☐	☒	A statement on whether measurements were taken from distinct samples or whether the same sample was measured repeatedly
☐	☒	The statistical test(s) used AND whether they are one- or two-sided<br>*Only common tests should be described solely by name; describe more complex techniques in the Methods section.*
☐	☒	A description of all covariates tested
☐	☒	A description of any assumptions or corrections, such as tests of normality and adjustment for multiple comparisons
☐	☒	A full description of the statistical parameters including central tendency (e.g. means) or other basic estimates (e.g. regression coefficient) AND variation (e.g. standard deviation) or associated estimates of uncertainty (e.g. confidence intervals)
☐	☒	For null hypothesis testing, the test statistic (e.g. F, t, r) with confidence intervals, effect sizes, degrees of freedom and P value noted<br>*Give P values as exact values whenever suitable.*
☒	☐	For Bayesian analysis, information on the choice of priors and Markov chain Monte Carlo settings
☒	☐	For hierarchical and complex designs, identification of the appropriate level for tests and full reporting of outcomes
☒	☐	Estimates of effect sizes (e.g. Cohen's d, Pearson's r), indicating how they were calculated

Our web collection on statistics for biologists contains articles on many of the points above.

Software and code

Policy information about availability of computer code

Data collection	Gene expression data was analyzed using Bioconductor 3.1 (http://bioconductor.org); Vevo3100 3.2.0; LAS AF 2.7.9723; Chart5.4, AD Instruments; ImageQuant TL 8.1; qRT-PCR: CFX Manager TM Software Version 3.1
Data analysis	GraphPad Prism 8.0.1, Microsoft Exel 16.16.3; Photoshop 2015.1.2; ImageJ 1.52a, Java 1.8.0_172; ImageQuant TL 8.1; Vevo3100 3.2.0, VevoLab x64 3.2.0; VisualSonics Cardiac Measurements within VevoLab x64 3.2.0; Chart5.4, AD Instruments; G power 3.1.9.3; LAS AF 2.7.9723; R version 3.3.3; Bioconductor Version 3.4; complexHeatmap Version 1.12.0; affyPLM Version 1.50.0; Brainarray mogene20st Version 22.0.0

For manuscripts utilizing custom algorithms or software that are central to the research but not yet described in published literature, software must be made available to editors/reviewers. We strongly encourage code deposition in a community repository (e.g. GitHub). See the Nature Research guidelines for submitting code & software for further information.

Data

Policy information about availability of data

All manuscripts must include a data availability statement. This statement should provide the following information, where applicable:

- Accession codes, unique identifiers, or web links for publicly available datasets
- A list of figures that have associated raw data
- A description of any restrictions on data availability

Gene array data are available from Array Express: accession numbers: E-MTAB-8110 and 8111. Full scans of the blots are available in Supplementary Fig 10. Figures and Extended Data Figures have associated raw data. Raw data for additional information required to interpret, replicate or build upon the findings of this study are available from the corresponding author upon reasonable request.

Field-specific reporting

Please select the one below that is the best fit for your research. If you are not sure, read the appropriate sections before making your selection.

- [x] Life sciences
- [ ] Behavioural & social sciences
- [ ] Ecological, evolutionary & environmental sciences

For a reference copy of the document with all sections, see nature.com/documents/nr-reporting-summary-flat.pdf

Life sciences study design

All studies must disclose on these points even when the disclosure is negative.

Sample size	Sample size for each experiment is indicated in the legend. No statistical tests were used to pre-determine sample size, but sample size was chosen based on previous experiments and comparable studies in literature, which is most optimal to generate statistically significant results. For adequate power, we generally chose a sample size of at least n=6–8 for physiological experiments and at least n=4 for biochemical experiments and n=3-4 for microarray analyses (e.g. doi: 10.1038/nm.3972, doi: 10.1073/pnas.1221999110, doi: 10.1007/s00204-016-1761-4 or doi: 10.1038/nm.1893).
Data exclusions	Mice were excluded from the study if aortic pressure gradient was below 60mmHg to ensure comparable pressure gradients throughout the study. This exclusion criteria was pre-established and was applied in all our former mouse studies involving TAC surgery (e.g. doi: 10.1038/ nm.3972, doi: 10.1073/pnas.1221999110 or doi: 10.1038/nm.1893)
Replication	Not applicable for in vivo experiments. In vitro experiments for screening (Microarray) were performed with biological replicates. For each series of experiments, all attempts at replication were successful. The number of biological samples and independent experiments is stated in the figure legends.
Randomization	Animals and cells were randomly assigned to the experimental groups. Cells were all plated at the same time and wells were randomly selected for treatment with stimuli or inhibitors. Tumor samples were obtained randomly without prior disclosure of the examined parameters.
Blinding	Investigators were blinded to groups allocation during data collection and data analysis as stated in the method section.

Reporting for specific materials, systems and methods

We require information from authors about some types of materials, experimental systems and methods used in many studies. Here, indicate whether each material, system or method listed is relevant to your study. If you are not sure if a list item applies to your research, read the appropriate section before selecting a response.

Materials & experimental systems

n/a	Involved in the study	
[ ]	[x]	Antibodies
[ ]	[x]	Eukaryotic cell lines
[x]	[ ]	Palaeontology
[ ]	[x]	Animals and other organisms
[ ]	[x]	Human research participants
[x]	[ ]	Clinical data

Methods

n/a	Involved in the study	
[x]	[ ]	ChIP-seq
[x]	[ ]	Flow cytometry
[x]	[ ]	MRI-based neuroimaging

Antibodies

Antibodies used

phospho-Akt1(S473) (1:2000, IB; ab81283, abcam), manufactor
phospho-BIM(S69) (1:400, IB; no. 4581, Cell Signaling), manufactor
phospho-Elk1 (S383) (1:1,000, IB; no. 9181, Cell Signaling), manufactor
phospho-Elk1 (S383) (1:1,000, IB; ab34270, abcam), manufactor
phospho-ERK1/2(TEY) (1:1,000, IB; no. 9101L, Cell Signaling), validation by phosphopeptide mapping and by manufactor, doi:10.1038/nm.1893
ERK1/2 (1:1,000, IB; no. 9102, Cell Signaling), manufactor and tested by overexpression, doi:10.1038/nm.1893
phospho-ERK1/2(T188) (1:1,000, IB; 1:500 IHC; Lorenz et al., 2009 or A010-40AP, Badrilla), tested using ERK2 mutant and cometitive phosphopeptide, doi:10.1038/nm.1893
Flag (1:10,000, IB or 1:200, IF, IP; F3165, Sigma,), manufactor and tested by overexpression, doi:10.1038/nm.1893
Gβ (1:5,000, IB; sc-378, Santa Cruz Biotechnology), manufactor and tested using purified Gbg, doi:10.1038/nm.1893
HA (1:5,000, IB; MMS-101R, Covance), manufactor and tested by overexpression, doi:10.1038/nm.1893
HA (1:500, IF; no. 3724, Cell Signaling), manufactor and tested by overexpression
Max (1:200, IF; sc-197, Santa Cruz Biotechnology), manufactor and doi10.1073 071043198

	c-Myc (1:1,000, IB; sc-789, Santa Cruz Biotechnology), manufactor and tested by overexpression<br>c-Myc (1:350, IF; M4439, Sigma), manufactor and tested by overexpression<br>phospho-p90RSK(S380) (1:5,000, IB; ab32203, abcam), manufacturer<br>phospho-Bad(S112) (1:1,000, IB, no. 5284, Cell Signaling), manufacturer and doi: 10.1016/0092-8674(95)90411-5<br>phospho-FoxO3a(S294) (1:1,000, IB; no. 5538, Cell Signaling), manufacturer<br>phospho-p70(S6) (1:1,000, IB; no. 9204, Cell Signaling), manufacturer<br>anti–mouse AffiniPure antibodies (1:10,000, no. 115-035-003), Dianova, Jackson ImmunoResearch, lot 89257, 89918, manufacturer<br>anti-rabbit AffiniPure antibodies (1:10,000, no. 111-035-144), Dianova, Jackson ImmunoResearch, lot 111430, manufacturer<br>anti–goat IgG (1:10,000,sc-2020), Santa Cruz, lot #B0614, manufacturer
Validation	Antibody validation was deferred to the manufacturers and was supported by multiple publications.

Eukaryotic cell lines

Policy information about cell lines

Cell line source(s)	HEK293 (ATCC, CRL-1573), COS7 (ATCC, CRL 1651), LS174T (ATCC, CL188), HT29 (ATCC, HTB38), H9c2 (ATCC, CRL 1446)
Authentication	None of the cell lines used were authenticated
Mycoplasma contamination	None, testings on regular bases
Commonly misidentified lines (See ICLAC register)	No cell line is listed by ICLAC

Animals and other organisms

Policy information about studies involving animals; ARRIVE guidelines recommended for reporting animal research

Laboratory animals	Animals in this study had the genetic backgounds C57BL/6J or FVB/N as indicated. For neonatal rat cardiomyocytes, pups of Sprague Dawley rats (Janvier) were used.<br>For all surgical procedures (transverse aortic constriction, catheterization, AAV-9 injection) male mice at the age of 8weeks were used.<br>For isolation of neonatal rat cardiomyocytes (NRCM) 1-2 day old Sprague Dawley rats were used, both sexes.<br>In all experiments, isogenic, age- and gender- littermates were used as controls.
Wild animals	none
Field-collected samples	none
Ethics oversight	Animal care was performed corresponding to the Committee on Animal Research of the regional government (Regierung von Unterfranken) which reviewed and approved all experimental protocols (Az. 54-2531.01-62/06, Az. 55.2-2531.01-46/09, 20/10, 52/10, 38/11, -60/13 and -42/14 and Az. 81-02.04.2018.A082) according to the national legislation

Note that full information on the approval of the study protocol must also be provided in the manuscript.

Human research participants

Policy information about studies involving human research participants

Population characteristics	Patients undergoing primary resection of a colorectal or lung cancer without prior radio- or radio-chemotherapy were included. Patient age was above 18 years. UICC stage I-IV. Patients with infectious disease (e.g. HIV; Hepatitis) were excluded. All colon cancer samples were adenocarcinoma (G2, pN0-1) and lung samples included adenocarcinomas (G2 and G3, pNx and nN2), a basaloid squamous carcinoma (G3, pN2) and one case of small cell lung cancer. All specimens were derived from primary surgical specimens, without prior treatment.
Recruitment	All patients meeting inclusion criteria were elucidated and written consent was achieved for „biobanking" from resection specimen. No patient refused inclusion.

Ethics oversight

The use of the colon cancer samples was approved by the ethic committee of the University and University Hospital of Würzburg (20180829 01) and all patients gave written consent. Additional colon cancer and lung cancer samples were obtained from RWTH Aachen centralised Biomaterial Bank (RWTH cBMB). The cBMB was reviewed and approved by the Ethics Committee of the Medical Faculty of the RWTH Aachen University. A mandatory prerequisite for incorporation of a biomaterial sample into RWTH cBMB is the written consent of the donor. Before signing, the donor is informed by a medical doctor about the research project and the intended storage of donated samples and associated data. The important contribution of the donor to biomedical research is addressed (quoted from https://www.cbmb.rwth-aachen.de/en/data-privacy). All procedures performed involving human tissue were in accordance with the ethical standards of the institutional research committee which are comparable with the 1964 Helsinki declaration and its later amendments.

Note that full information on the approval of the study protocol must also be provided in the manuscript.
